# Straightforward Syntheses of Alloys and Core–Shell Nanoparticles: The Benefits of Surfactant-Free Alcohol-Mediated Strategies Illustrated with Au–Ag Nanomaterials

**DOI:** 10.1021/acs.nanolett.6c02771

**Published:** 2026-09-17

**Authors:** Julie Agerbo Burslund, Aleksandra Smolska, Javier Eiroa Soto, Xianxian Xie, Kristian Junker Andersen, Armin Asghari Alamdari, Eugenio Solla, Jonathan Quinson

**Affiliations:** † Biological and Chemical Engineering Department, 1006 Aarhus University, 40 Åbogade, Aarhus 8000, Denmark; ‡ CICA-Centro Interdisciplinar de Química e Bioloxía, Facultade de Ciencias, 16737 Universidade da Coruña, Campus de Elviña, 15008 A Coruña, Spain; § Faculty of Mathematics and Physics, Department of Surface and Plasma Science, Charles University, V Holešovičkách 2, Prague 8 18000, Czech Republic; ∥ Servizos de Apoio á Investigación (SAI), 16737 Universidade da Coruña, Campus de Elviña, 15071 A Coruña, Spain

**Keywords:** Nanoparticles, Alloys, Core−shell, Gold, Silver, Monoalcohol, Surfactant-free

## Abstract

Core–shell nanoparticles present unique properties that their monometallic counterparts do not express. Unfortunately, the simple and environmentally friendly preparation of such advanced structures is challenging. A method that bypasses typical challenges in the synthesis of such materials is proposed based on a room-temperature monoalcohol-mediated synthesis without the need for surfactants. The concept is demonstrated with the straightforward synthesis of gold–silver alloys and core–shell nanostructures. The results pave the way for complex materials obtained by more sustainable surfactant-free approaches.

## Graphical abstract

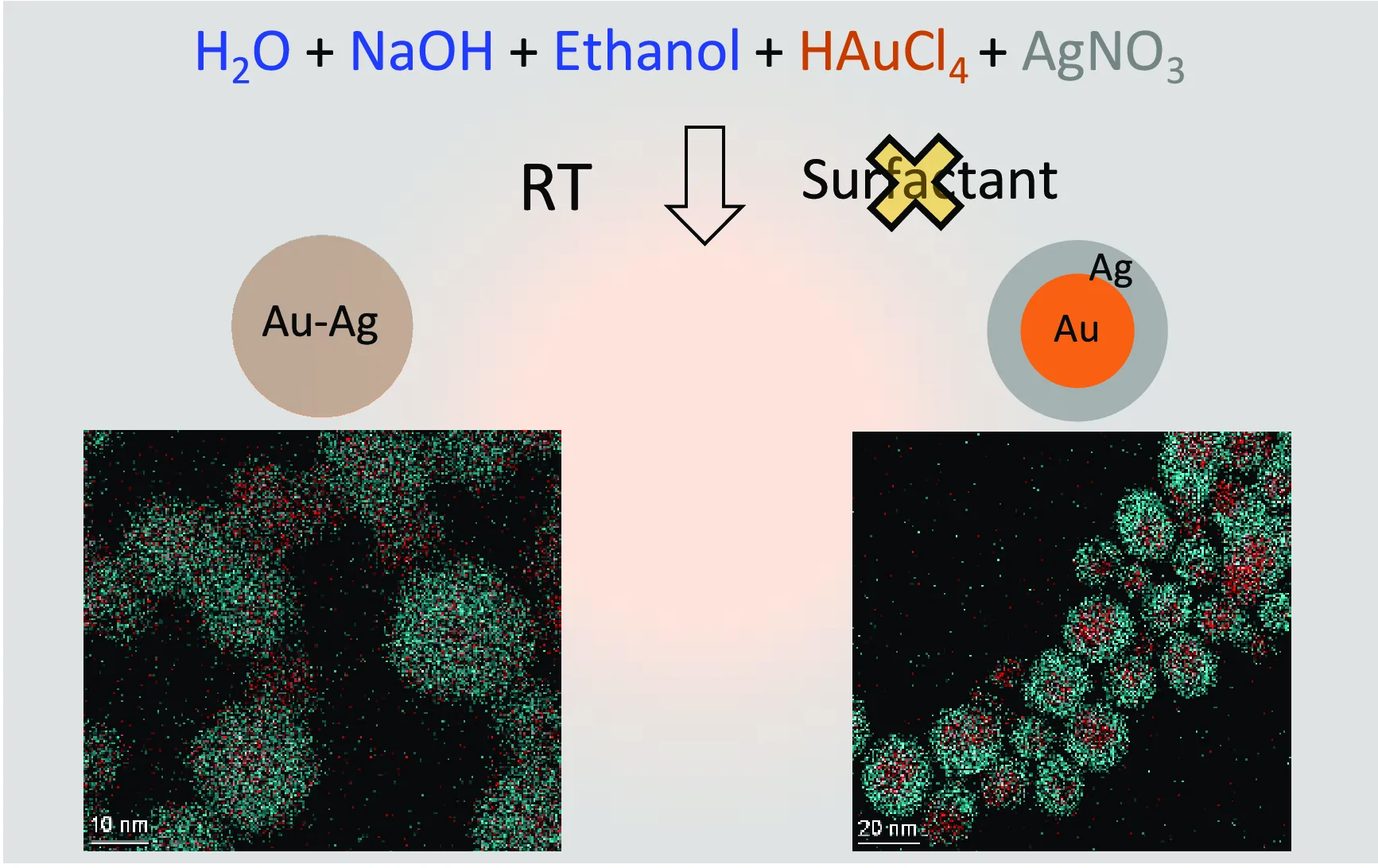

Nanoparticles (NPs) present unique properties relevant for medicine,^1^ sensing,^2^ optics,^3^ electronics,^4^ catalysis,^5^ energy conversion,^6,7^ water or air treatment,^8^ and many more applications. As a result, controlled syntheses of NPs are highly desirable and have been the subject of numerous studies.^9,10^ A remaining challenge in nanotechnology is to identify the right set of experimental synthetic parameters that will lead to a given nanomaterial. An extra degree of complexity comes with the need to design environmentally friendly preparation strategies for nanomaterials to holistically develop more sustainable solutions.^11^ More sustainable strategies have the potential to be widely implemented and bridge the gap between academia and industry.^12^

As an example, gold (Au) and silver (Ag) NPs have been intensively studied because of their potential numerous applications.^13–16^ Colloidal syntheses in the liquid phase are extremely popular due to their relative simplicity. The high redox potentials of Au and Ag precursors allow relatively simple syntheses of Au–Ag alloys, for instance, using room temperature (RT) processes in various solvents, including water.^15,17^ Moreover, due to their pronounced surface plasmon resonance (spr), Au_*x*_Ag_1–*x*_ NPs are easily studied by simple methods such as UV–vis.^17,18^ However, in most cases, a range of additives, capping agents, surfactants, polymers, ligands, etc. are used to stabilize the NPs and/or to achieve size and structure control.^9,19^ Unfortunately, those additives are often derived from fossil fuels, can be toxic, and interact with the NP surface, which can be detrimental for applications where a *clean* surface would be required such as catalysis or medicine. The presence of binding agents can also be detrimental in the development of more complex materials where a *free* surface would be beneficial, e.g., for further functionalization with specific molecules or to develop core@shell NPs.^20^ Removing the additives is often only partial and typically energy demanding and/or generates waste.^5,21,22^

When it comes to developing more sustainable and widely implementable strategies, a popular alternative to potentially harmful chemicals such as NaBH_4_ (playing the role of reducing agent) is to favor biogenic approaches where functional groups from molecules in food or plant extract are relevant sources of reducing agents and/or stabilizers.^23–25^ However, a severe drawback of this approach is that it often requires organic solvents and/or generates waste to produce the extract in the first place. Microbes and fungi are additional sources of reducing agents. However, biogenic species from different parts of the world will have different compositions, which can make a fine control of the NP properties challenging. Finally, the actual amount of biogenic material needed to potentially meet the demand for NPs makes this approach relatively incompatible with large-scale production. Another alternative to obtain surfactant-free materials is to consider laser ablation,^26^ successfully demonstrated to obtain Au, Ag, Au–Ag, and Au@Ag NPs, among many other materials.^26,27^ However, the technique requires relatively specific equipment and some optimization of the laser properties,^1^ which can prevent its widespread use by nonexperts.

To address the challenges related to the use of surfactants and related to the drawbacks of approaches based on biogenic options or laser ablation, surfactant-free syntheses, detailed elsewhere, are a promising option.^28,29^ Among relevant strategies,^26,30–32^ we recently reported a simple synthesis at RT of Au NPs simply obtained using water, NaOH, 20–30 v % ethanol, and HAuCl_4_ as gold precursor, i.e., a simple low-energy process requiring well-defined chemicals and simple molecules.^33,34^ The NPs are stable for months at RT despite the absence of surfactant due to electrostatic stabilization and are relevant for catalysis due to their relatively *clean* surface.^33,35,36^ The alkoxide formed under alkaline conditions likely plays the role of reducing agent.^37^ The synthetic strategy has so far only been explored for palladium (Pd) and Au–Pd alloy materials.^33^ Attempts to transfer the synthesis concepts to silver (Ag) NPs do not lead to well-defined nanomaterials but rather to relatively large materials in the size range of 30–100 nm.^38^

Here, we show that small size Au–Ag as well as core@shell Au@Ag NPs are easily obtained by this monoalcohol-mediated synthesis. To the best of our knowledge, this is the first time that a surfactant-free bottom-up colloidal approach is reported to prepare core@shell materials. Previous work using polyols such as glycerol was encouraging, but all used PVP and/or a support.^37,39,40^ Polyols are not ideal due to their higher viscosity and challenging handling.^41^ We attribute the scarcity of reports regarding surfactant-free core@shell materials to the general scarcity of robust protocols leading in the first place to surfactant-free monometallic NPs.^5,42^ Indeed, it is widely believed that additives, polymers, and surfactants are *needed* to achieve colloidal stability and prepare such materials. Therefore, the field of nanomaterial synthesis by bottom-up colloidal strategies remains therefore largely dominated by the use of such chemicals.^22^ Here, the *free* surface of the Au core and the excess of ethanol needed to prepare the Au NPs are leveraged to build up a shell at RT without the need to add an extra reducing agent.

The first finding is that mixing Au and Ag precursors in an alkaline solution of ethanol and water at RT easily leads to bimetallic Au–Ag NPs or Au@Ag NPs. The general scheme of the synthesis is presented in Figure 1a, and examples of UV–vis spectra of Au, Ag, Au–Ag, and Au@Ag NPs are reported in Figure 1b with illustrative STEM-EDS micrographs in Figure 1c,d. The typical spr signal of Au–Ag materials is observed^17^ in-between the expected signal of Au and Ag NPs with a maximum at 520 nm and 400–500 nm, respectively; see also Section S2 in the Supporting Information (SI). STEM-EDS confirms the presence of Au and Ag in the NPs, for instance, a shell made of Ag; see Figure S4. The formation of a crystalline core and shell is further confirmed by HRTEM; see Figure S5. The shell is rather homogeneous based on STEM-EDS, and the overall coverage is comparable to what has been reported for Au@Ag obtained by alternative methods, where part of the shell tends to be typically a bit thicker on one side of the NP.^18,43–51^ It is documented that in Au@Ag NPs a charge transfer from the Au core to the Ag shell maintains the Ag shell in a rather reduced state.^51–53^ However, a mixed state of Ag can be expected due to the relatively simple oxidation of Ag, especially when the Ag shell thickness increases.^49,53,54^ The relative amount of Au to Ag is controlled by the relative amount of precursor used as detailed in Section S3.

**Figure 1:**
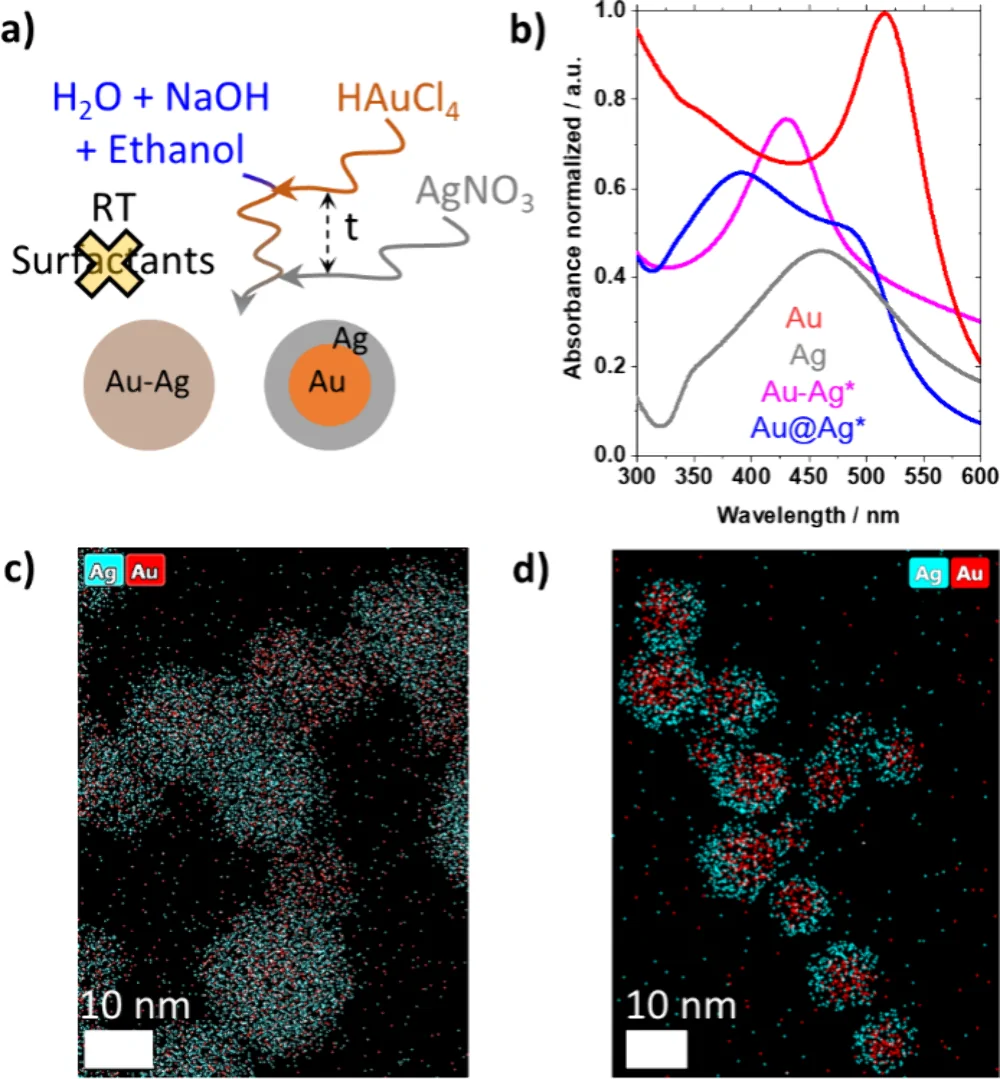
(a) Schematic of the room temperature (RT) alcohol-mediated surfactant-free synthesis of Au–Ag and Au@Ag NPs. (b) Illustrative UV–vis spectra of Au, Au–Ag, Au@Ag, and Ag NPs obtained by the approach using ethanol as the source of reducing agent, as indicated. *The spectra for Au–Ag and Au@Ag NPs were recorded on solutions diluted 4×. The absorbance is normalized to the maximum of the data set. (c, d) Illustrative STEM-EDS data colored maps of (c) Au–Ag and (d) Au@Ag NPs.

The results of a large parametric study are reported in the SI; see Section S4. A parameter to carefully tune for a successful synthesis is the time of addition of the silver precursor; see Section S5. Indeed, premixing HAuCl_4_ and AgNO_3_ in water leads to the formation of a AgCl precipitate. An option to bypass this challenge would be the coinjection of the precursors^17^ or the use of additives,^37^ but these options were not investigated here to keep the process as simple to perform as possible. In order to form mixed Au–Ag structures or Au@Ag, the timing of a delayed and sequential addition of the precursors is key. Section S4 discusses in detail the influences of the nature of the alcohol, the Au:Ag ratio, the ethanol content, the base concentration, and the time of addition of AgNO_3_ on the resulting NPs. The mixed Au–Ag structures are obtained by the rapid addition of the precursors (e.g., with a 20 s interval). A preferred strategy here is to add the Au precursor first, motivated by the fact that preparing Ag NPs does not lead to stable materials.^38^ Indeed, the NPs obtained when AgNO_3_ is added first tend to lead to phase-separated Janus-like or patchy-like material with poorly controlled shape attributed to galvanic displacement; see Figure S8.

As the time between the addition of HAuCl_4_ and AgNO_3_ increases, Au@Ag NPs were obtained. This is arguably expected since the Au NPs have more time to form.^55^ A benefit of delaying the addition of AgNO_3_ is to better control the synthesis as detailed in the SI. However, it cannot be guaranteed that the kinetics of formation is exactly the same every time. This pushed us to consider the addition of AgNO_3_ to preformed Au NPs. An important finding is that NPs with well-defined spr are obtained when an extra amount of base is also added; see Section 5.2 in the Supporting Information. This is attributed to the need to form new alkoxides from the alcohol in excess to be able to more efficiently reduce the second precursor.^37^

The results presented in Figure 2, Figure 3, and Section S6 and detailed below confirm the successful formation of Au@Ag NPs. It is established that the preformed surfactant-free NPs in ethanol-mediated syntheses are *alive* in the sense that (i) the reducing ability of the as-prepared solution can be reactivated by simply adding more base and (ii) the absence of strongly binding capping agents (otherwise typically used in the literature^5,56^ ) makes it possible to further use the seeds to build up more complex materials. Both features lead to a process that favors the formation of a shell over the formation of Ag NPs or patchy Au–Ag structures. Interestingly, the synthesis in aqueous media is here performed at RT because there is no need to compete with the usual ligand binding and no need to favor their desorption at higher temperatures, which also contributes to the formation of a shell compared to nucleation of new Ag NPs. Without base added, the shell is not formed, which confirms the expected role of alkoxides as a source of reducing agent.^37^ Furthermore, the synthesis is faster and more controlled if the AgNO_3_ is added first; see Figure S29. While a full investigation of the reason behind this observation is beyond the scope of this first study, it can be related to the expected formation of AgOH (poorly soluble in water) in the presence of base (i.e., when adding AgNO_3_ to an already alkaline solution).

**Figure 2:**
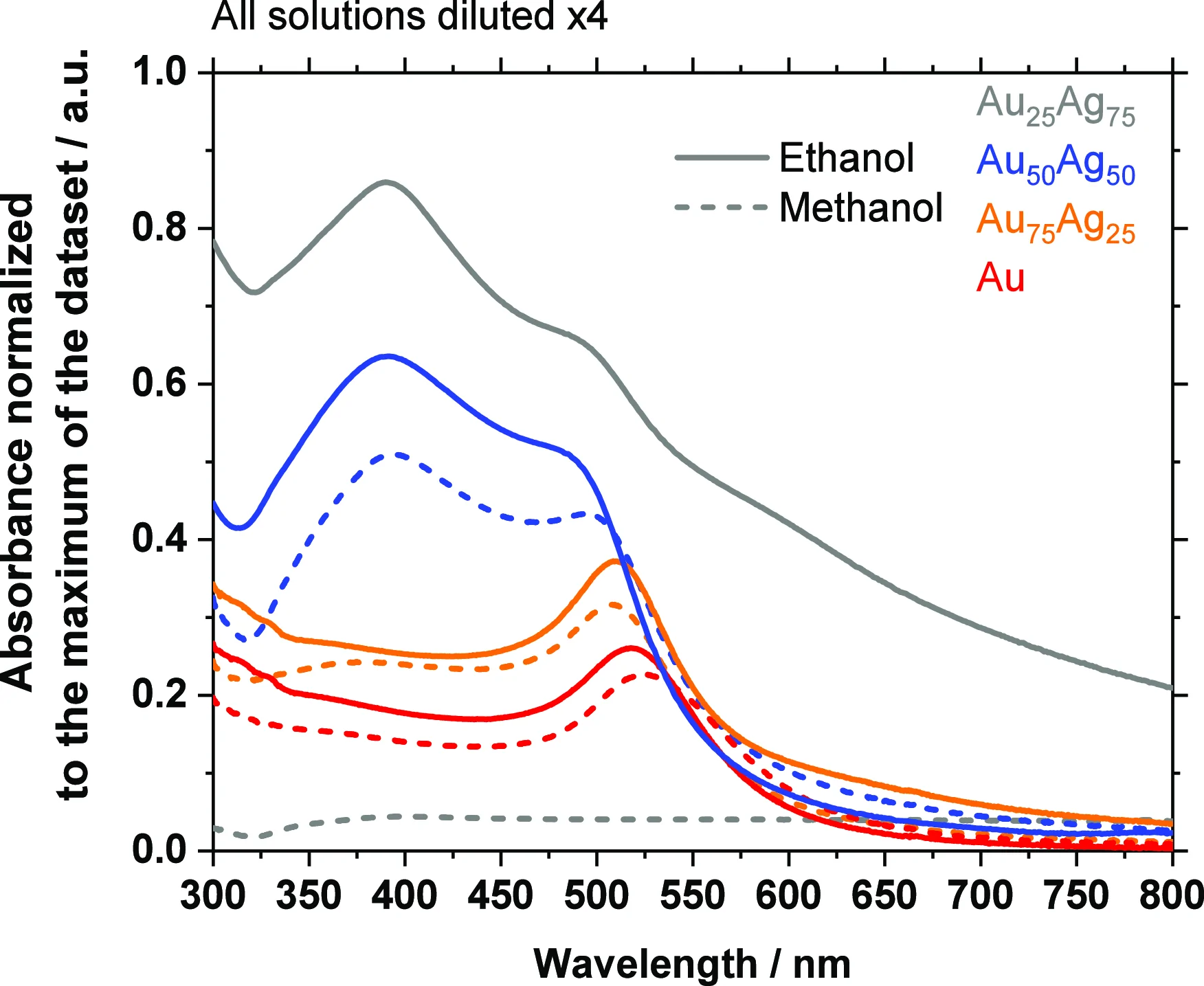
Illustrative UV–vis spectra of the Au@Ag NPs seeds prepared using 0.5 mM_Au_, overall 4 mM NaOH and 30 vol % alcohol using ethanol and methanol for different Au:Ag molar ratios of 75:25, 50:50, or 25:75, as indicated. NaOH was added in two steps equivalent to 2 mM each, once for the formation of the Au core and once for the formation of the shell. AgNO_3_ was added first and then NaOH, after 1 min. All solutions of as prepared materials were diluted 4× for measurement.

**Figure 3:**
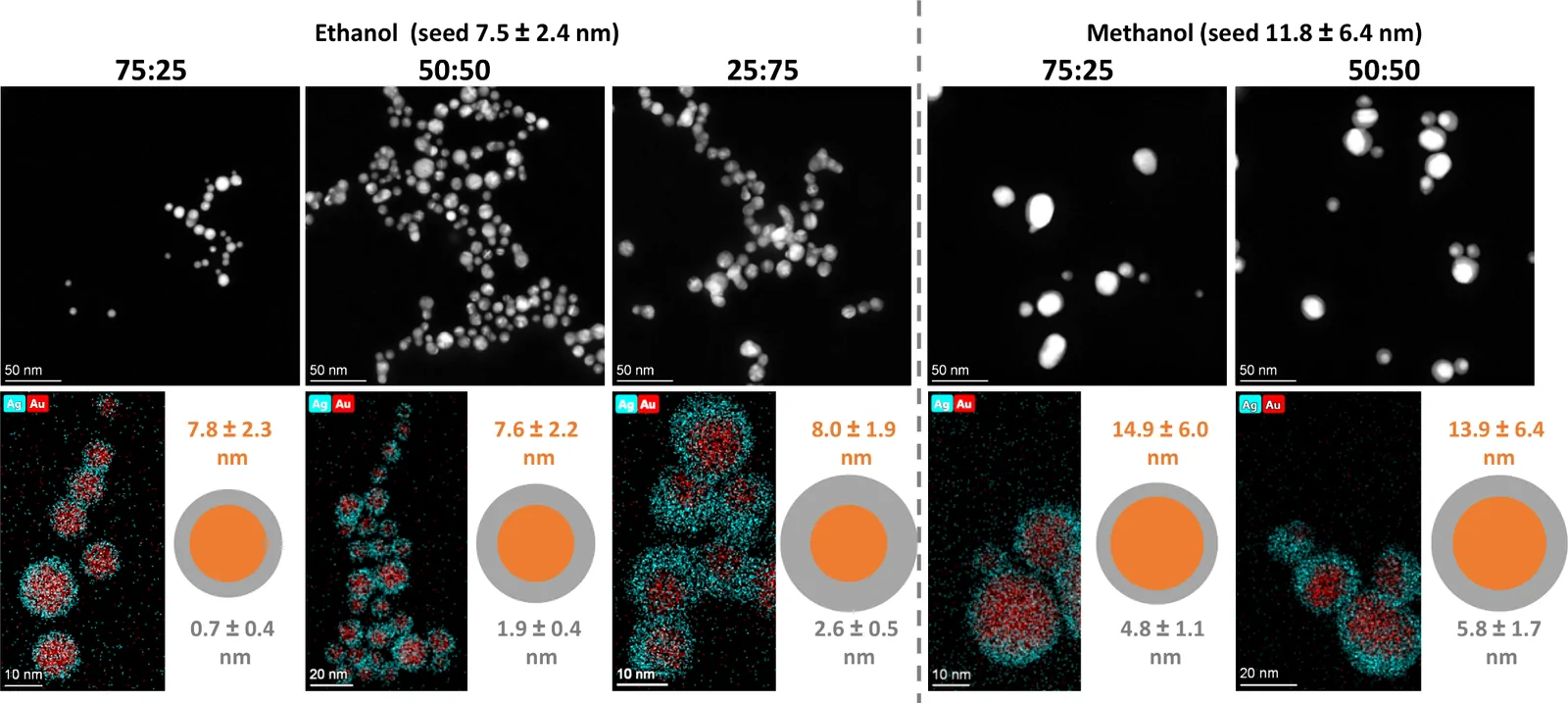
Illustrative STEM-EDS micrographs and schematic illustration of Au@Ag NP structures obtained using overall 4 mM NaOH and 30 vol % alcohol using ethanol or methanol for different Au:Ag molar ratios of 75:25, 50:50, or 25:75, as indicated. The corresponding UV–vis spectra are given in Figure 2 and complementary data, in Section S6 of the Supporting Information.

A strong benefit of this approach is that the size of Au NPs can be easily tuned by using ethanol or methanol,^33^ mixtures of alcohols,^33,55^ various NaOH/Au molar ratios,^33^ HAuCl_4_ or HAuBr_4_ as precursor,^57^ light,^34^ or water conductivity,^35^ and the seeds are obtained at a relatively high concentration of 0.5 mM_Au_ in volumes as large as 1 L^33^ and are stable for years even at ambient temperature.^36^ This means that it is relatively simple to control the size of the core. By simply adding more silver relative to the gold, the size of the shell can be tuned as well, as illustrated in Figure 2 and Figure 3 for various Au@Ag nanomaterials with various plasmomic properties related to various core and shell sizes.^18^

Interestingly, while ethanol-mediated syntheses lead to rather large and poorly defined Ag NPs^38^ (see in particular Figure S6), the strategy to aim for core@shell structures overall leads to NPs expressing a rather homogeneous Ag shell on NPs with a controlled overall diameter below 30 nm, illustrated in Figure 3. In other words, an Ag shell is obtained on NPs with an overall much smaller diameter than one can be obtained for monometallic Ag NPs. The strategy presented here is therefore promising for developing surfactant-free Ag surfaces on smaller nanomaterials by an ethanol-mediated synthesis.

The colloids are stable for several weeks with only minor changes as detailed in Section S7. It is important to weigh the achievements above considering that no surfactants are used, only simple equipment is needed for the synthesis, and only a minimal prior knowledge in nanomaterial preparation or a very basic knowledge in chemical sciences is required. It can be pointed out that the samples are not always as monodispersed as in more conventional surfactant-assisted syntheses. While this can be a drawback for some studies, this also allows one to investigate size-dependent trends within the same sample. For instance, we could observe that in nonoptimized syntheses the larger NPs tend to be more Ag-poor or Ag-rich; see, for example, Figures S7 or S26. The samples are therefore ideal for further studies to correlate the size and composition at the nanoscale. It is noteworthy to mention that the absence of surfactant facilitates imaging by electron microscopy; see, for instance, Figure S5.^18,41,58^

Finally, the surfactant-free NPs are promising, readily active catalysts. It is a general challenge to obtain Ag structures by the ethanol-mediated synthesis^38^ but not Au@Ag NPs. This allows us to first dive into the differences of Au or Ag NPs of similar sizes. Using the NaBH_4_-mediated reduction of methylene blue (MB) dye as a first example,^59^ it is shown that the formation of the Ag surface drastically modifies the kinetics of the reduction compared to the Au core, as detailed in Section S8. A second illustration in Figure 4 is the 4-nitrophenol (4-NP) reduction.^60^ Without incentive to optimize the process at this stage, the Au@Ag NPs with nominal composition 50:50 (Au:Ag) and obtained with Au NP seeds prepared in ethanol show a turnover frequency of around 130 h^–1^ which is higher than the ca. 50 h^–1^ for Ag NPs obtained by a surfactant-free alcohol-mediated synthesis that leads to Ag NPs ca. 45 nm in size at best in an optimal version using ethylene glycol as the source of the reducing agent.^38^ The higher activity achieved here is attributed to the smaller size of the Au@Ag NPs around 10 nm compared to what can be obtained by a surfactant-free alcohol-mediated approach for Ag NPs. Finally, the surfactant-free NPs are also readily electro-active (see Section S9), and their electrochemical characterization confirm further the formation of a Ag shell on the Au NPs. Those preliminary results illustrate the benefits of a core@shell strategy to develop surfactant-free Ag-based nanomaterials, for instance, toward catalytic applications.

**Figure 4:**
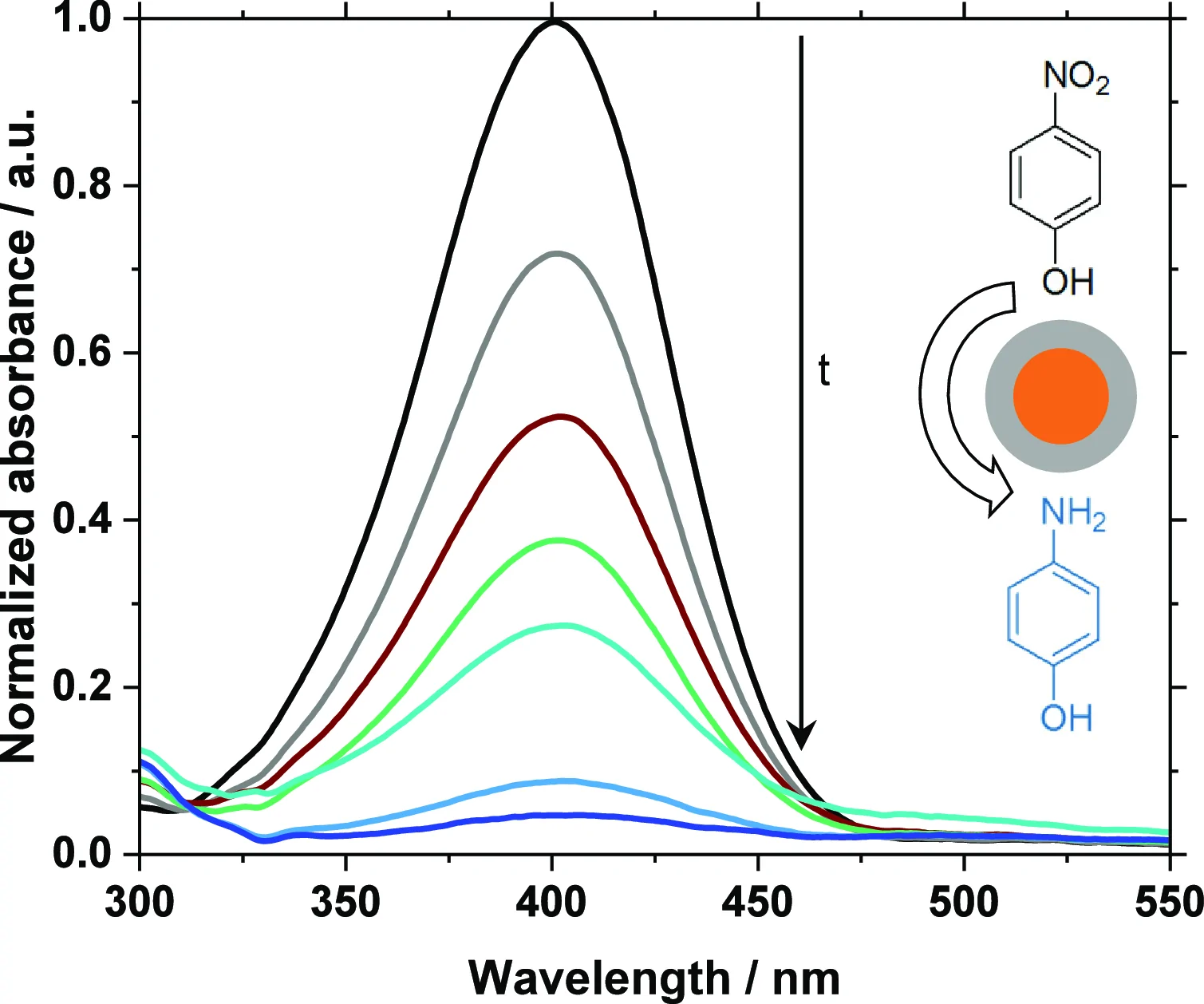
Illustrative time-resolved UV–vis spectra of 4-nitrophenol reduction catalyzed by Au@Ag NPs.

In conclusion, it is shown for the first time to the best of our knowledge that stable surfactant-free colloidal Au–Ag and Au@Ag NPs can be obtained at RT using an alcohol-mediated approach. It is shown that adding NaOH to seeds prepared in monoalcohol and water mixtures can reactivate the reducing ability of the excess alcohol making the prepared surfactant-free NP convenient building blocks toward increasingly more complex materials. The sizes of the core and the shell can be controlled. The relative simplicity of the approach is especially well-suited to comply with the increasing need to design sustainable investigations in nanotechnology. The proposed synthesis approach is anticipated to significantly facilitate a range of investigation on the formation and properties of Au@Ag NPs; the concept could be relevant to grow a Ag shell on other Au NPs, for instance, with different shapes^61–64^ and/or inspire the development of other metallic materials relevant for catalysis, sensing, optics, or medicine.^16,65–67^

## Supplementary Material



## Notes

Further methodology and UV–vis, STEM, and catalysis data (PDF)

## References

[1] Kalus M. R., Rehbock C., Barsch N., Barcikowski S. (2017). Colloids created
by light: Laser-generated nanoparticles for applications in biology
and medicine. Materials Today-Proceedings.

[2] Tabatabaei M. S., Islam R., Ahmed M. (2021). Applications
of gold nanoparticles
in ELISA, PCR, and immuno-PCR assays: A review. Anal. Chim. Acta.

[3] Singla S., Muskan, Lal B., Kaur J., Sharma G. (2024). Optical behavior
of glasses containing
gold nanoparticles: A review. Optics and Laser
Technology.

[4] Hossain N., Mobarak M., Mimona M., Islam M., Hossain A., Zohura F., Chowdhury M. (2023). Advances and significances of nanoparticles
in semiconductor applications - A review. Results
in Engineering.

[5] Quinson J., Kunz S., Arenz M. (2023). Surfactant-free colloidal
syntheses
of precious metal nanoparticles for improved catalysts. ACS Catal..

[6] Seh Z. W., Kibsgaard J., Dickens C. F., Chorkendorff I. B., Norskov J. K., Jaramillo T. F. (2017). Combining theory and experiment in
electrocatalysis: Insights into materials design. Science.

[7] Schroder J., Pittkowski R. K., Martens I., Chattot R., Drnec J., Quinson J., Kirkensgaard J. J. K., Arenz M. (2022). Tracking the Catalyst
Layer Depth-Dependent Electrochemical Degradation of a Bimodal Pt/C
Fuel Cell Catalyst: A Combined Operando Small- and Wide-Angle X-ray
Scattering Study. ACS Catal..

[8] Chaudhary S., Sharma P., Chauhan P., Kumar R., Umar A. (2019). Functionalized
nanomaterials: a new avenue for mitigating environmental problems. International Journal of Environmental Science and Technology.

[9] Daruich
De Souza C., Ribeiro Nogueira B., Rostelato M. E. C.M. (2019). Review
of the methodologies used in the synthesis gold nanoparticles by chemical
reduction. J. Alloys Compd..

[10] Cargnello M. (2019). Colloidal
Nanocrystals as Building Blocks for Well-Defined Heterogeneous Catalysts. Chem. Mater..

[11] Freese T., Elzinga N., Heinemann M., Lerch M. M., Feringa B. L. (2024). The relevance
of sustainable laboratory practices. RSC Sustainability.

[12] Quinson J. (2025). Questioning
Established Protocols for New and More Sustainable Syntheses of Nanomaterials. Langmuir.

[13] Qin L., Zeng G. M., Lai C., Huang D. L., Xu P. A., Zhang C., Cheng M., Liu X. G., Liu S. Y., Li B. S. (2018). ″Gold rush″ in modern science: Fabrication
strategies and typical advanced applications of gold nanoparticles
in sensing. Coord. Chem. Rev..

[14] Garcia A. G., Lopes P. P., Gomes J. F., Pires C., Ferreira E. B., Lucena R. G. M., Gasparotto L. H. S., Tremiliosi-Filho G. (2014). Eco-friendly
synthesis of bimetallic AuAg nanoparticles. New J. Chem..

[15] Bhattarai B., Zaker Y., Bigioni T. P. (2018). Green synthesis of gold and silver
nanoparticles: Challenges and opportunities. Current Opinion in Green and Sustainable Chemistry.

[16] Awiaz G., Lin J., Wu A. (2023). Recent advances
of Au@Ag core-shell SERS-based biosensors. Exploration.

[17] Larm N. E., Thon J. A., Vazmitsel Y., Atwood J. L., Baker G. A. (2019). Borohydride
stabilized gold-silver bimetallic nanocatalysts for highly efficient
4-nitrophenol reduction. Nanoscale Adv..

[18] Hazra A., Hossain S., Pramanick A., Ray M. (2025). Spontaneous Interfacial
Alloying in Au@Ag Core@Shell Nanoparticles: Unraveling the Impact
on Plasmonic Absorption. Journal Of Physical
Chemistry C.

[19] Pinto V., Ferreira M., Silva R., Santos H., Silva F., Pereira C. (2010). Long time effect on the stability of silver nanoparticles
in aqueous medium: Effect of the synthesis and storage conditions. Colloids Surf., A.

[20] Ghosh
Chaudhuri R., Paria S. (2012). Core/Shell Nanoparticles: Classes,
Properties, Synthesis Mechanisms, Characterization, and Applications. Chem. Rev..

[21] Cargnello M., Chen C., Diroll B. T., Doan-Nguyen V. V. T., Gorte R. J., Murray C. B. (2015). Efficient Removal of Organic Ligands
from Supported Nanocrystals by Fast Thermal Annealing Enables Catalytic
Studies on Well-Defined Active Phases. J. Am.
Chem. Soc..

[22] Rossi L. M., Fiorio J. L., Garcia M. A. S., Ferraz C. P. (2018). The role and fate
of capping ligands in colloidally prepared metal nanoparticle catalysts. Dalton Transactions.

[23] Khan T., Ullah N., Khan M. A., Mashwani Z. U. R., Nadhman A. (2019). Plant-based
gold nanoparticles; a comprehensive review of the decade-long research
on synthesis, mechanistic aspects and diverse applications. Adv. Colloid Interface Sci..

[24] Akhtar M. S., Panwar J., Yun Y. S. (2013). Biogenic
Synthesis of Metallic Nanoparticles
by Plant Extracts. ACS Sustainable Chem. Eng..

[25] Ortiz-Tirado A., Medina-Ganem L., Bandala E., Conejo-Davila A., Vega-Rios A., Goonetilleke A., Rodriguez-Narvaez O. (2025). Biogenic nanoparticles:
Synthesis, characterization, applications and scaling up limitations
in water treatment. Journal of Environmental
Chemical Engineering.

[26] Reichenberger S., Marzun G., Muhler M., Barcikowski S. (2019). Perspective
of Surfactant-free Colloidal Nanoparticles in Heterogeneous Catalysis. ChemCatChem..

[27] Omar G., Abd Ellah R., Elzayat M., Afifi G., Imam H. (2024). Superior removal
of hazardous dye using Ag/Au core-shell nanoparticles prepared by
laser ablation. Optics and Laser Technology.

[28] Quinson J. (2021). Surfactant-free
precious metal colloidal nanoparticles for catalysis. Frontiers in Nanotechnology.

[29] Israelachvili, J. N. Intermolecular and Surface Forces, 3rd ed.; Academic Press, 2011; ISBN 978-0-12-391927-4.

[30] Faraday M. (1857). The Bakerian
Lecture: Experimental relations of gold (and other metals) to light. Philosophical Transactions of the Royal Society of London.

[31] Kawasaki H., Yamamoto H., Fujimori H., Arakawa R., Iwasaki Y., Inada M. (2010). Stability of the DMF-Protected
Au Nanoclusters: Photochemical, Dispersion,
and Thermal Properties. Langmuir.

[32] Wang Y., Ren J. W., Deng K., Gui L. L., Tang Y. Q. (2000). Preparation
of tractable platinum, rhodium, and ruthenium nanoclusters with small
particle size in organic media. Chem. Mater..

[33] Quinson J., Aalling-Frederiksen O., Dacayan W., Bjerregaard J., Jensen K., Jorgensen M., Kantor I., Sorensen D., Kuhn L., Johnson M. (2023). Surfactant-Free Colloidal
Syntheses of Gold-Based Nanomaterials in Alkaline Water and Mono-alcohol
Mixtures. Chem. Mater..

[34] Røjkjær
Rasmussen D., Lock N., Quinson J. (2025). Lights on the Synthesis
of Surfactant-Free Colloidal Gold Nanoparticles in Alkaline Mixtures
of Alcohols and Water. ChemSusChem.

[35] Jæger F., Pedersen A. A., Wacherhausen P. S., Smolska A., Quinson J. (2025). Surfactant-free
gold nanoparticles synthesized in alkaline water-ethanol mixtures:
leveraging lower grade chemicals for size control of active nanocatalysts. RSC Sustainability.

[36] Varga M., Quinson J. (2025). Fewer, but better: on the benefits for surfactant-free
colloidal syntheses of nanomaterials. ChemistrySelect.

[37] Gomes J. F., Garcia A. C., Ferreira E. B., Pires C., Oliveira V. L., Tremiliosi-Filho G., Gasparotto L. H. S. (2015). New insights into the formation mechanism
of Ag, Au and AgAu nanoparticles in aqueous alkaline media: alkoxides
from alcohols, aldehydes and ketones as universal reducing agents. Phys. Chem. Chem. Phys..

[38] Sivebæk
Lind L. H., Smolska A., Eiroa Soto J., Quinson J. (2026). Surfactant-free room temperature syntheses of stable
colloidal silver nanoparticles in alkaline water-alcohols mixtures:
pitfalls and opportunities. Nanoscale Adv..

[39] Garcia A., Caliman J., Ferreira E., Tremiliosi-Filho G., Linares J. (2015). Promotional Effect of Ag on the Catalytic
Activity
of Au for Glycerol Electrooxidation in Alkaline Medium. ChemElectroChem..

[40] Gomes J., Garcia A., Pires C., Ferreira E., Albuquerque R., Tremiliosi-Filho G., Gasparotto L. (2014). Impact of the AuAg NPs Composition
on Their Structure and Properties: A Theoretical and Experimental
Investigation. J. Phys. Chem. C.

[41] Quinson J., Nielsen T. M., Escudero-Escribano M., Jensen K. M. Ø. (2023). Room temperature
syntheses of surfactant-free colloidal gold nanoparticles: the benefits
of mono-alcohols over polyols as reducing agents for electrocatalysis. Colloids Surf., A.

[42] Kouawa
Fokam H., Smolska A., Quinson J. (2026). NaBH4-mediated syntheses
of colloidal gold nanocatalysts in water: are additives really needed?. Journal of Materials Chemistry A.

[43] Gao C., Hu Y., Wang M., Chi M., Yin Y. (2014). Fully Alloyed Ag/Au
Nanospheres: Combining the Plasmonic Property of Ag with the Stability
of Au. J. Am. Chem. Soc..

[44] Wang K., Sun D., Pu H., Wei Q. (2019). Shell thickness-dependent Au@Ag nanoparticles
aggregates for high-performance SERS applications. Talanta.

[45] Sirgedaite G., Talaikis M., Drabavicius A., Niaura G., Mikoliunaite L. (2025). Synthesis
and characterization of Au@Ag nanoparticles for multiwavelength SERS
biosensing. Spectrochimica Acta Part A-Molecular
and Biomolecular Spectroscopy.

[46] Loiseau A., Zhang L., Hu D., Salmain M., Mazouzi Y., Flack R., Liedberg B., Boujday S. (2019). Core-Shell
Gold/Silver
Nanoparticles for Localized Surface Plasmon Resonance-Based Naked-Eye
Toxin Biosensing. ACS Appl. Mater. Interfaces.

[47] Paital D., Sen T., Patra A., Haldar K. (2017). Core size matters! High Raman enhancing
core tunable Au/Ag bimetallic core-shell nanoparticles. Gold Bulletin.

[48] Lei H., Zhao W., Huang F., Tian Y., Zhao Q., Ma R., Ren L., Li H., Dou S., Wang Z. (2024). Shell thickness-dependent
Au@Ag nanoparticles for surface-enhanced Raman scattering detection
of pollutants. Vacuum.

[49] Knauer A., Eisenhardt A., Krischok S., Koehler J. (2014). Nanometer precise adjustment
of the silver shell thickness during automated Au-Ag core-shell nanoparticle
synthesis in micro fluid segment sequences. Nanoscale.

[50] Yang L., Yan W., Wang H., Zhuang H., Zhang J. (2017). Shell thickness-dependent
antibacterial activity and biocompatibility of gold@silver core-shell
nanoparticles. RSC Adv..

[51] Kamimura S., Yamashita S., Abe S., Tsubota T., Ohno T. (2017). Effect of
core@shell (Au@Ag) nanostructure on surface plasmon-induced photocatalytic
activity under visible light irradiation. Appl.
Catal., B.

[52] Nishimura S., Dao A. T. N., Mott D., Ebitani K., Maenosono S. (2012). X-ray Absorption
Near-Edge Structure and X-ray Photoelectron Spectroscopy Studies of
Interfacial Charge Transfer in Gold-Silver-Gold Double-Shell Nanoparticles. J. Phys. Chem. C.

[53] Thi
Ngoc Anh D., Singh P., Shankar C., Mott D., Maenosono S. (2011). Charge-transfer-induced suppression of galvanic replacement
and synthesis of (Au@Ag)@Au double shell nanoparticles for highly
uniform, robust and sensitive bioprobes. Appl.
Phys. Lett..

[54] Maenosono S., Lee J., Dao A. T. N., Mott D. (2012). Peak shape analysis of Ag 3d core-level
X-ray photoelectron spectra of Au@Ag core-shell nanoparticles using
an asymmetric Gaussian-Lorentzian mixed function. Surf. Interface Anal..

[55] Hamer
Larsen J. C., Porsgaard A. N., Vinding L., Smolska A., Quinson J. (2025). Less chemicals for more controlled syntheses: on the
benefits of mixtures of alcohols for room temperature surfactant-free
colloidal syntheses of gold nanoparticles. RSC
Sustainability.

[56] Seifert J., Traoré N., Diesch J., Kichigin A., Nees N., Peukert W., Spiecker E., Zubiri B., Pflug L., Taylor R. (2025). Silver Incorporation
into Gold Patchy Nanoparticles:
Influence on the Tunability and Long-Term Stability of Plasmonic Properties. J. Phys. Chem. C.

[57] Sorensen M., Fokam H., Smolska A., Larsen J., Nordhal G., Bojesen E., Lock N., Quinson J. (2026). Room temperature
surfactant-free
syntheses of colloidal gold nanoparticles: Comparison of the precursors
HAuCl_4_ and HAuBr_4_. Colloids
Surf., A.

[58] Li C., Tardajos A., Wang D., Choukroun D., Van Daele K., Breugelmans T., Bals S. (2021). A simple method to
clean ligand contamination on TEM grids. Ultramicroscopy.

[59] Nathawat P., Sahu M., Ganguly M. (2026). Role of sodium
borohydride as a co-catalyst
for nanoparticle-induced degradation of toxic dyes in aquatic systems. Nanoscale Adv..

[60] Smolska A., Lock N., Quinson J. (2026). On the Overlooked Effect of Counter-Cations
From Borohydrides for the Gold- and Metal- Mediated Catalytic Reduction
of 4-Nitrophenol. Small Methods.

[61] Serrano-Freijeiro A., Zarzuela-Amor M., Renero-Lecuna C., Obelleiro-Liz M., Martin V., Perez-Juste I., Taboada J., Casado J., Pastoriza-Santos I., Perez-Juste J. (2026). Chiral Au@Ag Core-Shell
Nanoparticles for Enantioselective SERS Detection of Bio-Relevant
Chiral Molecules. ACS Nanoscience Au.

[62] Jamali S., Ul Abideen Z., Khaskheli M., Abro M., Malik M., Akram M., Ali S. (2026). Au@Ag nano cuboids with tunable surface
plasmon resonance: A pathway to high-performance and chemically stable
SERS substrates. Physica B-Condensed Matter.

[63] Sha P., Su Q., Dong P., Wang T., Zhu C., Gao W., Wu X. (2021). Fabrication
of Ag@Au (core@shell) nanorods as a SERS substrate by
the oblique angle deposition process and sputtering technology. RSC Adv..

[64] Ma Y., Li W., Cho E., Li Z., Yu T., Zeng J., Xie Z., Xia Y. (2010). Au@Ag Core-Shell Nanocubes with Finely Tuned and Well-Controlled
Sizes, Shell Thicknesses, and Optical Properties. ACS Nano.

[65] Quinson J., Dworzak A., Simonsen S. B., Kuhn L. T., Jensen K. M. O., Zana A., Oezaslan M., Kirkensgaard J. J. K., Arenz M. (2021). Surfactant-free synthesis of size
controlled platinum
nanoparticles: Insights from in situ studies. Appl. Surf. Sci..

[66] Yusuf B., Yaseen W., Xie J., Babangida A., Muhammad A., Xie M., Xu Y. (2022). Rational design of
noble metal-based multimetallic nanomaterials: A review. Nano Energy.

[67] Loza K., Heggen M., Epple M. (2020). Synthesis, Structure,
Properties,
and Applications of Bimetallic Nanoparticles of Noble Metals. Adv. Funct. Mater..

