# Supplementary material for: Straightforward Syntheses of Alloys and Core–Shell Nanoparticles: The Benefits of Surfactant-Free Alcohol-Mediated Strategies Illustrated with Au–Ag Nanomaterials
Source: Nano Lett. 2026 Sep 17;26(38):12795–801. doi: 10.1021/acs.nanolett.6c02771 (PMC13632581; doi:10.1021/acs.nanolett.6c02771)
Supplement: Supplementary file 1 [file nl6c02771_si_001.pdf]

Supporting information for

Straightforward syntheses of alloys and core-shell nanoparticles: the benefits of surfactant-free alcohol-mediated strategies illustrated with Au-Ag nanomaterials

Julie Agerbo Burslund,[a] Aleksandra Smolska,[a] Xianxian Xie,[a,b] Kristian Junker Andersen,[a] Armin Asghari Alamdari,[a] Eugenio Solla,[c] Jonathan Quinson[d,a*]

a) Biological and Chemical Engineering Department, Aarhus University, 40 Åbogade, Aarhus, Denmark
b) Faculty of Mathematics and Physics, Department of Surface and Plasma Science, Charles University, V Holešovičkách 2, Prague 8, 18000, Czech Republic
c) Servizos de Apoio á Investigación (SAI), Universidade da Coruña, Campus de Elviña, 15071, A Coruña, Spain
d) CICA-Centro Interdisciplinar de Química e Bioloxía, Facultade de Ciencias, Universidade da Coruña, Campus de Elviña, 15008, A Coruña, Spain

j.quinson@udc.es

Table of contents

1. Materials and methods 3
1.1. Chemicals 3
1.2. Synthesis of Au-Ag NPs 3
1.3. Characterization 6
1.3.1. UV-vis 6
1.3.2. STEM-EDS 6
1.3.3. HRTEM 8
1.4.3. Zeta potential 8
1.4. Catalysis 9
1.4.1. 4-nitrophenol reduction 9
1.4.2. MB reduction 9
1.4.3. Metrics 9
1.5. Electrochemical characterization and electrocatalysis 10
2. UV-vis Au, Ag and Au-Ag 11
3. Composition control 12
4. Notes on parametric study 13
5. Delayed addition of $HAuCl_4$ and $AgNO_3$ 15
5.1. Short delay 15
5.1.1. Influence of the alcohol 15
5.1.2. Influence of the Au:Ag ratio 17
5.1.3. Influence of the ethanol content 19
5.1.4. Influence of the base 20
5.2. Long delay 21
5.2.1. Sequential addition of the precursors 21
5.2.2. Scale up in concentration and sequential addition of the base 24
6. Core@Shell 29
7. Stability 37
8. Catalysis 38
9. Electrochemical characterization 42
10. References 44

1. Materials and methods

1.1. Chemicals

All chemicals were used as received: $HAuCl_4$ ($HAuCl_4 \cdot 3H_2O$, ACS grade, Sigma-Aldrich); $AgNO_3$ (ACS reagent, ≥ 99.0 %, Sigma Aldrich); methanol (AnalaR NORMAPUR, Reag. Ph. Eur. ACS, VWR); ethanol (EtOH, Absolute, VWR); ethylene glycol (EG, EMSURE®, Reag. Ph. Eur. Reag. USP, Supelco®); glycerol (bi-distilled 99.5%, VWR); NaOH (Reag. Ph. Eur., VWR); KOH (Reag. Ph. Eur., VWR) ; water (mQ, Milli-Q,Millipore, resistivity of >18.2 MΩ•cm); H_2SO_4 ((99.999%, Sigma-Aldrich); HCl (puriss. ACS reagent, reag. ISO, Reag. Ph. Eur. fumin, ≥ 37%, Sigma Aldrich); HNO_3 (puriss ≥ 65%, Sigma Aldrich); 4-nitrophenol (4-NP, ReagentPlus®, ≥99%, Sigma-Aldrich); $NaBH_4$ (ReagentPlus®, 99%, Sigma-Aldrich); methylene blue (MB, FluoroChem); N_2 (99.999%).

1.2. Synthesis of Au-Ag NPs

General. The NPs were prepared using stock solutions of $HAuCl_4$, $AgNO_3$ and NaOH in water at 50 mM. The stock solutions of $HAuCl_4$ and $AgNO_3$ were prepared in glass containers and stored in aluminum foil and in a fridge. The stock of NaOH was prepared in a plastic container and stored at ambient temperature.[1] The relative amount of $HAuCl_4$ or $AgNO_3$ was tuned to obtain various Au:Ag molar ratios and the total concentration of metal, as indicated. All syntheses were carried out in rectangular disposable polystyrene UV-vis cuvettes (1 cm wide) equipped with a magnetic stir bar (both flushed with compressed air prior to adding liquid). The stir bars were cleaned using *aqua regia* (4:1 v:v, concentrated HCl:HNO_3) and washed thoroughly with mQ water (***to be handled and disposed of following with extra care the safety regulations at the work place***).

Au or Ag NPs. The reaction was initiated by adding the $HAuCl_4$ precursor (alternatively $AgNO_3$) to a solution containing water, the alcohol and the base, under stirring. The cuvettes were then capped and shaken once manually before being placed again rapidly under stirring conditions. The total volume was 2 mL. For the first two hours, the reactions took place in a photobox (Puluz LED portable photo studio, PU5060EU, 60 cm x 60 cm x 60, 60 W) to control the light[2] while stirring (500 rpm) using a magnetic stirrer and then were left overnight at ambient temperature and light without stirring before removing the magnet and performing UV-vis characterization and/or preparing the grids for STEM-EDS analysis. The samples were then closed further with Parafilm and stored at ambient temperature in a storage box. The whole synthesis was conducted at room temperature (ca. 22 °C).
Note that although this was not used here, a simple manual shake (without magnetic stirring) is possible to obtain Au NPs by this approach which alleviate from the use of *aqua regia*.[3]
Au-Ag NPs. The Au-Ag NPs were obtained by adding $AgNO_3$ 20 seconds after the $HAuCl_4$. Shorter times between the additions of the two precursor are possible but sometimes challenging to

reproduce. 20 seconds were identified as the shortest time that could reproducibly and safely be achieved by a single experimentalist (with the need to switch from the Au stock solution to Ag stock solution). The procedure is schematized in **Figure S1**. Note that the use of two pipettes for a co-injection is an option[4] but we here focus on a methodology that can be widely implemented with the minimum of specific equipment to democratize the use of bi-metallic NPs.

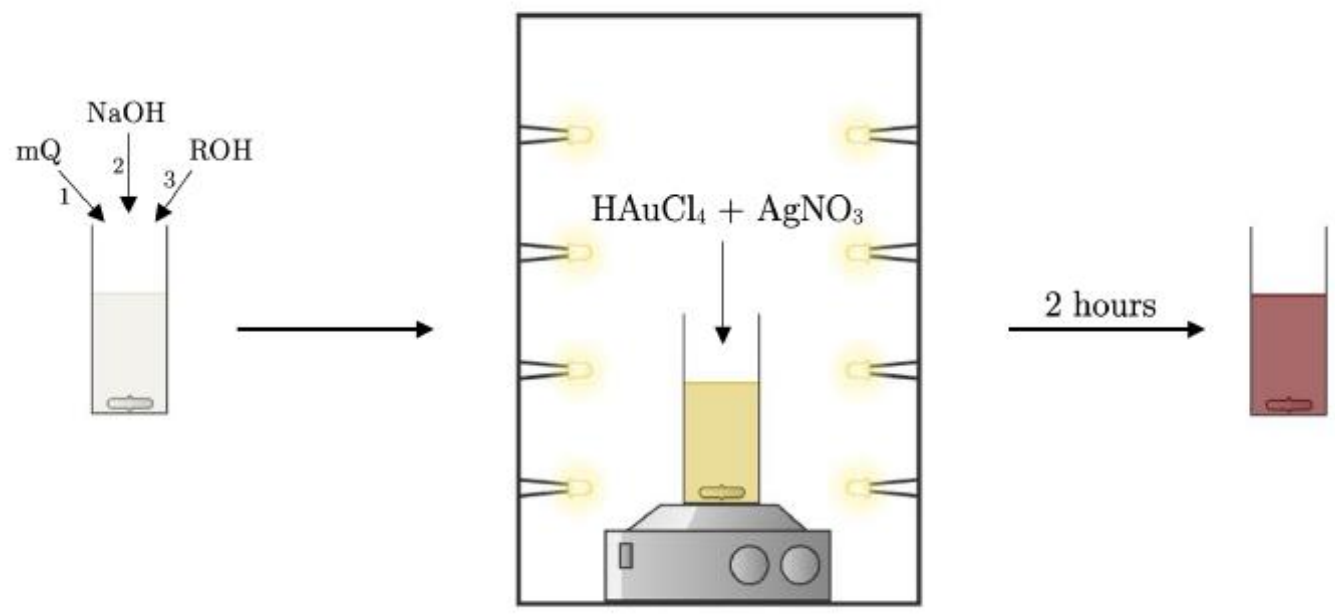

Figure S1. Schematic representation of a synthesis based on a sequential but fast addition of $HAuCl_4$ then $AgNO_3$. The addition of the precursors is performed in a photobox and the sample left to react under stirring and in the photobox for 2 hours. The time delay between the additions of $HAuCl_4$ and $AgNO_3$ is within few seconds and typically 20 seconds.

Delayed addition. In a range of syntheses, a longer delayed addition compared to the case presented above was performed. In some cases, the total amount of base was not added from the beginning of the synthesis but after the addition of $HAuCl_4$ or $AgNO_3$, as detailed and illustrated in **Figure S2**.

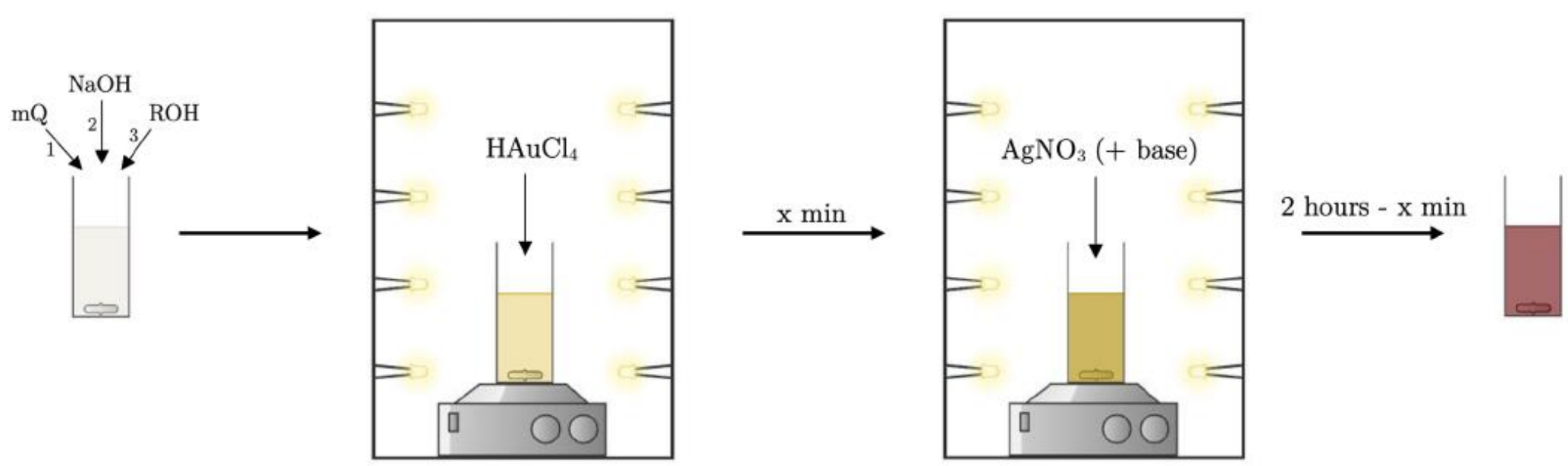

Figure S2. Schematic representation of a synthesis based on a sequential addition of $HAuCl_4$ and $AgNO_3$. The addition of the precursors is performed in a photobox and the sample left to react under stirring and in the photobox for 2 hours. The time delay between the additions of $HAuCl_4$ and $AgNO_3$ is controlled to be several minutes, as indicated.

Au@Ag NPs. To obtain core-shell NPs, pre-made Au NPs obtained following the procedure detailed above and in the literature were used,[2,3,5] and prepared first without adding $AgNO_3$. $AgNO_3$ was added to those NPs as illustrated in **Figure S3**. Specifically here, the pre-made NPs (seeds) were prepared with 0.5 mM $HAuCl_4$, 2 mM NaOH and 30 v.% ethanol or methanol for a volume of 100 mL and 14 mL, respectively. 2 mL of seeds were used to which the relevant chemicals were added under stirring. The concentrations expressed for those samples are considering a total volume of 2 mL given the very small volume of $AgNO_3$ or NaOH added (typically 15-60 µL and 80 µL, respectively).

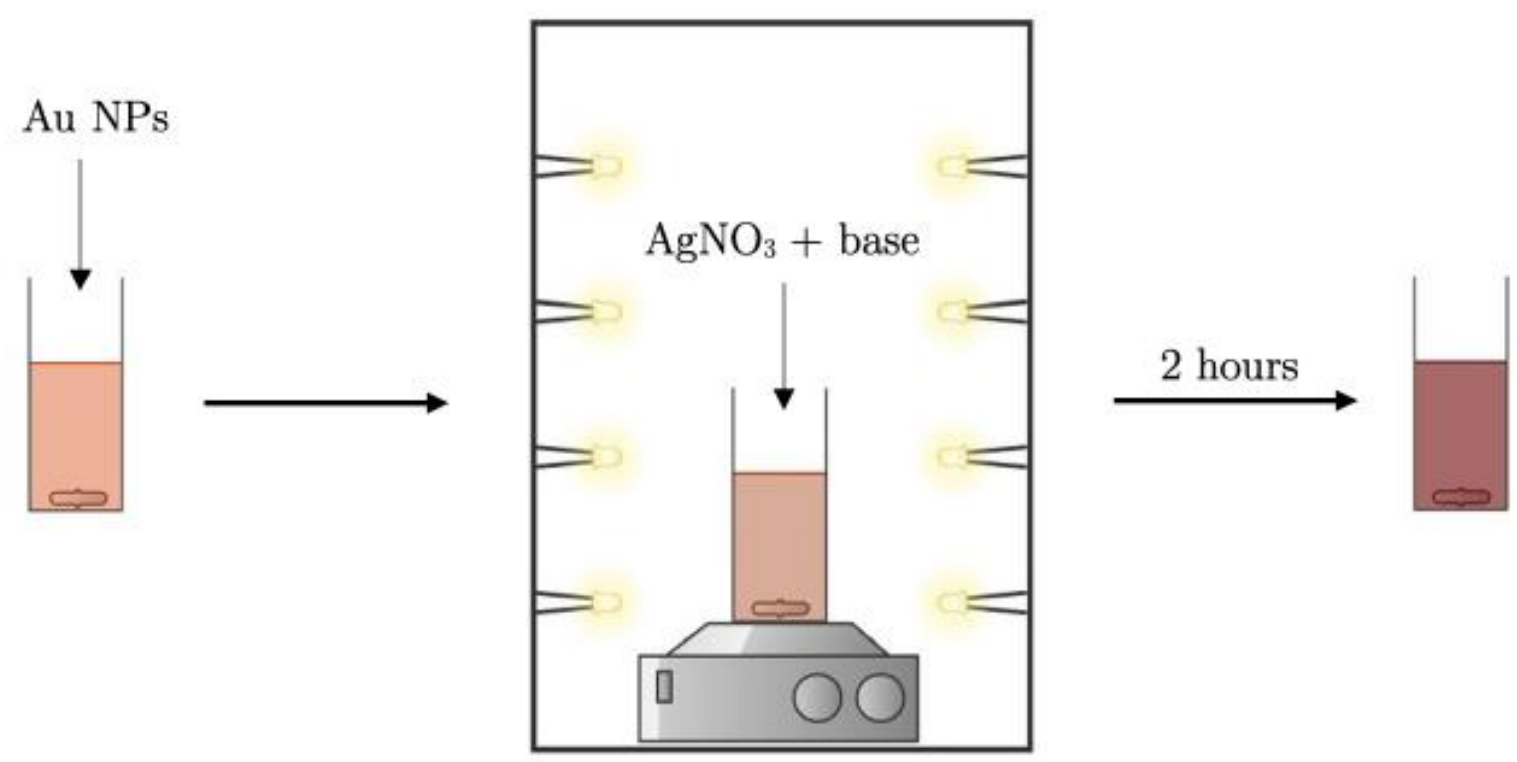

Figure S3. Schematic representation of a synthesis based on the addition of $HAuCl_4$ on pre-formed Au NPs. The addition of the precursors is performed in a photobox and the sample left to react under stirring and in the photobox for 2 hours.

Note on reproducibility. Since reproducibility can be a challenge in the nano-sciences,[6,7] we made sure that the successful synthesis of Au@Ag NPs could be achieved using different batches of Au NPs (e.g. prepared with 20 or 30 v.% ethanol, several months old), could be performed by various experimentalists and could be performed in different laboratories in different countries (Denmark and Spain). The successful synthesis was achieved in all cases.

Equations. The use of ethanol for the ethanol-mediated synthesis is detailed in previous work.[8] By analogy it is expected that the overall reduction of the Ag in alkaline media can be described as:

$$AgNO_3 + 1\ e^- \rightarrow Ag^0 + NO_3^-$$
$$H_3CCH_2OH + 2\ OH^- \rightarrow H_3CCOH + 2\ H_2O + 2\ e^-$$

$$H_3CCH_2OH + 2\ AgNO_3 + 2\ OH^- \rightarrow H_3CCOH + 2\ H_2O + 2\ Ag^0 + 2\ NO_3^-$$

1.3. Characterization

1.3.1. UV-vis

UV-vis spectrum were recorded 24 hours after synthesis using a UV spectrophotometer (Thermo Scientific Genesys 10S) with a scanning interval of 300-800 nm. A reference (blank) consisted in the same type and amount of alcohol in water as the respective sample.

1.3.2. STEM-EDS

Micrographs from scanning transmission electron microscopy (STEM) and maps from energy dispersive X-ray spectroscopy (EDS) were obtained on a FEI Talos F200X. Different detectors (bright field and high angle annual dark field) were used to obtain the micrographs. The samples were prepared by depositing the as-prepared colloidal on a Cu grids and washing with water before drying at ambient temperature. The size of the Au-Ag particles where assessed based on the STEM data whereas the relative composition were obtained from the EDS maps using the L-lines of Au and Ag, see **Figure S4**. The NP sizes were obtained using the image processing software ImageJ for a number of NPs as indicated.

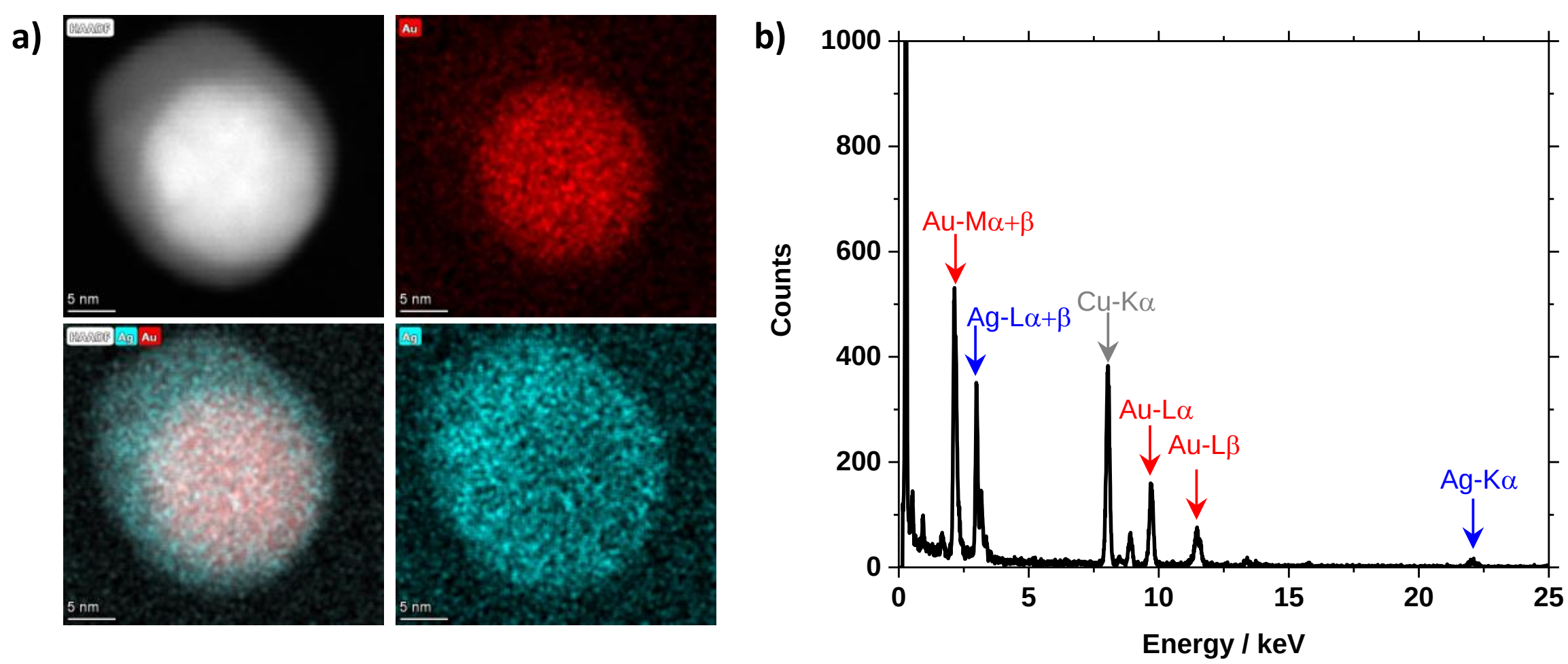

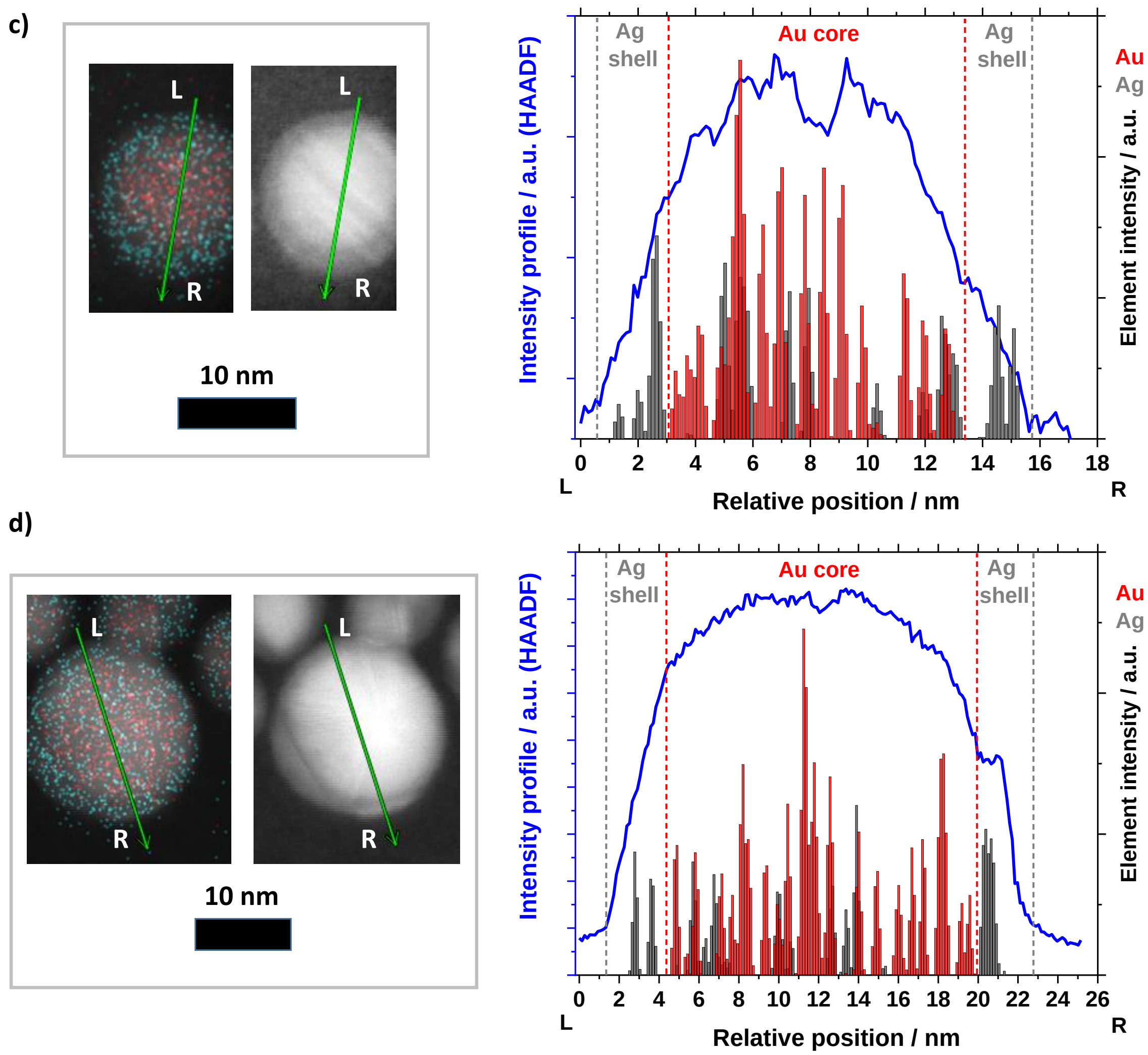

Figure S4. (a) Illustrative STEM-EDS data for a Au@Ag NP and (b) corresponding spectrum. (c,d) Single NP EDS profile across a NP showing the related micrographs (left) and the corresponding profiles (right). In the EDS maps Au is in red and Ag in blue, as in (a).

The results in **Figure S4**, clearly show a shell with only Ag detected that also corresponds rather well to the High-Angle Annular Dark-Field detector (HAADF) intensity profile, despite the relatively thin shell obtained here.

1.3.3. HRTEM

High resolution transmission microscopy (HRTEM) was performed with a Jeol JEM2010HR operating at 200 kV and the grids prepared as described above.

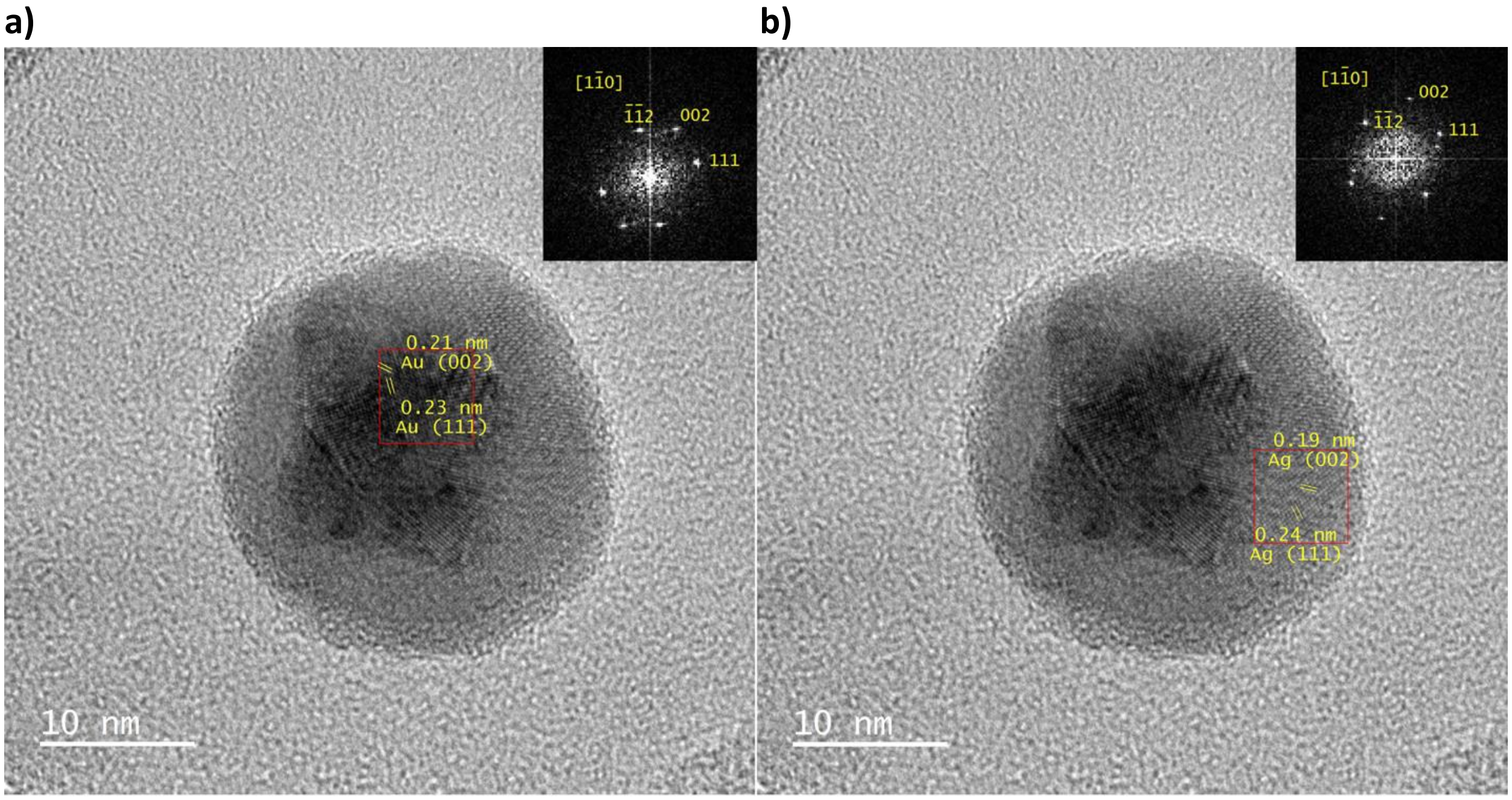

Figure S5. Illustrative HRTEM micrograph of the same Au@Ag NP in (a) and (b).

In **Figure S5a**, the phase determination for the Au core is presented, and in **Figure S5b** for the Ag shell, together with the Fast the Fast Fourier Transform (FFT) calculated on the corresponding squared areas (red square). On the core of the particle, the crystal planes for Au (002) and (111) at 0.21 and 0.23 nm respectively, were recorded. When indexing the FFT for the core, a positive match for an Au cell viewed along the [1-10] zone axis was obtained. The same analysis was applied to the Ag shell, where the (002) and (001) planes at 0.19 and 0.24 nm respectively, were recorded. The FFT obtained on the area marked by the red square on the shell, also positively matched with an Ag cell viewed along the [1-10] zone axis. FFT patterns derived from the HRTEM images were indexed using standard FCC unit cells for Au and Ag, retrieved from the Crystallography Open Database (COD ID No. 9008463 for Au and COD ID No. 9008459 for Ag).[9]

1.4.3. Zeta potential

A Zetasizer Pro (Malvern Panalytical) equipment was used using a refractive index of gold NPs of 0.18 and an absorption of 3.433. The solutions were measured in a dedicated cell (Disposable Folded Capillary Cell, DTS1070).

1.4. Catalysis

1.4.1. 4-nitrophenol reduction

The reduction of 4-NP was performed following our recently reported procedure,[10] and general guidelines.[11] The experiment was performed directly in a UV-vis cuvette placed in a UV-vis spectrophotometer (Vernier, Go Direct® UV-vis spectrophotometer) under stirring. The measurements then began immediately after, and spectra were collected every 20 sec. The UV-vis spectrophotometer was calibrated using a blank consisting of mQ water. A stock solution of $NaBH_4$ at 100 mM freshly prepared was used together with a 1 mM stock solution of 4-NP. A given volume of as-prepared colloids (typically fews µL depending on the total concentration of metal in the dispersion) was added to 0.1 mL of 4-NP at 1 mM mixed with 0.1 mL of of $NaBH_4$ at 100 mM. Total volume of the aqueous solution was 2 mL. This leads to a final concentration of typically 0.00125 mM_{Au+Ag}, 0.05 mM of 4-NP and 5 mM of $NaBH_4$, therefore a final $NaBH_4$/4-NP molar ratio of 100 and a [Ag+Au]/4-NP molar percentage of 2.5%. The decrease of the absorbance at 400 nm was followed over time to establish the reduction of 4-NP.[11] ***Pay attention on how to discard the $NaBH_4$ and 4-NP based waste following the good practices implemented in the laboratory***.

1.4.2. MB reduction

The MB reduction was performed in similar way to the 4-NP reduction but a Go Direct® SpectroVis® Plus Spectrophotometer was used. The main difference was that a constant flow of N_2 was used during the reduction and no stirring was performed. The stock solutions were 1 mM MB in water and freshly prepared 100 mM $NaBH_4$. The as-prepared colloids were diluted in water to 0.125 mM_{Au} or 0.25 mM_{Au+Au}, as indicated. The final concentrations in a reaction volume of 2 mL were 0.05 mM of MB, 10 mM of $NaBH_4$, 0.625 µM of Au or Ag when only one metal was used and 1.25 μM_{Au+Ag} otherwise. The $NaBH_4$/MB molar ratio was therefore 200, i.e. a large excess of $NaBH_4$. The Au/MB percentage was 1.25%. After mixing the water, MB and $NaBH_4$, the solution was purged with N_2 for 3 minutes before the catalyst was added (Au NPs, Au@Ag NPs, $HAuCl_4$, $AgNO_3$ or mixtures, as indicated).

1.4.3. Metrics

The turnover frequency (TOF) were estimated using the relationship:

$$TOF = \frac{C_{dye\_0}}{C_{metal}} . \frac{A_0 - A_t}{A_0} . \frac{1}{t}$$

where A_t is the absorbance of characteristic peaks of 4-NP (400 nm) or MB (662 nm) at a reaction time t and A_0 is the initial absorbance. C_{dye_0} is the initial concentration of dye and C_{metal} the overall concentration of metal used. Note that other metrics could report a normalization by mass and/or active surface size of metal given that nanomaterials are here used.

The apparent rate constant k_{app} was estimated assuming a pseudo-first-order kinetics:[11,12]

$$t.k_{app} = -\ln(\frac{A_t}{A_0})$$

Note that:

$$TOF = \frac{C_{dye-0}}{C_{metal}}.(1 - e^{-t.k_{app}}).\frac{1}{t}$$

1.5. Electrochemical characterization and electrocatalysis

The electrochemical characterization was performed following the procedure previously reported and detailed elsewhere.[3] Briefly, the measurements were performed in a three-electrode set up consisting of a working electrode (WE) that is a glassy carbon (GC) electrode with a diameter of 5 mm (0.2 cm_2), a counter electrode (CE) that was a graphite rod (Thermo Scientific Chemicals, 6.15 mm x 152 mm, 15403735) and the reference electrodes were a Saturated Calomel Electrode (SCE, SI Analytics™ ScienceLine Glass Single Reference Electrode, Fisher Scientitic, 10542113) or a Hg/HgO (Origalys, OGR001) electrode, as indicated.

To prepare the WE, 30 µL of the as-prepared dispersions of Au NPs (0.5 mM Au) or Au@Ag NPs (0.5 mM_{Au} and 0.5 mM_{Ag}) were dropped on the GC surface (polished to mirror finish using aluminum oxide paste, 1 and 0.3 µm AP-D suspension, Struers) and simply left to dry at room temperature. The 30 µL were added in two steps of 15 µL with drying in between the steps. All the electrochemical experiments were conducted using a computer-controlled potentiostat (Eci-100, NordicElectrochemistry) at room temperature (ca. 22 °C). The electrolytes (ca. 20 mL in a Pyrex container) were not purged with N_2 or any other gases and no rotation was performed. Each sample was characterized by the following sequence of steps with rinsing with water in-between the different steps:

A) 3 scans at 50 mV s^{-1} in 1 M KOH (without ethanol) between -0.50 and 0.70 $V_{Hg/HgO}$
B) 3 scans at 50 mV s^{-1} in 1 M KOH + 1 M ethanol between -0.50 and 0.70 $V_{Hg/HgO}$
C) 3 scans at 50 mV s^{-1} in 0.5 M H_2SO_4 between 0.00 and 1.50 V_{SCE}

2. UV-vis Au, Ag and Au-Ag

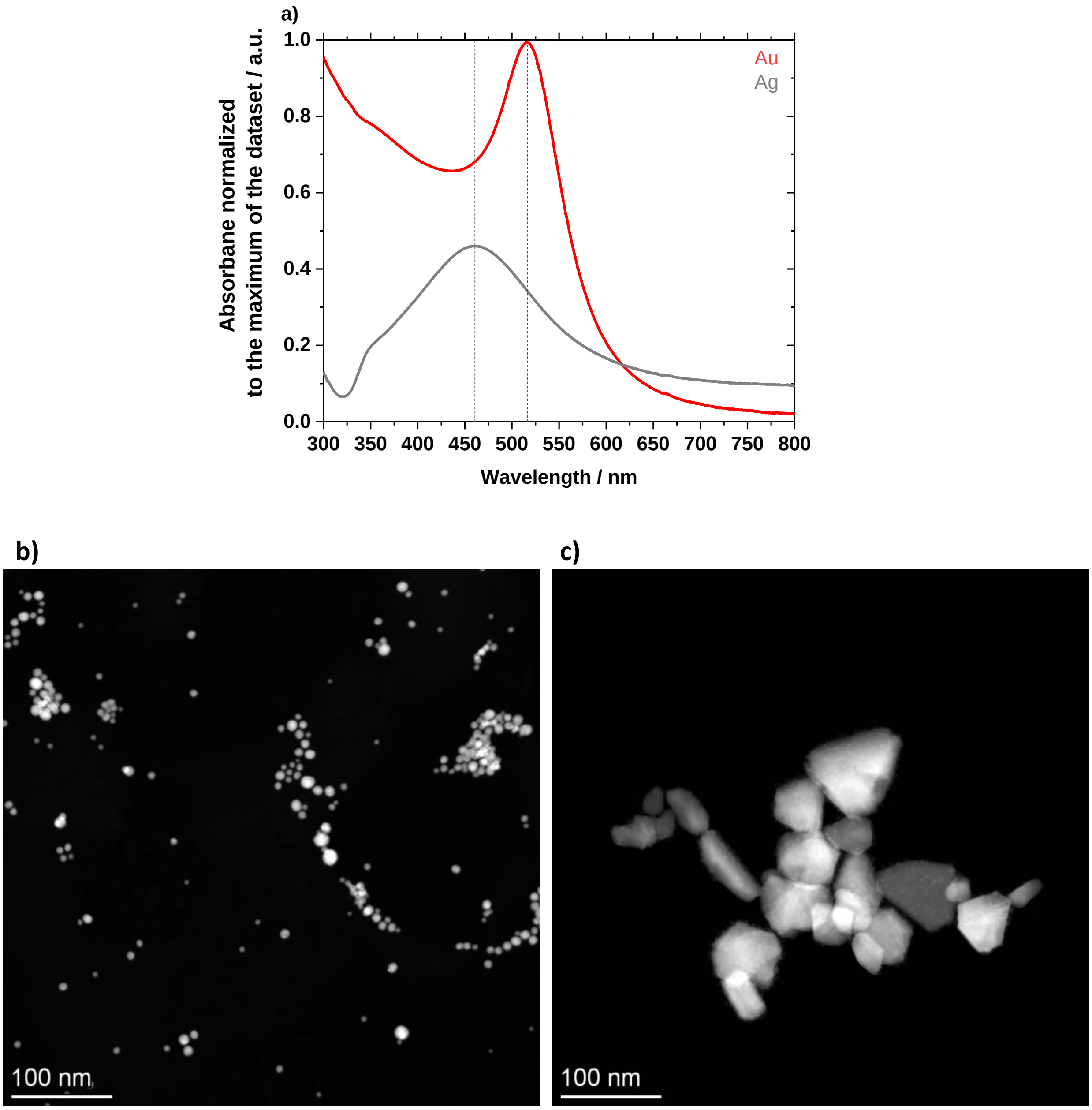

Figure S6. (a) Illustrative UV-vis spectra of Au and Ag NPs obtained by an ethanol-mediated synthesis in alkaline water-ethanol mixtures, as indicated. The vertical dotted lines are a guide for the eyes to identify the A_{spr}. (b-c) STEM micrographs of the corresponding (b) Au and (c) Ag NPs. 30 v.% ethanol and 2 mM NaOH were used for 0.5 mM $HAuCl_4$ and 0.5 mM $AgNO_3$. The spectra reported un (a) are the same as in **Figure 1** in the manuscript and reported here for clarity.

3. Composition control

Depending on the synthesis conditions used, more or less Au relative to Ag is observed and more or less of the metal is observed relative the nominal atomic ratio. An overview of the experimental composition retrieved from the analysis of individual NPs as a function of the expected composition is given in **Figure S7**. In the next sections are also indicated the experimental compositions as a function of NP size.

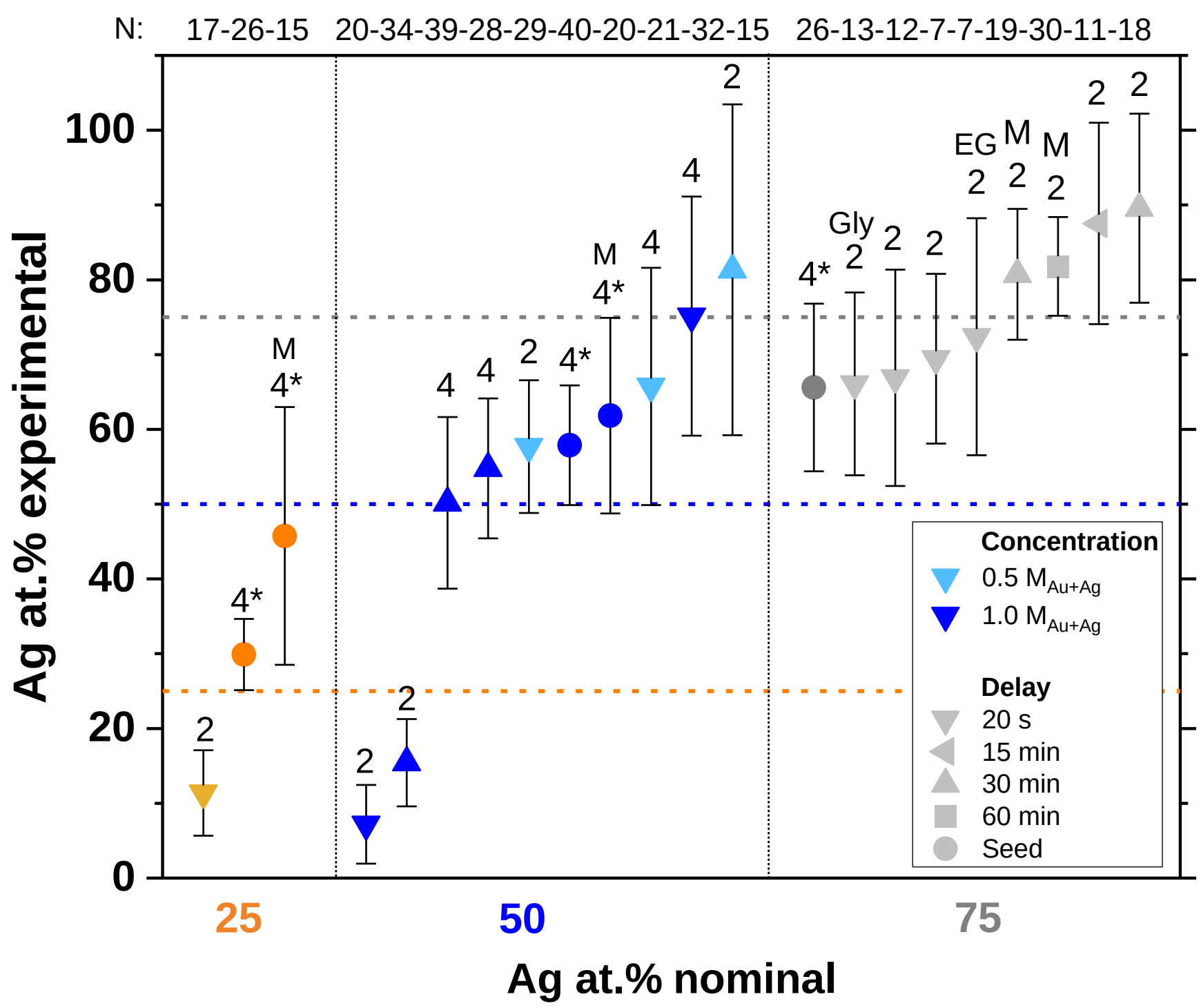

Figure S7. Experimental composition as a function of nominal composition for samples prepared under various conditions of metal concentration (light datapoints: 0.5 mM$_{Au+Ag}$, darker datapoints: 1.0 mM$_{Au+Ag}$), NaOH concentration (indicated on top of the error bars, a * means that the total amount was added in two steps: half at the beginning of the synthesis and the other half before or after adding $AgNO_3$). The total number of NPs counted is given at the top. The time delay for addition of the precursors is coded by the different shape of the datapoints, as indicated. The round datapoint correspond to a case where pre-made Au NPs were used. The alcohol used for the synthesis is given by a letter above the error bars (nothing: ethanol; M: methanol; EG: ethylene glycol; G: Glycerol).

Overall the nominal composition agrees rather well with the experimental composition with deviation as it can be expected.[5] As discussed below, it can be seen, especially for a 50:50 Au:Ag ratio, that using a NaOH/(Au+Ag) molar ratio of 4 lead to a better match between experimental and nominal composition (comparison of light blue and dark blue datapoints: those with a number 2 for light blue and 4 for dark blue lead to a better match between measured and expected composition).

4. Notes on parametric study

The multiplicity of parameters that can influence the outcome of the synthesis is very large and include, among others: total concentration of precursors, relative ratios of the precursors, nature of the precursor, nature of alcohols, amount of alcohol, light, concentration of base, time delay between the addition of the precursor, order of addition of the precursor, sequential addition of the base, temperature, stirring or not, use of gases, different containers used, etc.[1-3,5,10,13-15]

In this first report, only an overview of some parameters is reported to illustrate the potential of the synthesis approach without aiming to provide (yet) a clear picture of the influence of each and every one of the many potential experimental knobs that can be tuned.

It is however important to keep in mind:

1) that while Au NPs ca. 10-20 nm are easily obtained in a surfactant-free room temperature ethanol-mediated synthesis,[2,5] Ag NPs are more challenging to obtain and typically show undefined shapes and a rather large size around 30 nm or more,[16] see for instance **Figure S6**.

2) that obtaining Au-Ag or Au@Ag NPs depends on the time delay between the addition of Au and Ag precursor. A mixture of $HAuCl_4$ and $AgNO_3$ lead to a AgCl precipitate that is not easily used directly as precursor.

3) that attempts to use $AgNO_3$ and then $HAuCl_4$ precursor did not lead to well-defined materials (no regular shape) probably due to galvanic displacement, as illustrated in **Figure S8**, and this strategy was not pursued further at this stage.

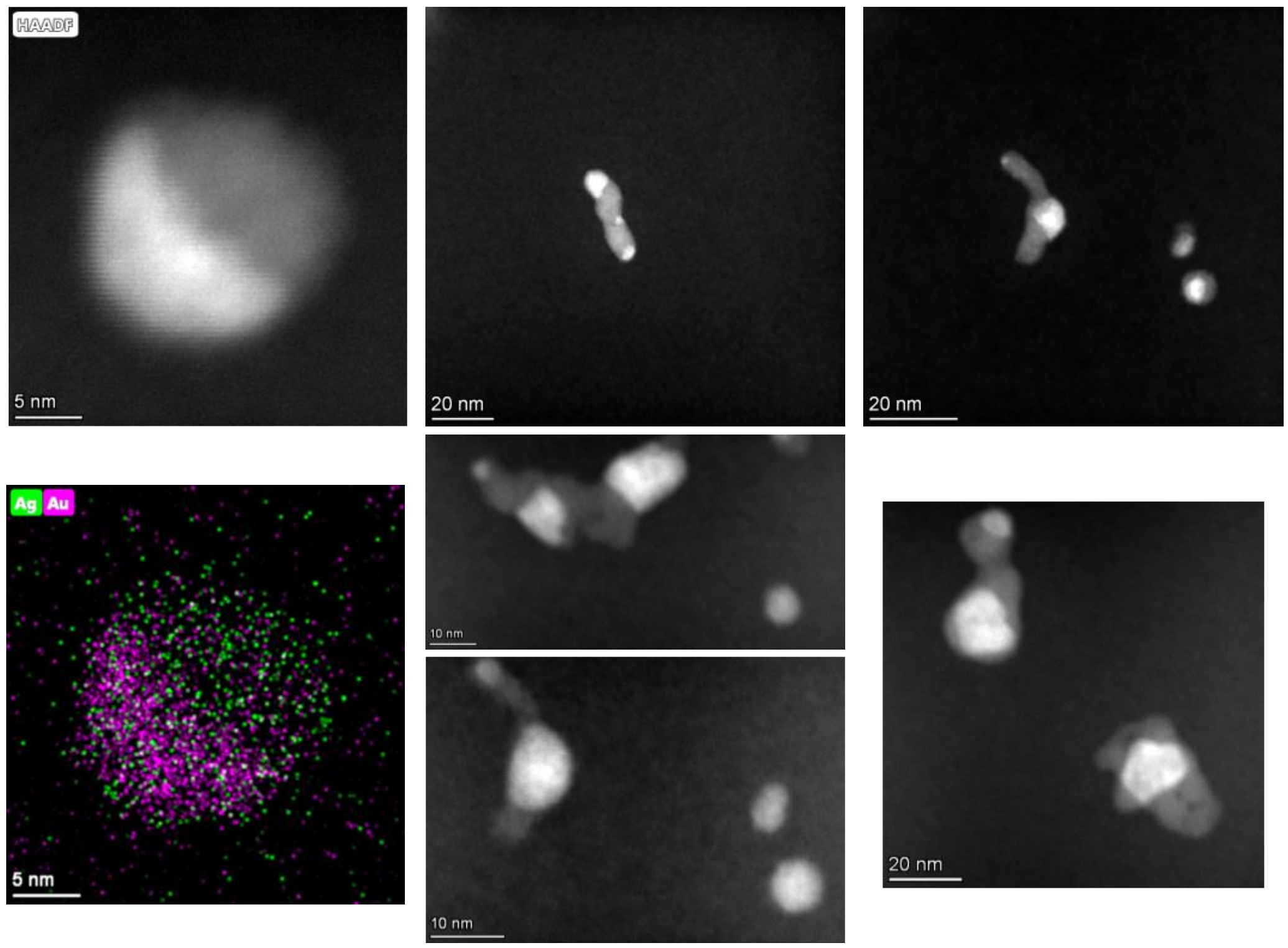

Figure S8. Illustrative STEM-EDS micrographs illustrating the most challenging control over the properties of Ag-Au NPs when $AgNO_3$ is added first. In this case the synthesis was performed with 25 v.% glycerol, 0.5 mM_{Au+Ag} and a Au:Ag molar ratio of 25:75 for 2 mM of NaOH. The color map used in this figure differs from the color code used in the other figures to stress that the order of addition of the precursor was different. The data set on the left confirms that the brighter features in the dark field image in are Au-rich.

5. Delayed addition of $HAuCl_4$ and $AgNO_3$

5.1. Short delay

In this entire **section 5.1**, the time delay between the addition of $HAuCl_4$ and $AgNO_3$ is 20 seconds, as detailed in **section 1.2** and **Figure S1**.

Table S1. Overview of the parameter space screened with a delayed addition of the precursors of 20 seconds between the addition of $HAuCl_4$ and $AgNO_3$.

<table>
<tr><th>Alcohol</th><th>Alcohol
v.%</th><th>NaOH
mM</th><th>Precursor
mM_{Au+Ag}</th><th>$HAuCl_4$:$AgNO_3$
molar ratio</th></tr>
<tr><td>Ethanol</td><td>5, 10, 20, 30, 40, 50, 75, 95</td><td>2, 3, 4</td><td>0.5, 0.67, 1, 2</td><td rowspan="2">100:00
75:25
50:50
25:75</td></tr>
<tr><td>Methanol</td><td rowspan="3">30</td><td rowspan="3">2</td><td rowspan="3">0.5</td></tr>
<tr><td>Ethylene glycol</td><td>25:75</td></tr>
<tr><td>Glycerol</td><td>25:75</td></tr>
</table>

5.1.1. Influence of the alcohol

A general challenge is to obtain Ag-rich samples using surfactant-free alcohol-mediated syntheses, e.g. no clear plasmon is obtained when Ag only materials are prepared as presented in **Figure S6**. It is also shown in **Figure S13** that rather large materials with poorly defined shapes are obtained. The exception would be when ethylene glycol is used, detailed elsewhere.[16] A comparison of the effect of the alcohol is presented in **Figure S9**. Ethanol and glycerol are the alcohols leading to the NPs with the clearest spr features, probably due to the more regular shape of the NPs obtained, see **Figure S10**. It is worth noting that it is relatively simple to observe the formation of surfactant-free Au@Ag NPs when glycerol is used, see **Figure S11**. In light of the discussion provided below this is attributed to the very fast formation of Au NPs when glycerol is preferred.[14]

Glycerol is a rather viscous solvent which complicate the processing of the colloids.[13] Despite the possible challenges, a stronger focus is here given to the mono-alcohol mediated syntheses due to the lower viscosity and lower boiling points of the methanol and ethanol. Particularly promising is a clear spr observed for the Ag-rich materials when ethanol is used as source or reducing agent.

Ethanol-mediated syntheses are promising to develop surfactant-free Au-Ag NPs.

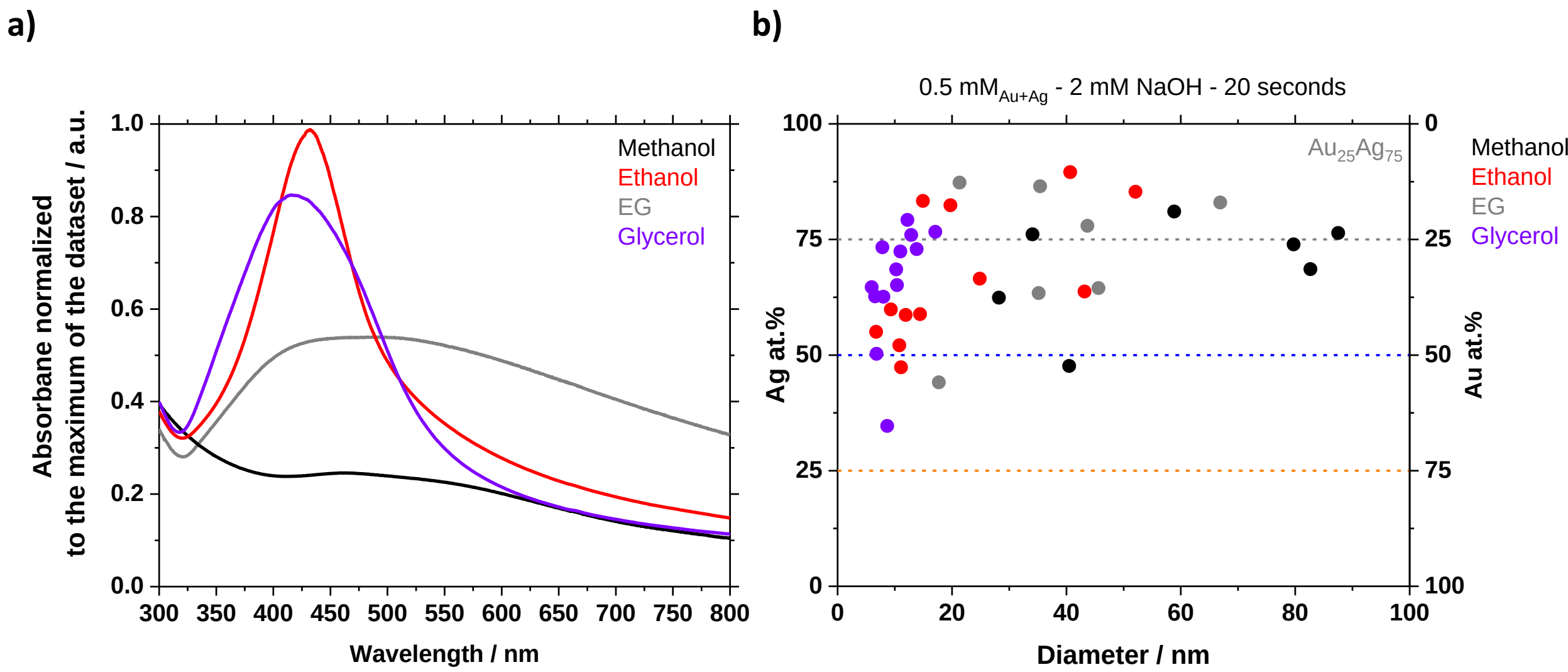

Figure S9. (a) Illustrative UV-vis spectra for NPs obtained for syntheses performed with different alcohols. In all cases 0.5 mM$_{Au+Ag}$, 2 mM NaOH, 30 v.% alcohol and a nominal Au:Ag atomic ratio of 25:75 were used with 20 seconds delay between the addition of $HAuCl_4$ and $AgNO_3$. (b) Size-composition relationship for the NPs.

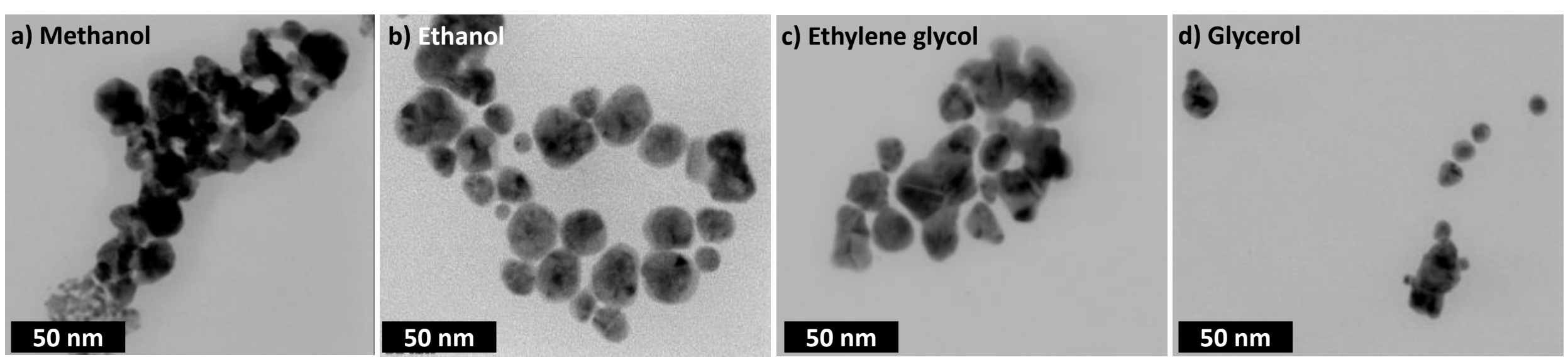

Figure S10. (a) Illustrative STEM bright field micrographs of the NPs obtained for syntheses performed with different alcohols. In all cases 0.5 mM$_{Au+Ag}$, 2 mM NaOH, 30 v.% alcohol and a nominal Au:Ag atomic ratio of 25:75 were used.

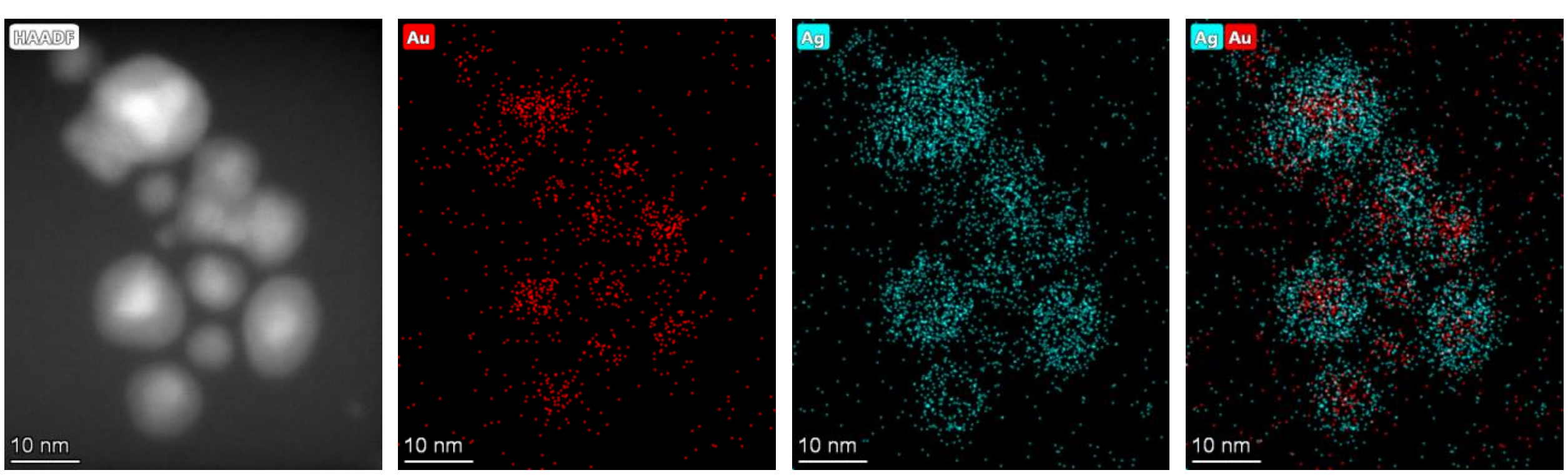

Figure S11. Illustrative STEM micrographs and EDS map for Au an Ag, as indicated, for NPs obtained for syntheses performed with glycerol. 0.5 mM$_{Au+Ag}$, 2 mM NaOH, 30 v.% glycerol and a nominal Au:Ag atomic ratio of 25:75 were used.

5.1.2. Influence of the Au:Ag ratio

As the Ag content increases the λ_{spr} value decreases and the NPs tend to become larger, see **Figure S12a**. The nominal Au:Ag molar ratio controls the final relative amount of the metals, see **Figure S12b** and **Table S2**. However, the NPs tend to not be spherical with a short delayed addition of the precursors, see **Figure S13**.

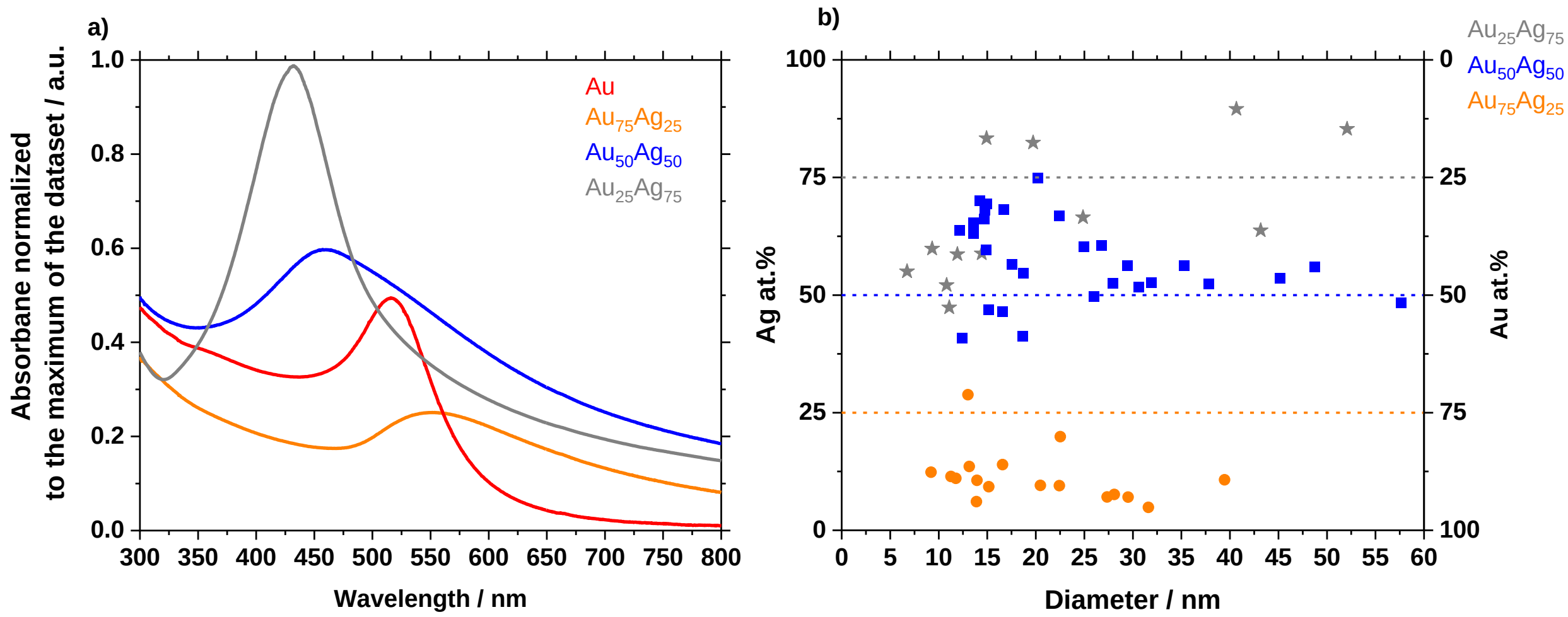

Figure S12. (a) Illustrative UV-vis and (b) related composition as a function of size for NPs prepared using different nominal Au:Ag atomic ratios, as indicated. The synthesis was obtained using 30 v.% ethanol, 0.5 mM_{Au+Ag} and a NaOH concentration of 2 mM..

Table S2. Overview of the properties of the NPs obtained using 30 v.% ethanol, 0.5 mM_{Au+Ag} and a NaOH concentration of 2 mM.

**$HAuCl_4$:$AgNO_3$** molar ratio	**Au:Ag** molar ratio EDS based on individual NPs (deviation)	**Diameter** nm	**N**	**λ_{spr}** nm
100:0	-	7.5 ± 2.4	276	516
75:25	89:11 (6)	20 ± 9	17	552
50:50	42:58 (9)	22 ± 13	131	459
25:75	33:67 (14)	26 ± 14	24	431
0:100	-	30+	-	463

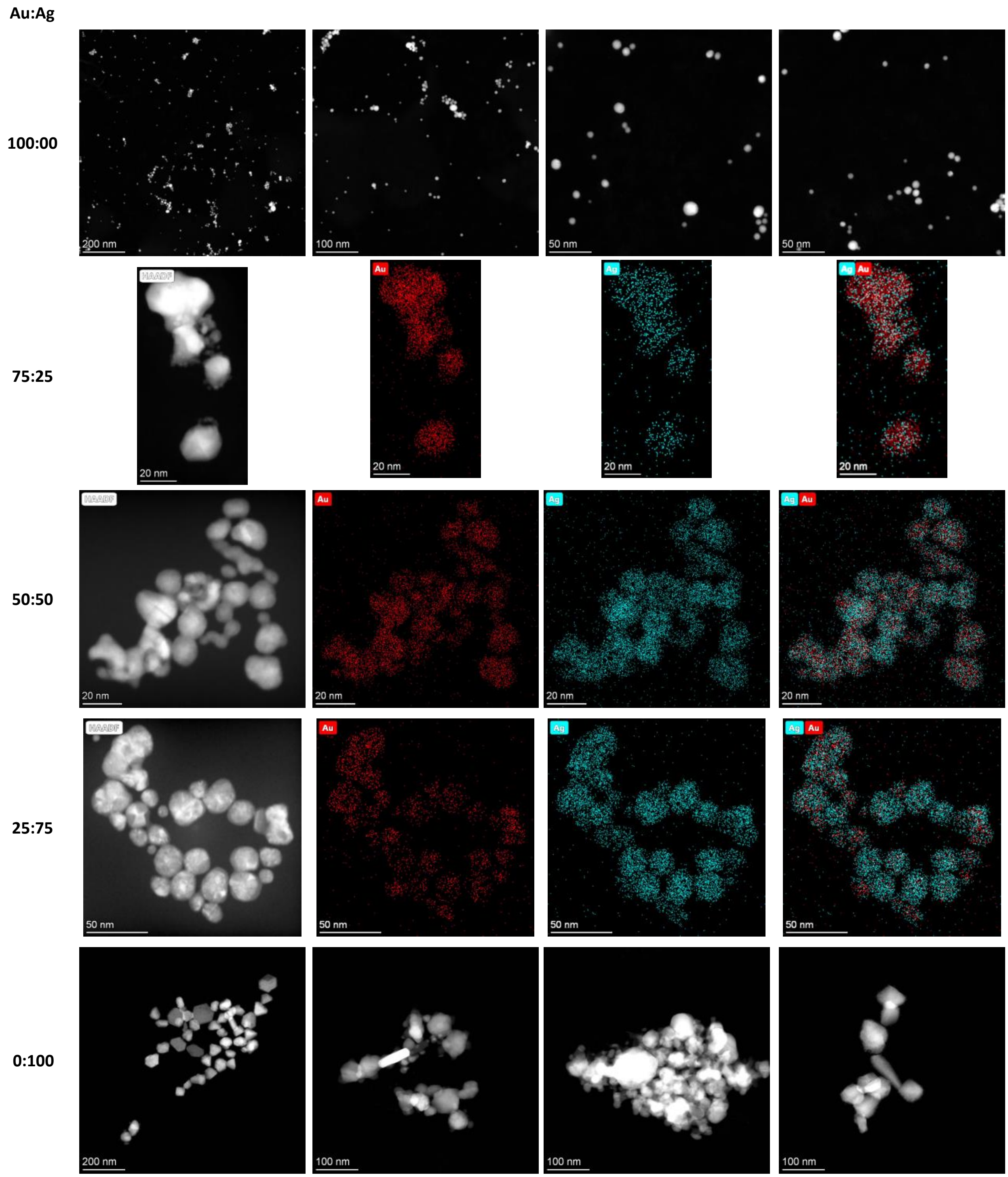

Figure S13. Illustrative STEM-EDS data for samples obtained using 0.5 mM_{Au+Ag}, 2 mM NaOH and and 30 v.% ethanol. The $HAuCl_4$:$AgNO_3$ molar ratios was tuned between 75:25, 50:50, 27:75, 0:100 as indicated. 20 seconds were left between the addition of $HAuCl_4$ and $AgNO_3$, when relevant.

5.1.3. Influence of the ethanol content

In agreement with previous report, for Au NPs only (100% Au) a ratio 20-30 v.% ethanol is ideal to obtain small size NPs with a well-defined spr,[2,5] see **Figure S14a**. The synthesis of Ag NPs by the same method is challenging regardless of the ethanol content,[16] see **Figure S13**. An ethanol content of 30 v.% is here also ideal to obtain the UV-vis spectra with the more pronounced spr features in particular at higher Ag contents.

The lower stability and absence of spr features for high ethanol content are attributed to an excess of reducing agent and the poorly defined feature at 10 v.% are attributed to a lack of reducing agent.

30 v.% ethanol was considered an optimal choice for the synthesis of Au-Ag NPs.

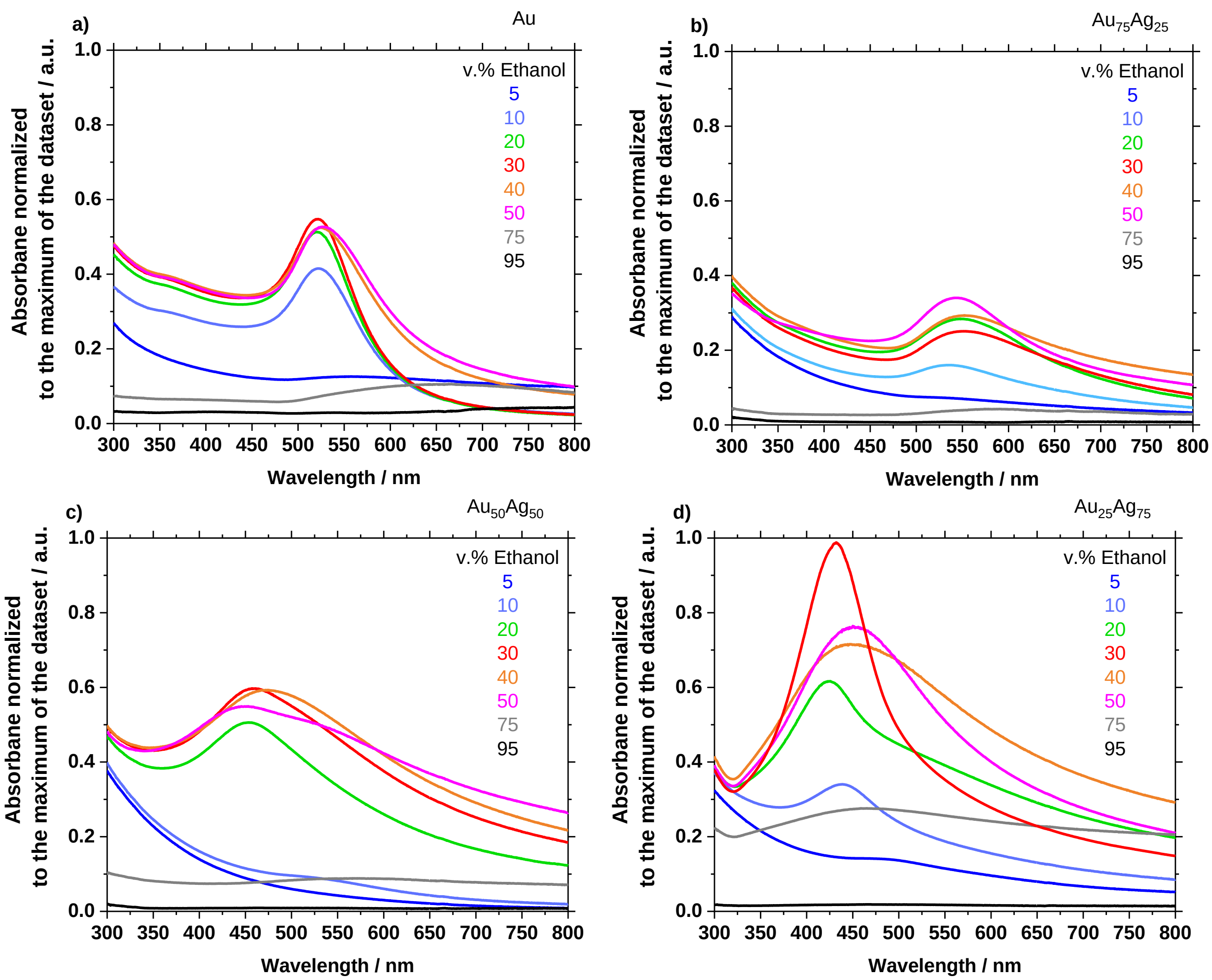

Figure S14. Illustrative UV-vis spectra of samples obtained using 0.5 mM_{Au+Ag} and 2 mM NaOH. The $HAuCl_4$:$AgNO_3$ molar ratios was tuned between (a) 100:0, (b) 75:25, (c) 50:50 and (d) 27:75 varying the v.% of ethanol between 5, 10, 20, 30 40, 50, 75 and 95 v.%.

5.1.4. Influence of the base

The best defined spr features (well defined unique spr peak) were otbained using 2 mM NaOH in most cases and especially at higher Ag contents, see **Figure S15**.

A NaOH concentration of 2 mM was preferred to obtain clearer spr features and to minimize the amount of base used to develop more cost-efficient and more sustainable syntheses

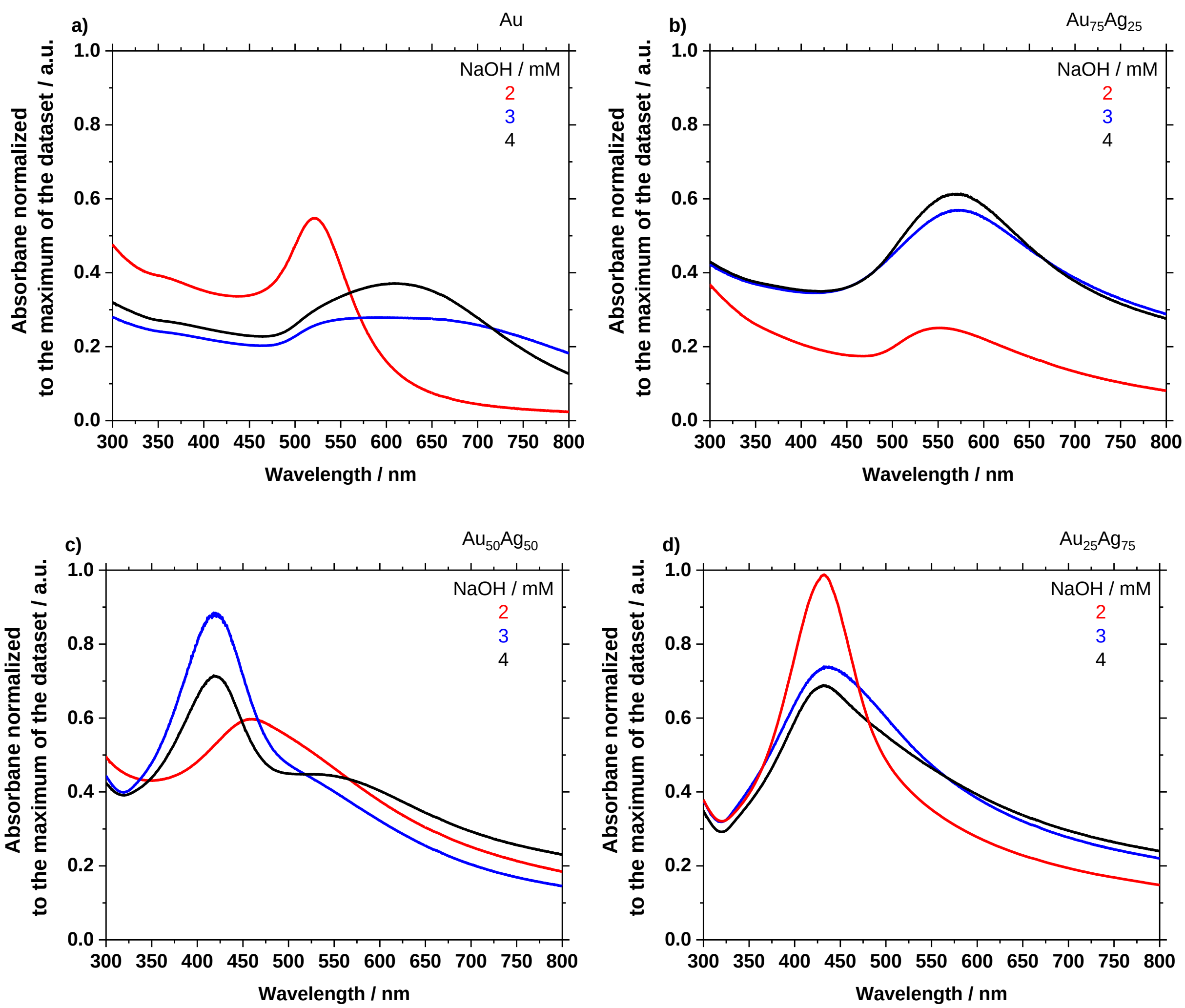

Figure S15. Illustrative UV-vis data for samples obtained using 0.5 mM_{Au+Ag} and 30 v.% ethanol. The $HAuCl_4$:$AgNO_3$ molar ratios was tuned between (a) 100:0, (b) 75:25 (c) 50:50 and (d) 27:75 for a concentration in NaOH of 2, 3 or 4 mM, as indicated.

5.2. Long delay

One of the main challenge of the sequential addition of the previous **section 5.1**, is that the time between the addition of the two precursors can be difficult to control. In this section, the impact of the time delay between the two precursors addition is investigated in more details.

In this entire **section 5.2**, the time delay between the addition of $HAuCl_4$ and $AgNO_3$ is several minutes, as detailed in **section 1.2** and **Figure S2**. Using 30 minutes or 60 minutes delay addition was not showing strong differences and a focus below is on samples obtained using 30 minutes.

Table S3. Overview of the parameter space screened with a delayed addition of the precursors of several minutes between the addition of $HAuCl_4$ and $AgNO_3$.

**Alcohol**	**NaOH*** mM	**Precursor** mM_{Au+Ag}	**$HAuCl_4$:$AgNO_3$** molar ratio	**Time delay between $HAuCl_4$ and $AgNO_3$ addition**** min
Ethanol	2, 4	0.5, 0.67, 1, 2	75:25<br>50:50<br>25:75	15, 30, 60
Methanol	2	0.5		30, 60

* The NaOH was preferentially added in 2 steps with an equal volume each time as detailed in the text, half before adding $HAuCl_4$ and half after adding $AgNO_3$.
** Note that for a 30-60 min time of reaction it is expected that the synthesis of Au NP is still proceeding.[2,14]

5.2.1. Sequential addition of the precursors

The delayed addition of $AgNO_3$ has a strong influence on the UV-vis spectra and morphology of the NPs. The results below in **Figure S16** and **Figure S17** are to be compared with those obtained with a 20 seconds delay between the addition of $HAuCl_4$ and $AgNO_3$ reported in **Figure S12** and **Figure S13**. The results from **Figure S12** are reported in **Figure S16** below to facilitate the comparison.

Delaying the addition of the precursor tend to lead to UV-vis spectra with features closer to Au NPs in the sense that the spectra with Au:Ag molar ratios of 75:25 and 50:50 show not only a maximum shifted towards lower wavelengths but also a feature closer to 500 nm, especially clear for the 50:50 samples. This suggests that the particles are more silver rich, smaller in size and/or with a different structure.

A closer look at the STEM-EDS data reported in **Figure S18** indicates slightly smaller NPs when the NPs are obtained with a 20 seconds delay, and has the time of addition delay increases the NPs tend to present a core made of Au NPs that tend to be larger but is not spherical. In addition, there is a trend to see NPs of various compositions depending on size as illustrated in **Figure S16b** and **Figure S19**, where the smallest NPs tend to be more Ag-rich whereas the larger ones tend to be more Au-rich.

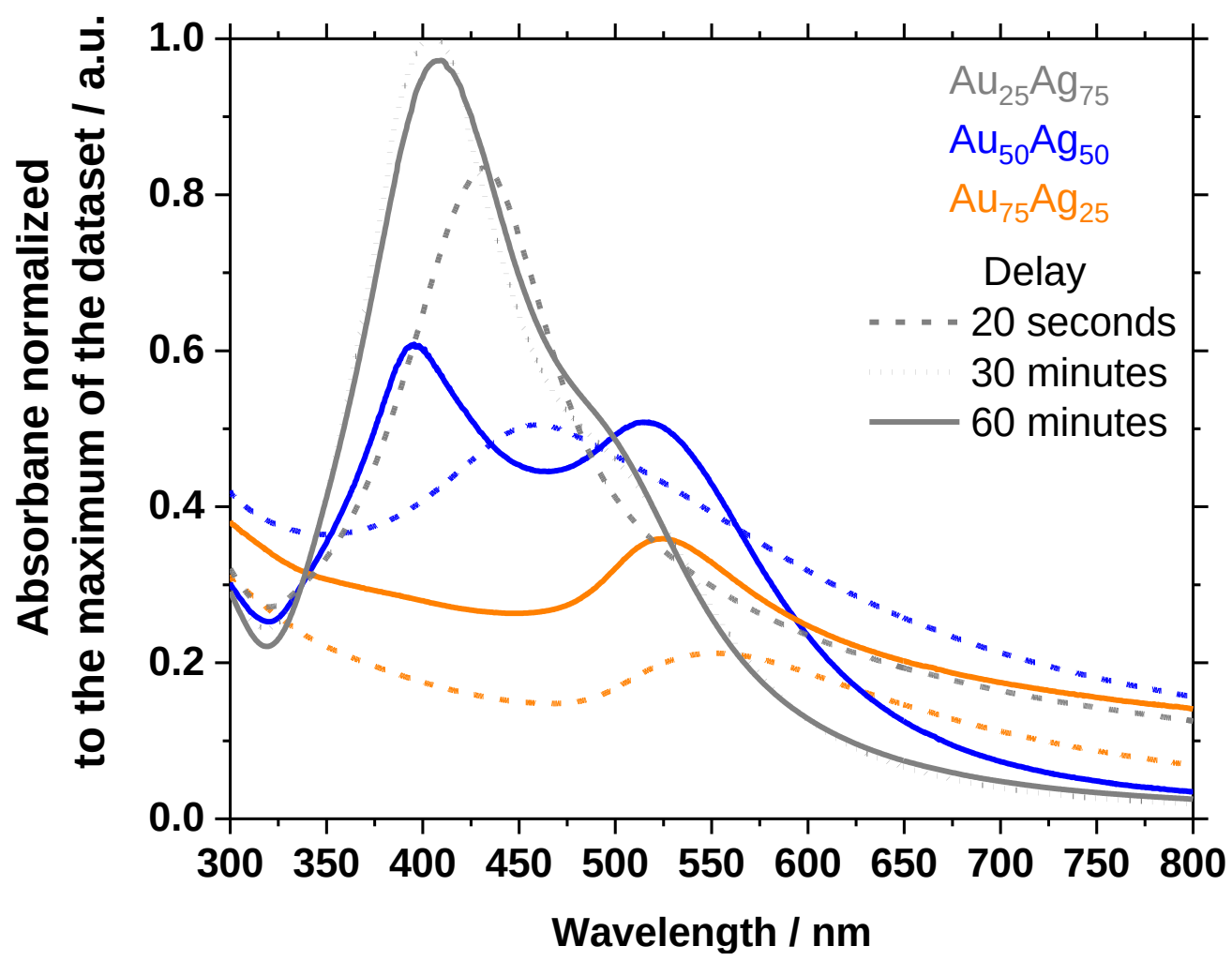

Figure S16. Illustrative UV-vis spectra for samples prepared with 0.5 mM_{Au+Ag} and a Au:Ag ratio of 75:25, 50:50 or 75:25, as indicated, obtained using 30 v.% ethanol and 2 mM NaOH. The time between the addition of $HAuCl_4$ and $AgNO_3$ was 20 seconds or 30 minutes, as indicated. The dataset corresponding to 20 seconds is the same as in **Figure S12a** and reported to facilitate the comparison.

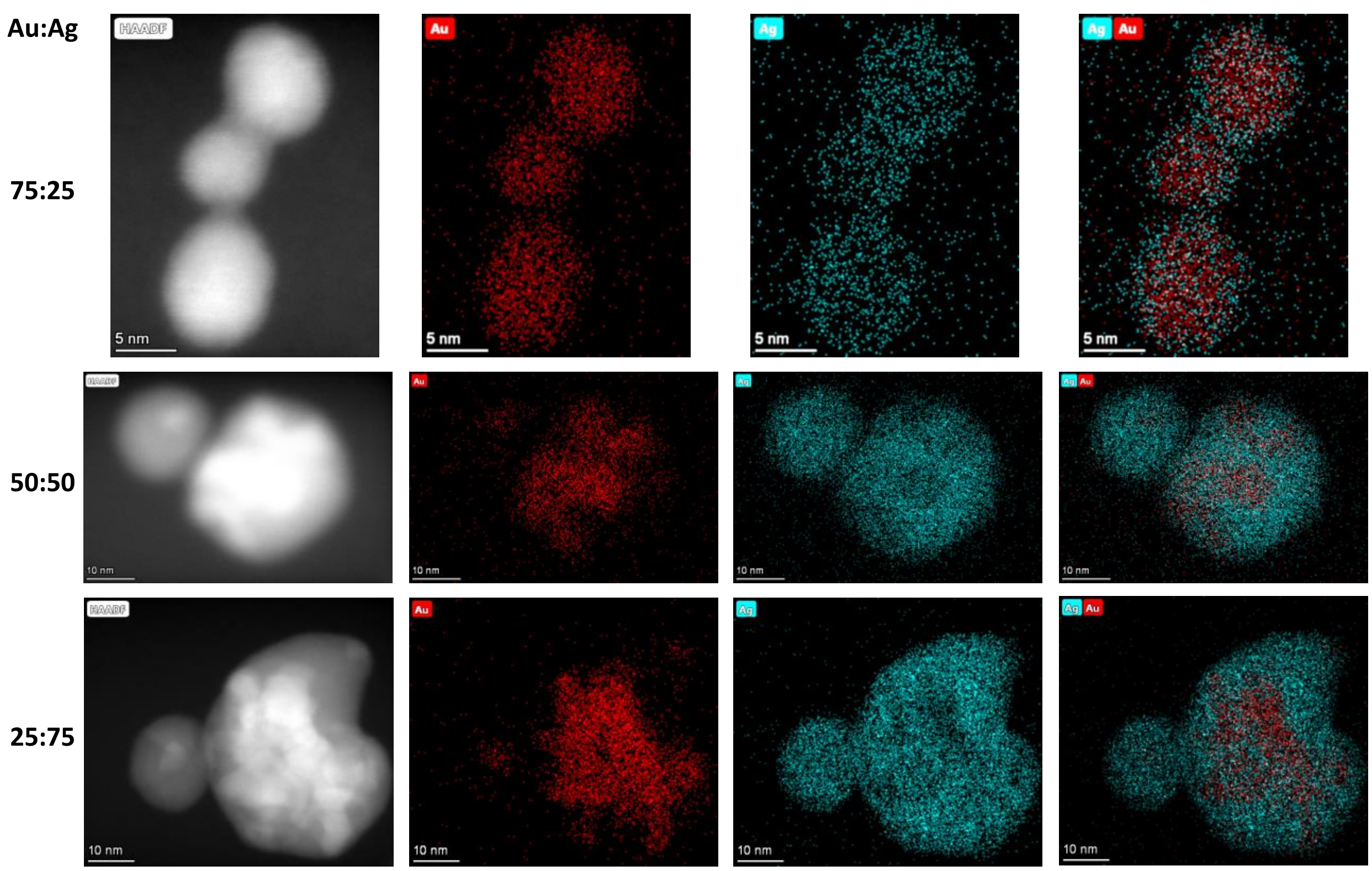

Figure S17. Illustrative STEM-EDS data for samples obtained using 0.5 mM_{Au+Ag}, 2 mM NaOH and 30 v.% ethanol. The $HAuCl_4$:$AgNO_3$ molar ratios was tuned between 75:25, 50:50 and 27:75, as indicated. 30 minutes were left between the addition of $HAuCl_4$ and $AgNO_3$.

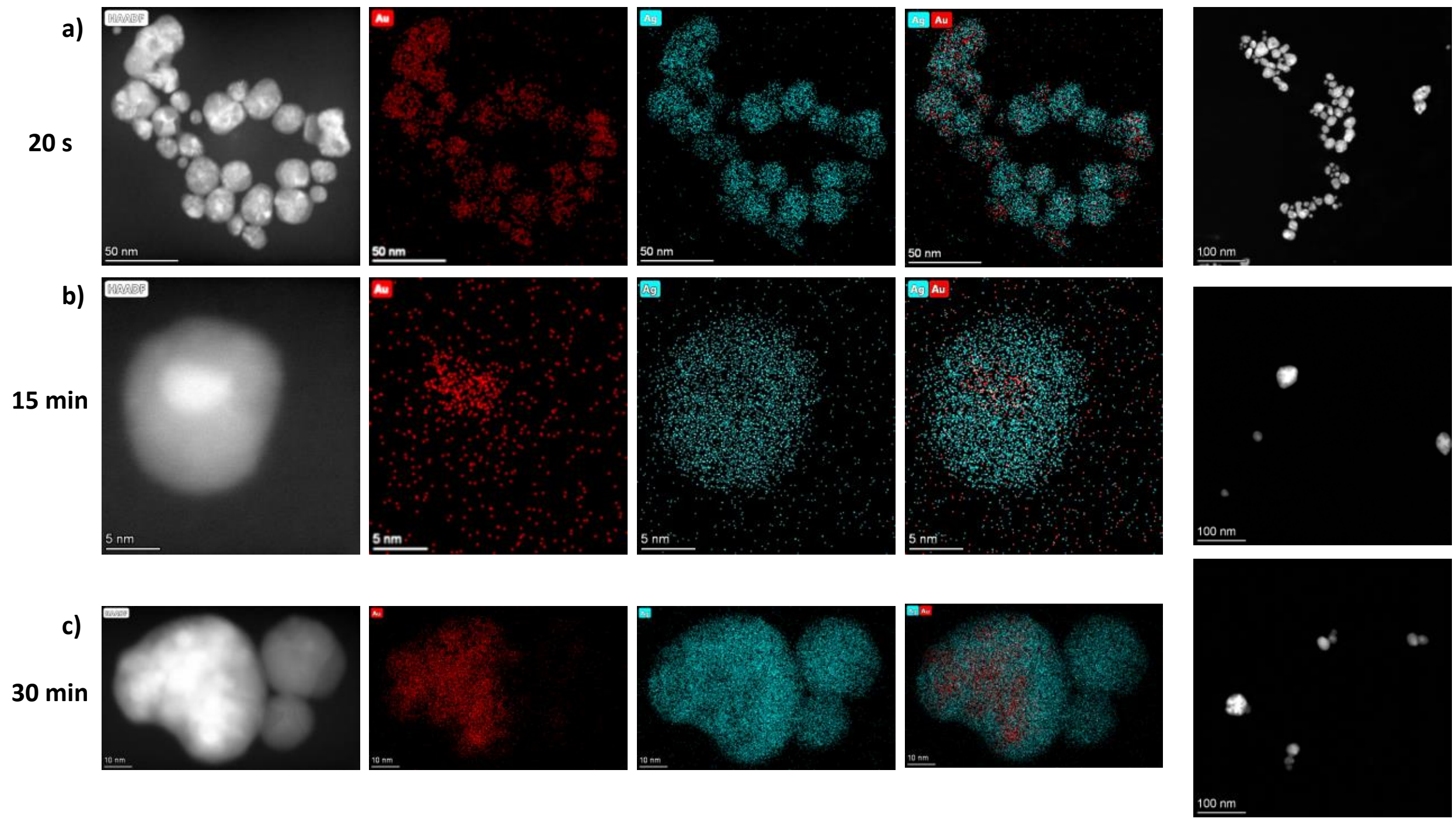

Figure S18. Illustrative STEM-EDS micrographs for samples prepared with 0.5 mM_{Au+Ag} and a Au:Ag ratio of 25:75 obtained using 30 v.% ethanol and 2 mM NaOH. The time between the addition of $HAuCl_4$ and $AgNO_3$ was (a) 20 s, (b) 15 minutes or (c) 30 minutes.

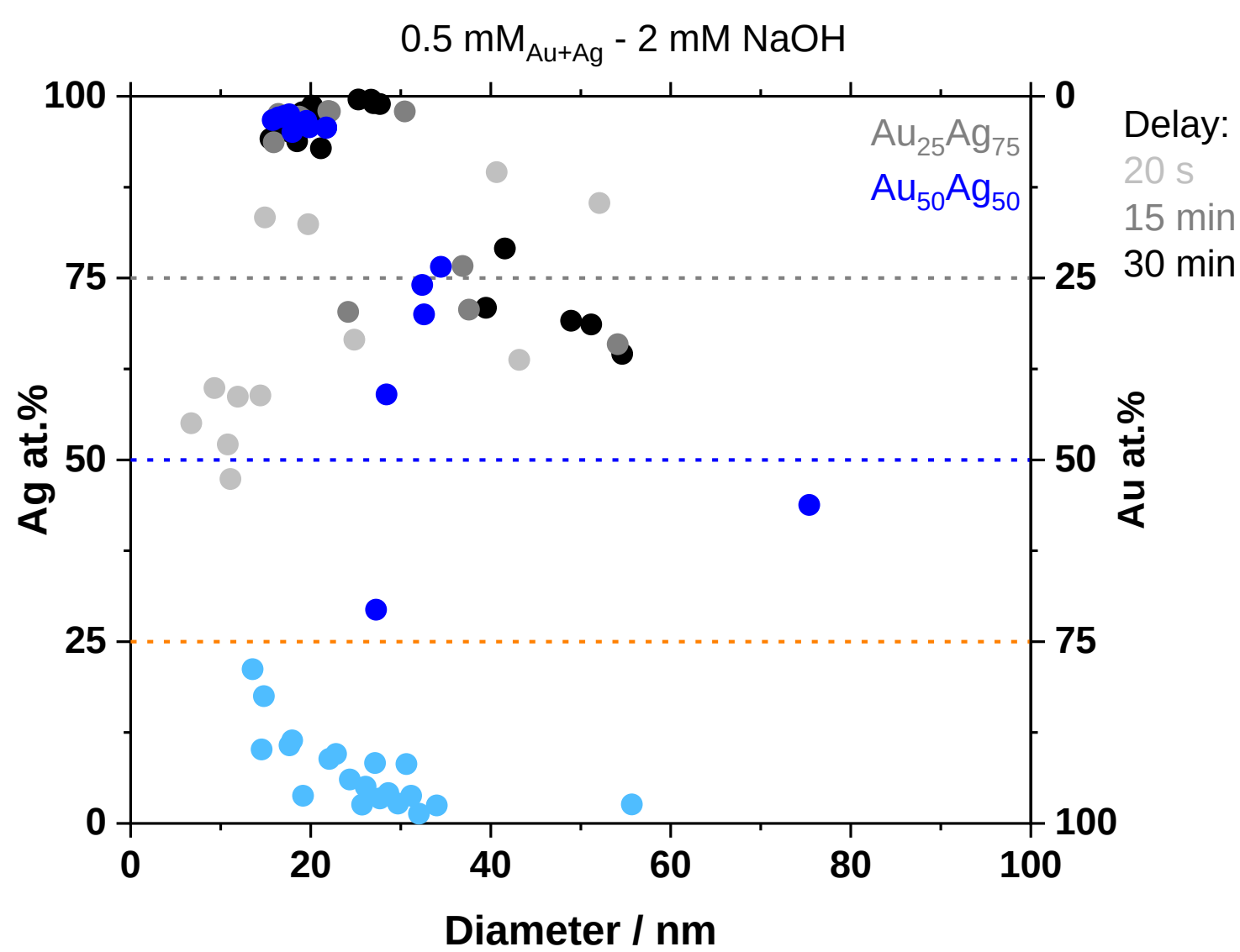

Figure S19. Size-composition relationship for the NPs prepared using 0.5 mM_{Au+Ag} and a Au:Ag ratio of 25:75 or 50:50, as indicated, obtained using 30 v.% ethanol and 2 mM NaOH. The time between the addition of $HAuCl_4$ and $AgNO_3$ was 20 s, 15 minutes or 30 minutes, as indicated. The dataset corresponding to 20 seconds is the same as in **Figure S12b** and reported to facilitate the comparison.

5.2.2. Scale up in concentration and sequential addition of the base

We then assessed if the synthesis could be scaled up in concentration. We focused on considering 0.5 mM_{Au}. In the case of Au:Ag molar ratios of 50:50 that leads to 1.0 mM_{Au+Ag}, considering that a NaOH/Au molar ratio of 4 is ideal to prepare Au NPs, 4 mM NaOH were first used. As illustrated in **Figure S20**, with a 20 seconds delay addition, Au-Ag NPs are obtained just like in the case where 0.5 mM_{Au+Ag} was used.

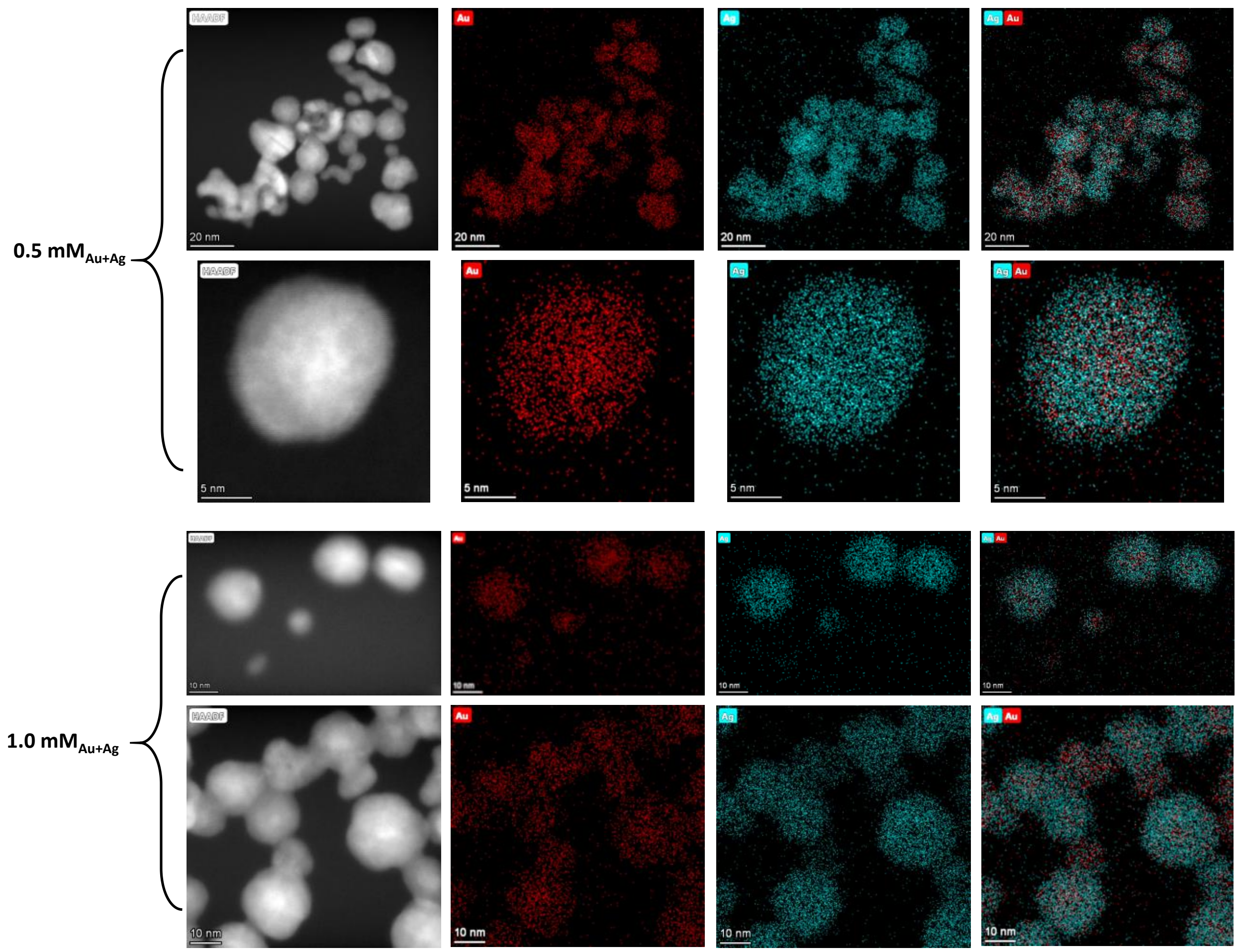

Figure S20. STEM-EDS micrographs of NPs obtained using 0.5 or 1.0 mM_{Au+Ag}, as indicated. 30 v.% ethanol was used in all cases and the $HAuCl_4$:$AgNO_3$ molar ratios was 50:50 for a NaOH/Au molar ratio of 4 and NaOH was added from the beginning of the reaction. The time delay between the addition of $HAuCl_4$ and $AgNO_3$ was 20 seconds.

However, if only 2 mM NaOH was used, NP with a Ag-poor composition compared to what was expected were observed, see **Figure S21**. This results stress the need to use enough base to perform the reduction via the alkoxide formed from the alcohol.[5]

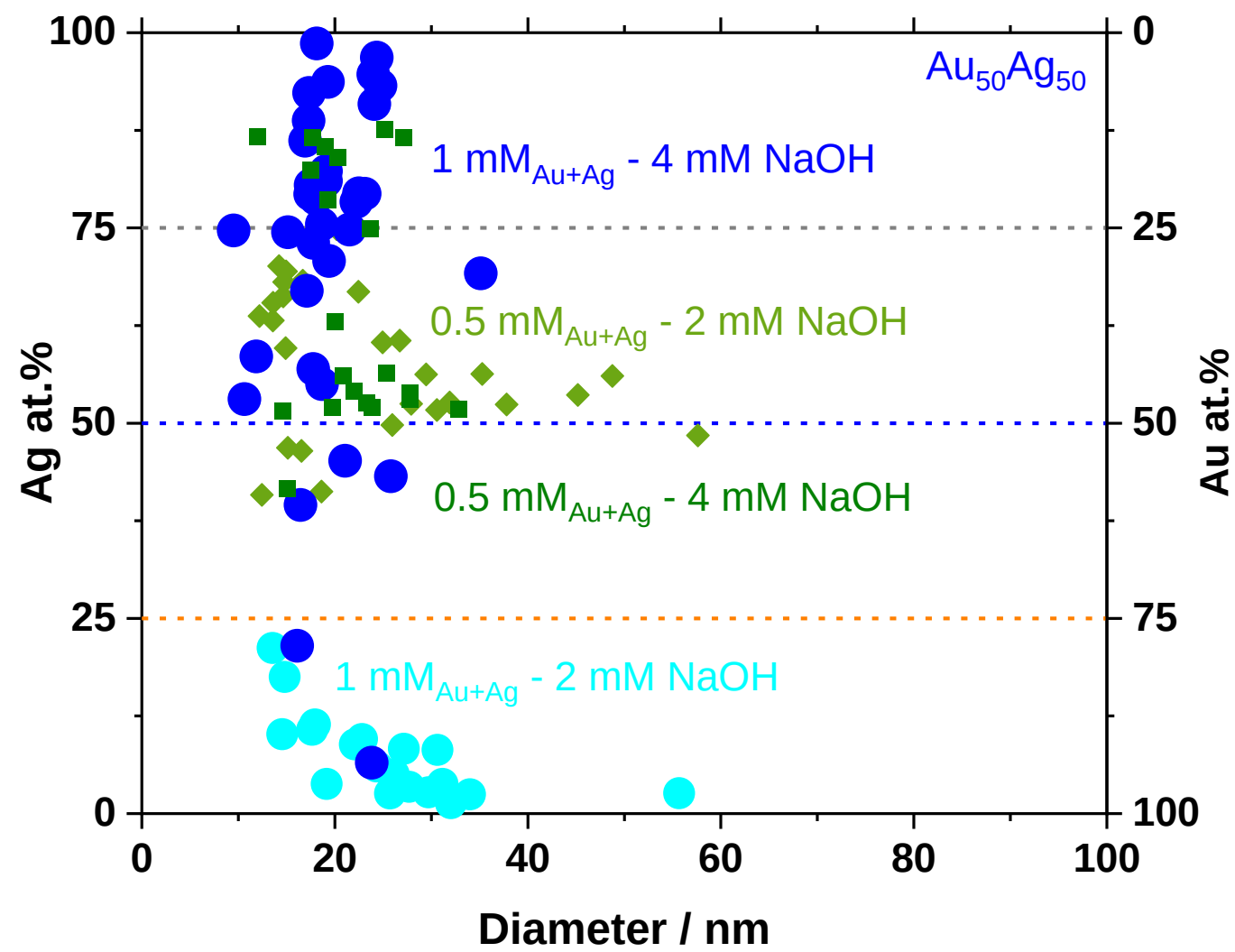

Figure S21. Size-composition relationship for the NPs prepared using 0.5 or 1.0 mM_{Au+Ag}, as indicated. 30 v.% ethanol was used in all cases and the $HAuCl_4$:$AgNO_3$ molar ratios was 50:50 for a NaOH concentration of 2 or 4 mM, as indicated where NaOH was added from the beginning of the reaction. The time delay between the addition of $HAuCl_4$ and $AgNO_3$ was 20 seconds.

When a 30 minutes addition was performed, Au@Ag NPs were obtained as detailed in **Figure S23** however the shell is rather small. This is in agreement with the UV-vis spectra in **Figure S22**, with features close to those of Au NPs and the high absorbance observed before and after the plasmon resonance suggests unreacted precursor. This can be explained by the fact that most of the alkoxide required for the reduction of the Au NPs has been consumed at this stage.[5] Therefore adding NaOH might be required for further reduction by 'activation' of the remaining ethanol present in excess.

It is important to note for the following that a concentration of 4 mM NaOH is not optimal for synthesizing Au-only NPs from 0.5 mM_{Au}, see **Figure S15**. Here, for the first 30 minutes, only $HAuCl_4$ is present and to optimize the growth of Au NPs half of the base is added before the addition of $HAuCl_4$ and half of the base after the addition of $AgNO_3$. In other words an extra equivalent of 2 mM of NaOH is added after 30 minutes, leading to a total concentration of 4 mM.

The resulting UV-vis spectra then show features more related to Ag plasmonic properties with more pronounced peaks at lower wavelengths. STEM-EDS in **Figure S23** confirms that adding more base the second time leads to larger shell suggesting a more efficient reduction of the Ag. There seems to be no strong influence if the base is added before or after $AgNO_3$, **Figure S23b-c**.

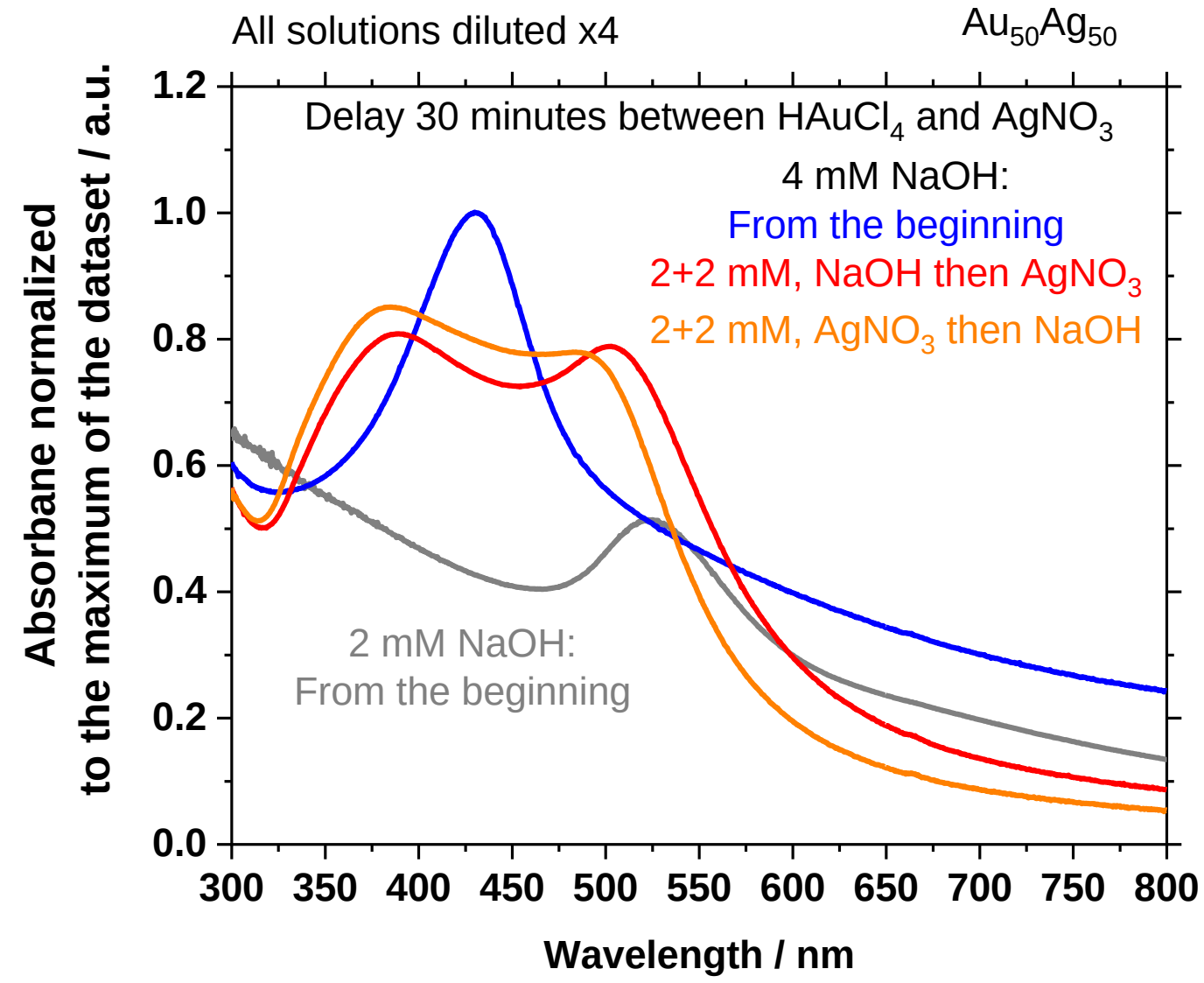

Figure S22. (a) UV-vis spectra for syntheses performed using 1.0 mM_{Au+Ag} and 30 v.% ethanol. The $HAuCl_4$:$AgNO_3$ molar ratios was 50:50, as indicated, for a concentration in NaOH of 2 mM added only once or for 4 mM added in two steps of 2 mM. The sample were all diluted by 4 for measurement.

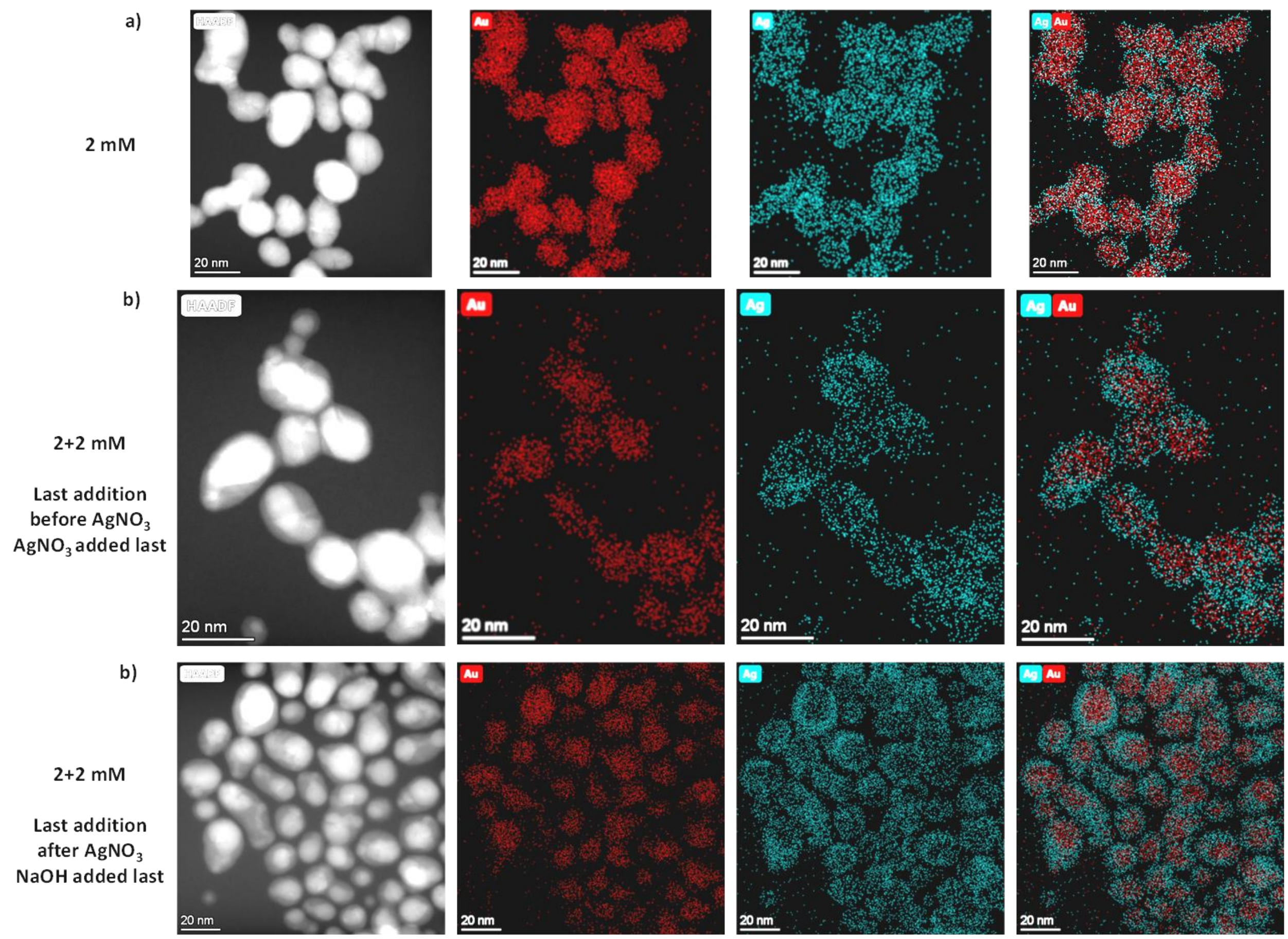

Figure S23. STEM-EDS micrographs for syntheses performed using 1.0 mM_{Au+Ag}, 30 v.% ethanol, a $HAuCl_4$:$AgNO_3$ molar ratio of 50:50 for a total concentration of (a) 2 mM and (b-c) 4 mM NaOH. The NaOH was added in such a way that (a) 2 mM of NaOH equivalent was added first, with 30 minutes delayed addition of $AgNO_3$, and (b-c) 2 steps addition of 2 mM each, with 30 minutes delayed addition of $AgNO_3$, with the base added before or after $AgNO_3$, as indicated. There was in all cases a 30 minutes delayed between the addition of the $HAuCl_4$ and $AgNO_3$ (a-c) and 20 seconds between the second addition of NaOH and $AgNO_3$ for (b-c).

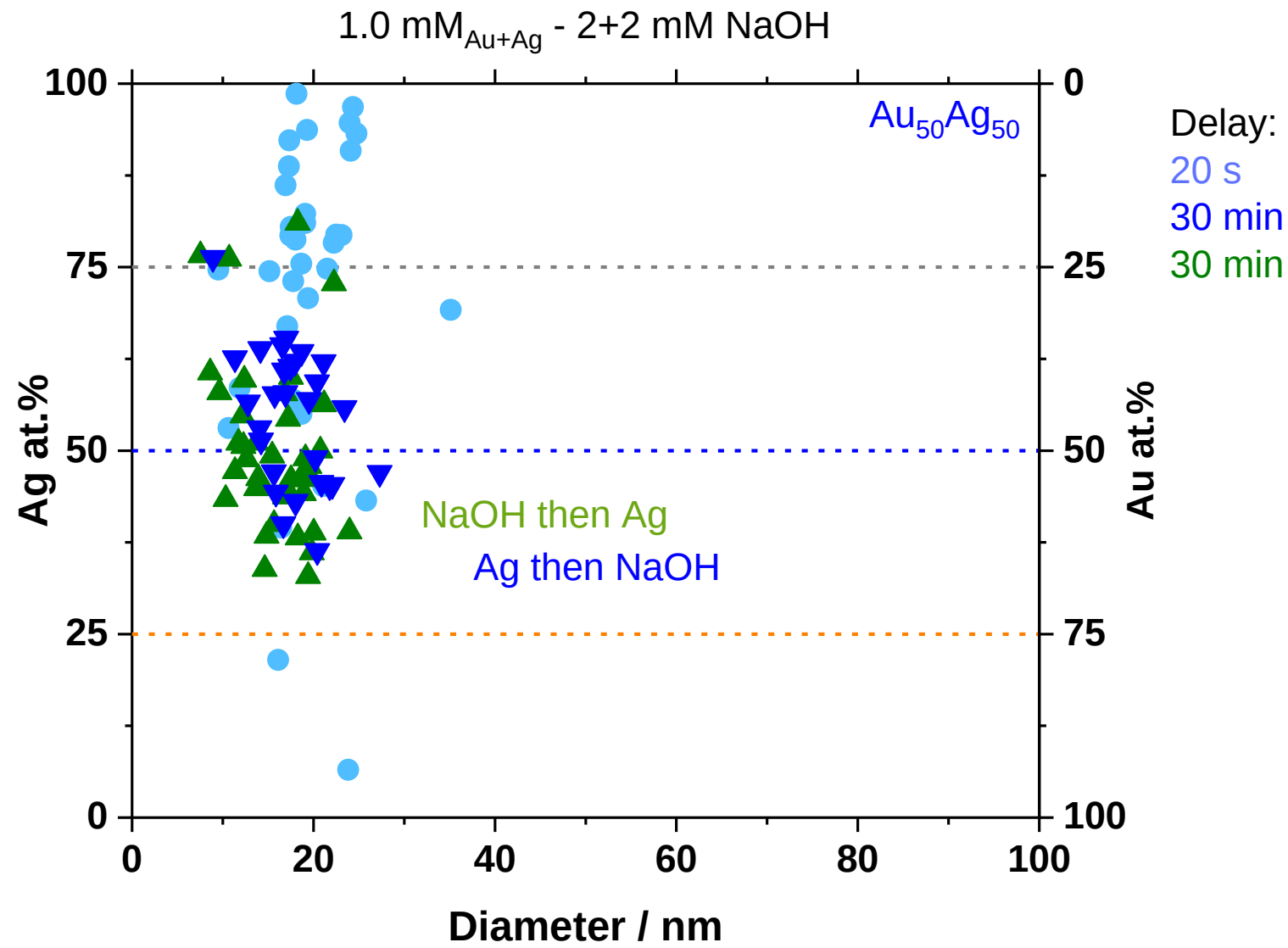

Figure S24. Size-composition relationship for the NPs prepared using 1.0 mM$_{Au+Ag}$, 30 v.% ethanol, a $HAuCl_4$:$AgNO_3$ molar ratio of 50:50 for a total concentration of 4 mM NaOH. The NaOH was added in a 2 steps addition of 2 mM with 30 minutes delayed addition of $AgNO_3$, with the base added before or after $AgNO_3$, as indicated. As a comparison a case where 4 mM NaOH was added at the beginning of the reaction and only 20 seconds delay between the addition of $HAuCl_4$ and $AgNO_3$ is presented.

A preference has been to add the $AgNO_3$ first and then the base with a 1 minute interval between $AgNO_3$. It is detailed below how this order in the sequence of addition overall lead to more reproducible results for Au@Ag NPs.

6. Core@Shell

The previous results suggest the possibility to prepare Au@Ag NPs by providing NaOH to a solution of forming Au NPs in ethanol. However, adding chemicals changes the concentration of the species and the Au:Ag molar ratio as the NPs form since the Au NPs might still be forming after 30-60 minutes.[2,5,14] We therefore investigated the opportunities to prepare Au@Ag NPs obtained using a pre-made solutions of seeds.

The NP seeds are characterized in **Figure S25**. The UV-vis spectra of the resulting Au@Ag NPs are reported in **Figure 2** in the manuscript. Due to the low stability of the samples prepared with seed synthesized using methanol for a Au:Ag molar ratio of 75:25 this sample was not considered further (see the low absorbance in the UV-vis spectrum in **Figure 2** of the main manuscript).

Table S4. Overview of the parameter space screened for the preparation of Au@Ag NPs using premade NPs.

**Alcohol**	**NaOH*** mM	**Precursor** mM_{Au+Ag}	**$HAuCl_4$:$AgNO_3$** molar ratio
Ethanol<br>Methanol	4	0.67<br>1<br>2	75:25<br>50:50<br>25:75

* The final NaOH concentration was preferentially achieved in 2 steps with an equal volume each time as detailed in the text, once to prepare the Au NPs seed and once before adding $AgNO_3$.

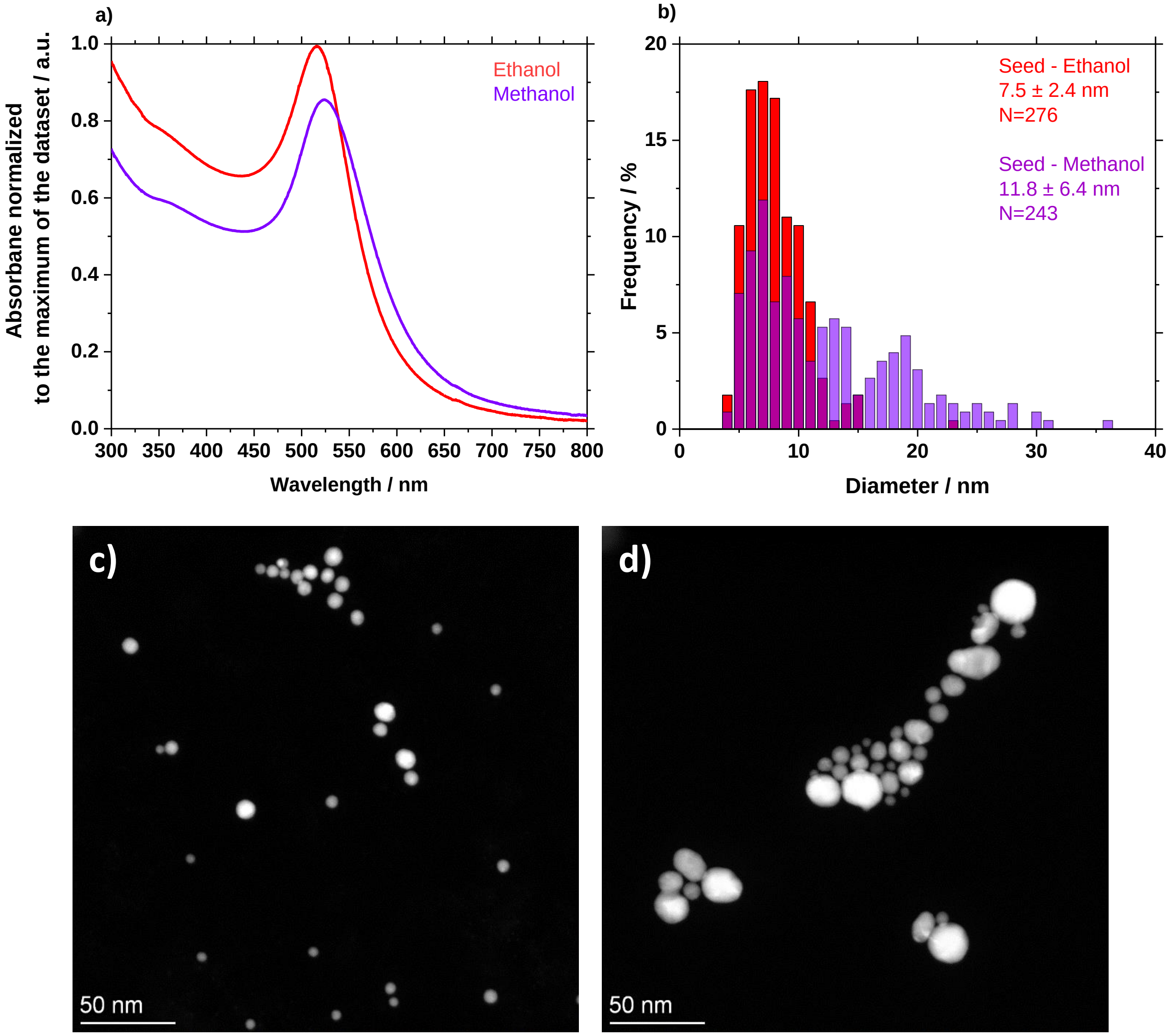

Figure S25. (a) Illustrative UV-vis of the Au NPs seeds prepared using 0.5 mM $HAuCl_4$, 2 mM NaOH and 30 v.% alcohol using ethanol or methanol, as indicated. (b) Corresponding size distribution. (c,d) Corresponding STEM micrographs. The UV-vis spectra of Au NPs obtained in ethanol is also reported in **Figure 1**.

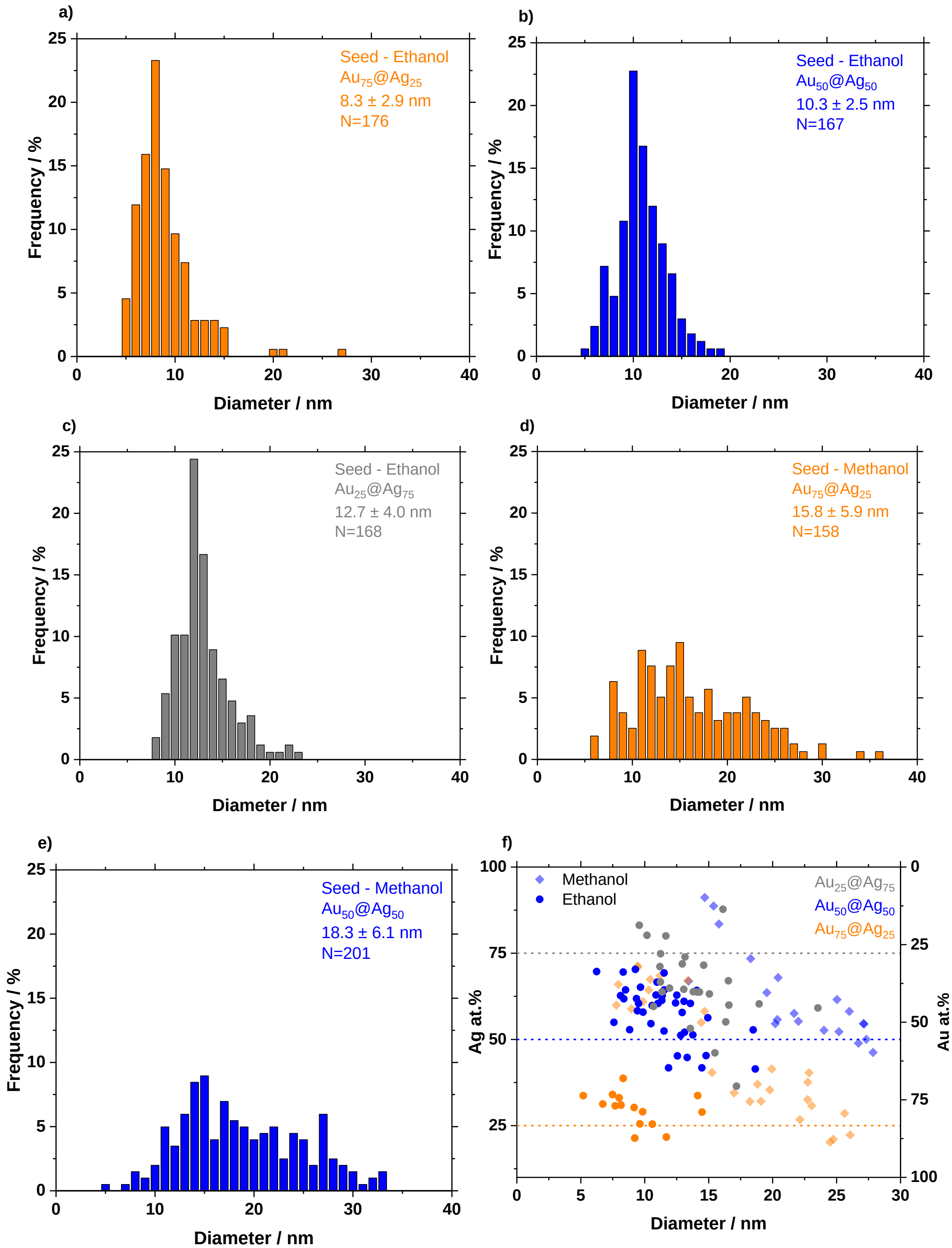

Figure S26. (a-e) Size distribution for Au@Ag NPs obtained using Au seeds at 0.5 mM_{Au} prepared in 30 v.% ethanol or methanol, as indicated. To the seed solutions, a desired amount of Ag was added to obtain Au:Ag molar ratios of 75:25, 50:50 and 75:25, as indicated. Overall 4 mM NaOH was used (in 2 steps addition) and $AgNO_3$ was added first then NaOH after 1 minute. The corresponding UV-vis spectra are reported in **Figure 2**. (f) Overall composition for individual NPs as a function of size.

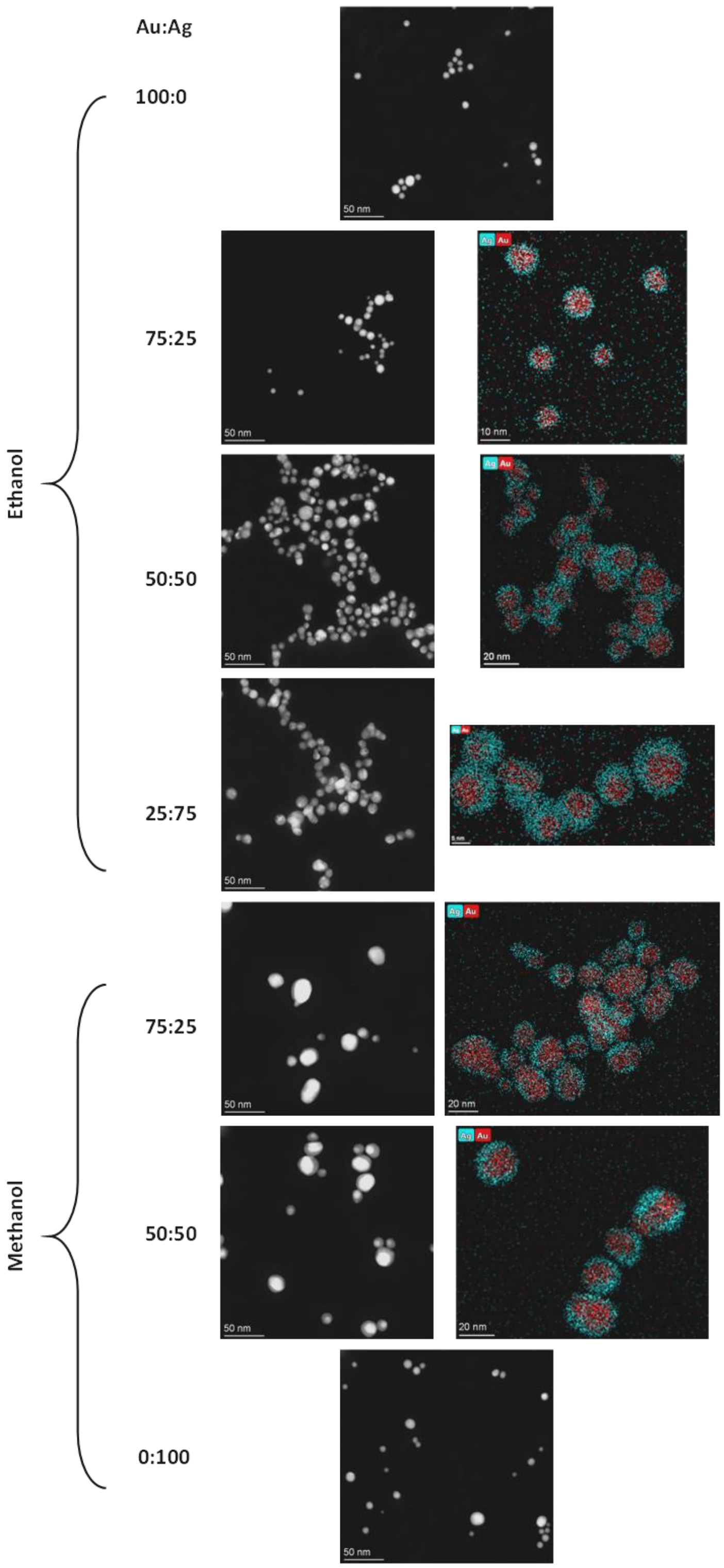

Figure S27. Complementary STEM-EDS data for Au@Ag NP obtained using Au seeds at 0.5 mM_{Au} prepared in ethanol or methanol, as indicated. To the seed solutions, a desired amount of Ag was added to obtain Au:Ag molar ratios of 75:25, 50:50 and 75:25, as indicated. Overall 4 mM NaOH was used (in 2 steps addition, $AgNO_3$ first then NaOH after 1 minute).

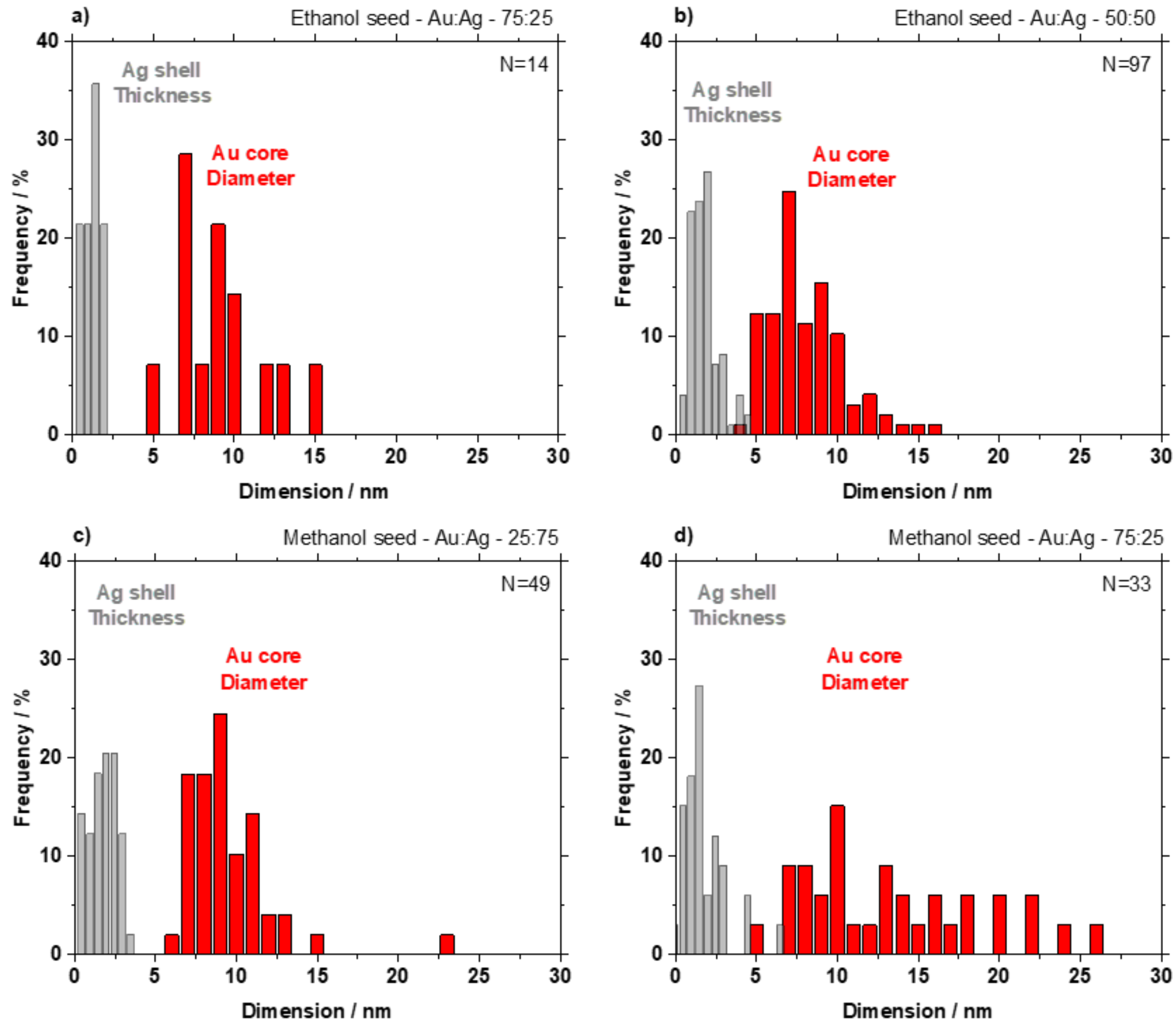

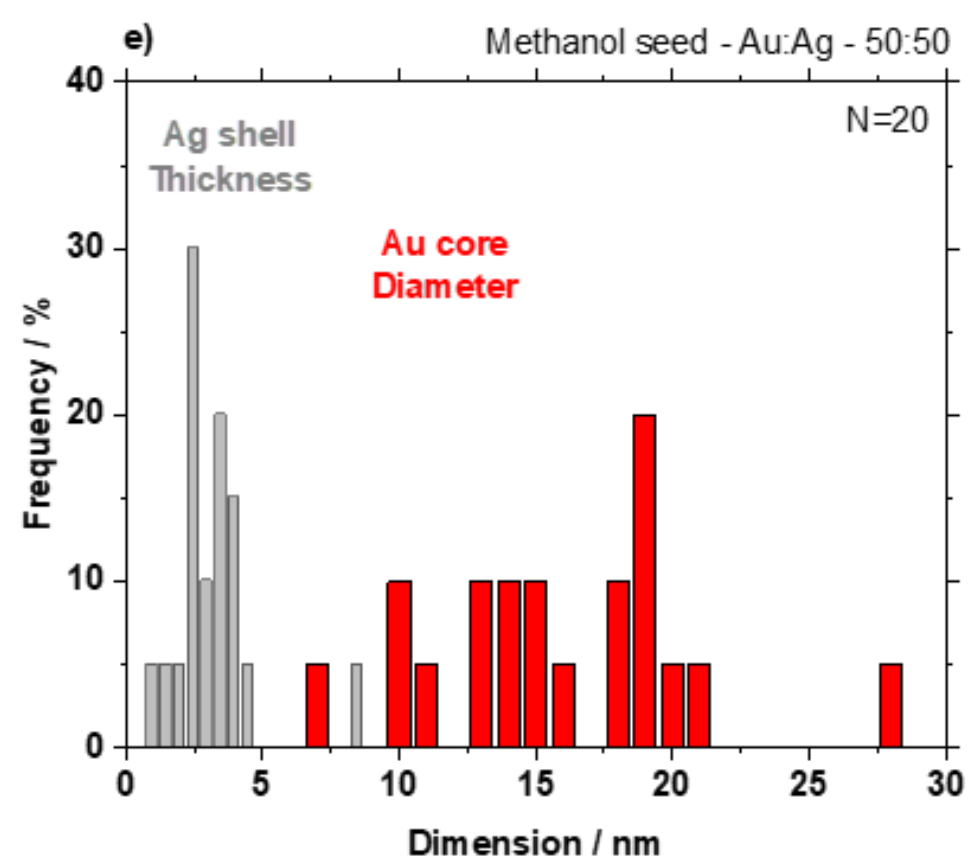

f)

Seed	Ag at.% Nominal	Ag at.% (deviation)
Ethanol	25	29.9 (4.77)
	50	57.88 (8.00)
	75	65.60 (11.2)
Methanol	25	45.74 (17.23)
	50	61.85 (13.08)

Figure S28. (a-e) Size distribution for Au@Ag NP obtained using Au seeds at 0.5 mM_{Au} prepared in 30 v.% ethanol or methanol, as indicated. To the seed solutions, a desired amount of Ag was added to obtain Au:Ag molar ratios of 75:25, 50:50 and 75:25, as indicated. Overall 4 mM NaOH was used (in 2 steps addition) and $AgNO_3$ was added first then NaOH after 1 minute. The corresponding UV-vis spectra are reported in **Figure 2**. The focus is here on the core and the shell dimensions retrieved from EDS maps for individual NPs. In case of thinner shells, the evaluation of the shell thickness can be challenging. (f) Overall EDS composition based on the results presented in **Figure S26d**.

A deviation between nominal and experimental composition is expected as in previous reports.[11,12] the mismatch can originate from deviation between the nominal and actual concentration of precursor, incomplete reduction of the precursors, limited statistics and/or limitation in the characterization technique.

On the need for NaOH and reproducibility

As detailed above, a preference was given to the addition of $AgNO_3$ first and then NaOH. We observed in some cases that if the NaOH is added first and $AgNO_3$ last, the reaction is less controlled. For instance, the very pronounced orange color in the case of a Au:Ag molar ratio of 1:1, was not always observed and the UV-vis spectra can significantly differ as illustrated in **Figure S29a**. For reference, the spectra of $AgNO_3$ in 20 v.% ethanol and $AgNO_3$ + NaOH in the concentration used here are also provided. It is confirmed that in the absence of Au NPs as seed, no clear Ag-like signal is observed.

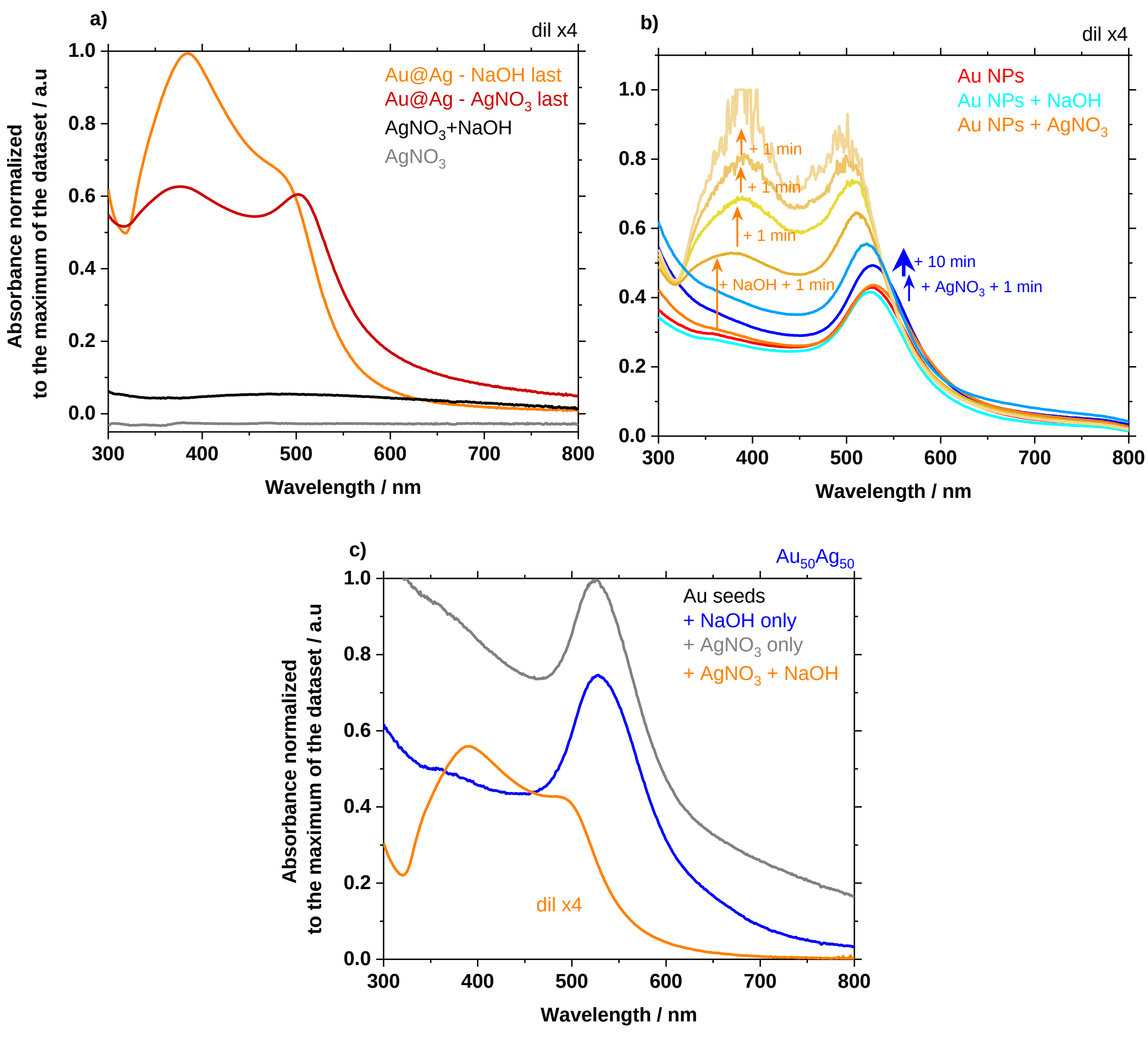

Figure S29. (a) UV-vis spectra of Au@Ag NPs obtained by adding NaOH or $AgNO_3$ last, as indicated. The spectra of a solution of $AgNO_3$ or $AgNO_3$+NaOH in concentrations and in solvent used for the syntheses are also reported for comparison. All solution were diluted by 4 for measurement. (b) UV-vis spectra of as prepared Au NPs (in red) and this same solution 1 minute after adding NaOH (light blue) or $AgNO_3$ (orange). In the case where $AgNO_3$ was added first, various spectra recorded with a 1 minute interval after adding NaOH are also presented and the

noisy signal observed is related to the saturation of the detector. In the case where NaOH was added first, the first spectra 1 minute after adding $AgNO_3$ is also reported as well as the spectra recorded after a further 10 minutes. The concentrations were 0.5 mM Au NPs, 0.5 mM $AgNO_3$ and 2+2 mM NaOH for 20 v.% ethanol in water. (c) UV-vis spectra measured a day after synthesis for Au NPs seeds (20 v.% ethanol, 0.5 mM_{Au}, 2 mM NaOH) to which were added NaOH (equivalent 2 mM), $AgNO_3$ (equivalent 0.5 mM_{Ag}) or both $AgNO_3$ and NaOH (successively with a 1 min delay).

This possible lower reproducibility observed depending on the order of addition of the chemicals is attributed to the different kinetics of reduction illustrated in **Figure S29b**. Adding $AgNO_3$ only (orange) or NaOH only (blue) to the Au seeds does not change much the spectra (as illustrated for 1 day after the addition of the chemicals but also even after a week). However, adding NaOH to a solution of Au NPs and $AgNO_3$ rapidly lead to spr features and an increased absorbance to a point that for the for concentrations used the signal saturates in less than 5 minutes. In contrast, adding $AgNO_3$ to a solution of Au NPs and NaOH leads to a more moderate change in the spectra and a moderate increase in absorbance even after 10 minutes. Those difference in kinetics are not explored further at this stage but are to be related to the redox properties of $AgNO_3$ in alkaline conditions and/or the need to slowly form a first layer of Ag on the Au NPs for the further successful growth of the shell. Furthermore, as detailed in the manuscript, the possible formation of the poorly soluble AgOH can be an additional challenge here.

Figure S29b and **Figure S29c** also confirm that NaOH is needed to lead to a relatively fast formation of the Au@Ag NPs and no clear signal indicative of Au@Ag structures could be observed in the presence of $AgNO_3$ alone without base. Note that no optimization of the time of synthesis for the shell formation was performed here assuming that the samples will be used at least the day after their preparation but **Figure S29b** confirm that the shell is formed within minutes with only minor and slow changes after 10-15 minutes.

A preference has been to add the $AgNO_3$ first and then the base with a 1 minute interval between $AgNO_3$ and NaOH.

7. Stability

Given that the NPs are here obtained without surfactant nor added stabilizers the NPs are very stable overtime for months whether stored at room temperature or in a fridge, as illustrated in **Figure S30**. The moderate change of the UV-vis signal with a more pronounced features at low wavelength around 400 nm suggests a change in the Ag shell overtime. Considering the fact that the NPs were stored at room temperature, those results suggest that the shell can be further controlled by temperature. Conveniently in this direction, the pre-formed NPs seed are stable at temperature up to 60-80 °C which opens opportunities for further control over the NP shell. The stability of the colloids is confirmed by the relatively high absolute value of 31 mV for zeta potential measurements, measured to be - 31 mV.

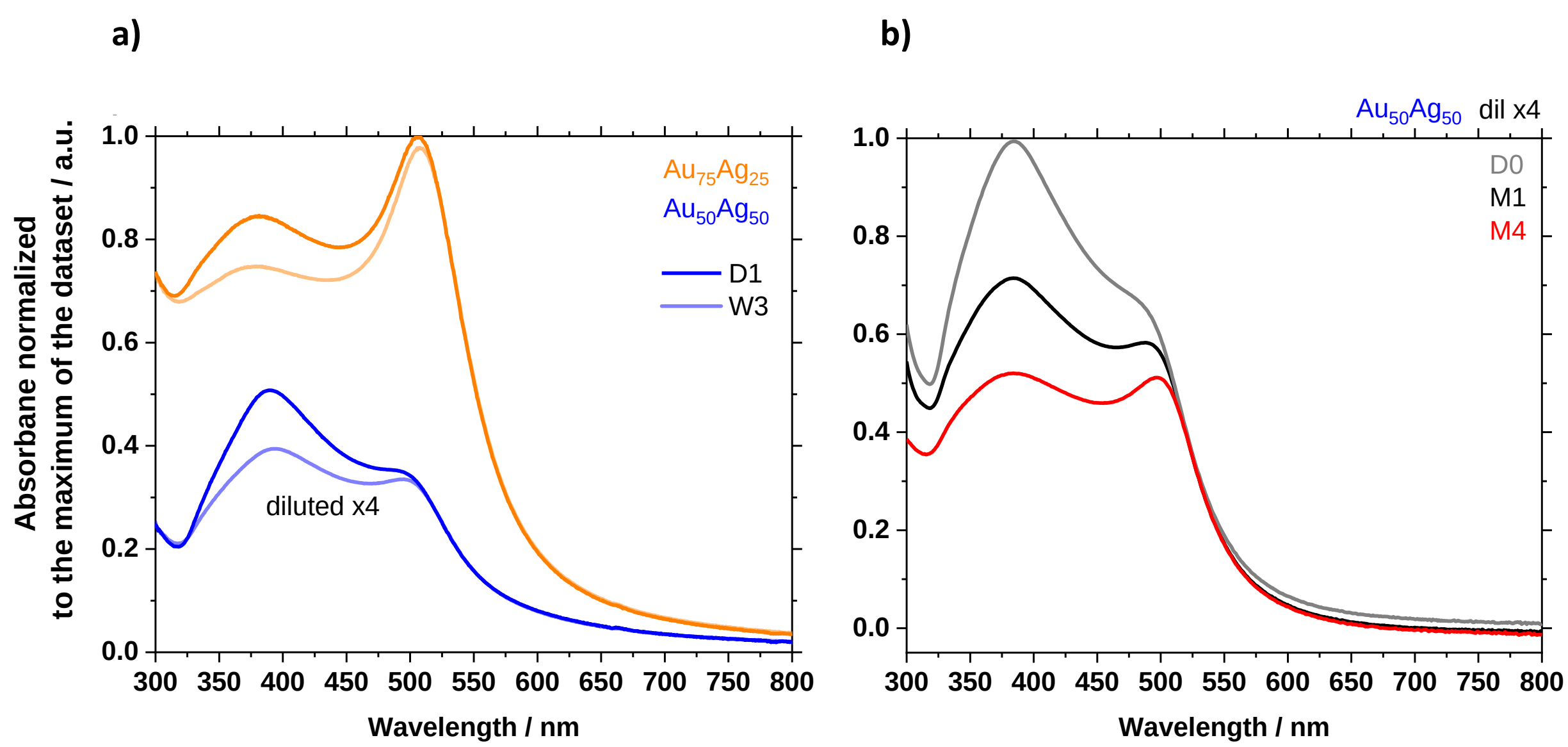

Figure S30. (a) UV-vis spectra of Au@Ag NPs seeds prepared using 0.5 mM $HAuCl_4$, overall 4 mM NaOH and 30 v.% methanol for different Au:Ag molar ratio of 75:25, or 50:50, as indicated. The NPs were stored at ambient temperatue ca. 22 °C. (b) UV-vis spectra of Au@Ag NPs seeds prepared using 0.5 mM $HAuCl_4$, overall 4 mM NaOH and 20 v.% ethanol for a Au:Ag molar ratio of 50:50. The NPs were stored in a fridge at ca. 6 °C. The spectra were recorded on the day of synthesis (D0), the day after (D1), 3 weeks after (W3), a month after (M1) or 4 months (M4) after synthesis.

8. Catalysis

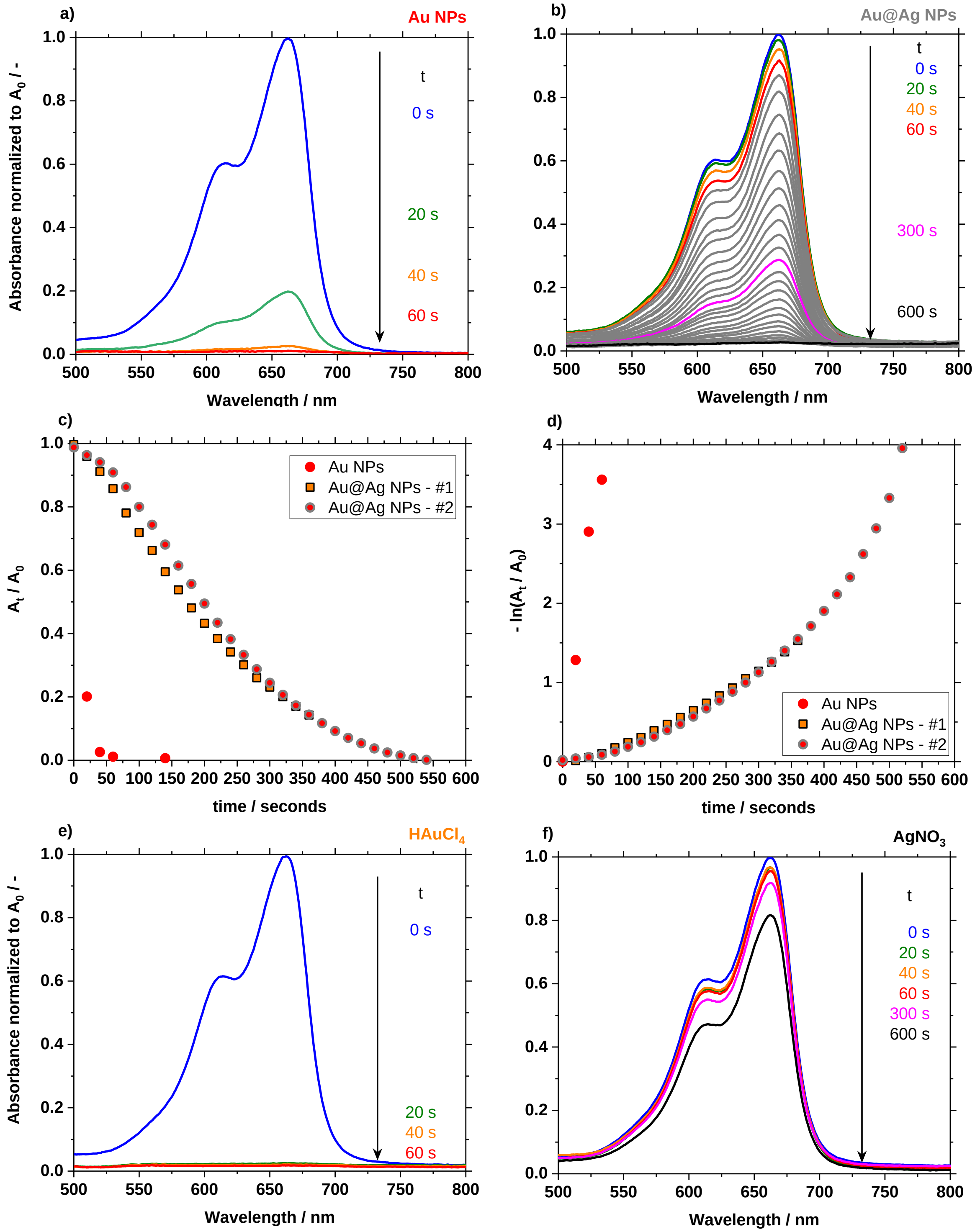

a)
Au NPs
Absorbance normalized to A0 / -
Wavelength / nm
t
0 s
20 s
40 s
60 s
b)
Au@Ag NPs
Wavelength / nm
t
0 s
20 s
40 s
60 s
300 s
600 s
c)
At / A0
time / seconds
Au NPs
Au@Ag NPs - #1
Au@Ag NPs - #2
d)
- ln(At / A0)
time / seconds
Au NPs
Au@Ag NPs - #1
Au@Ag NPs - #2
e)
HAuCl4
Absorbance normalized to A0 / -
Wavelength / nm
t
0 s
20 s
40 s
60 s
f)
AgNO3
Wavelength / nm
t
0 s
20 s
40 s
60 s
300 s
600 s

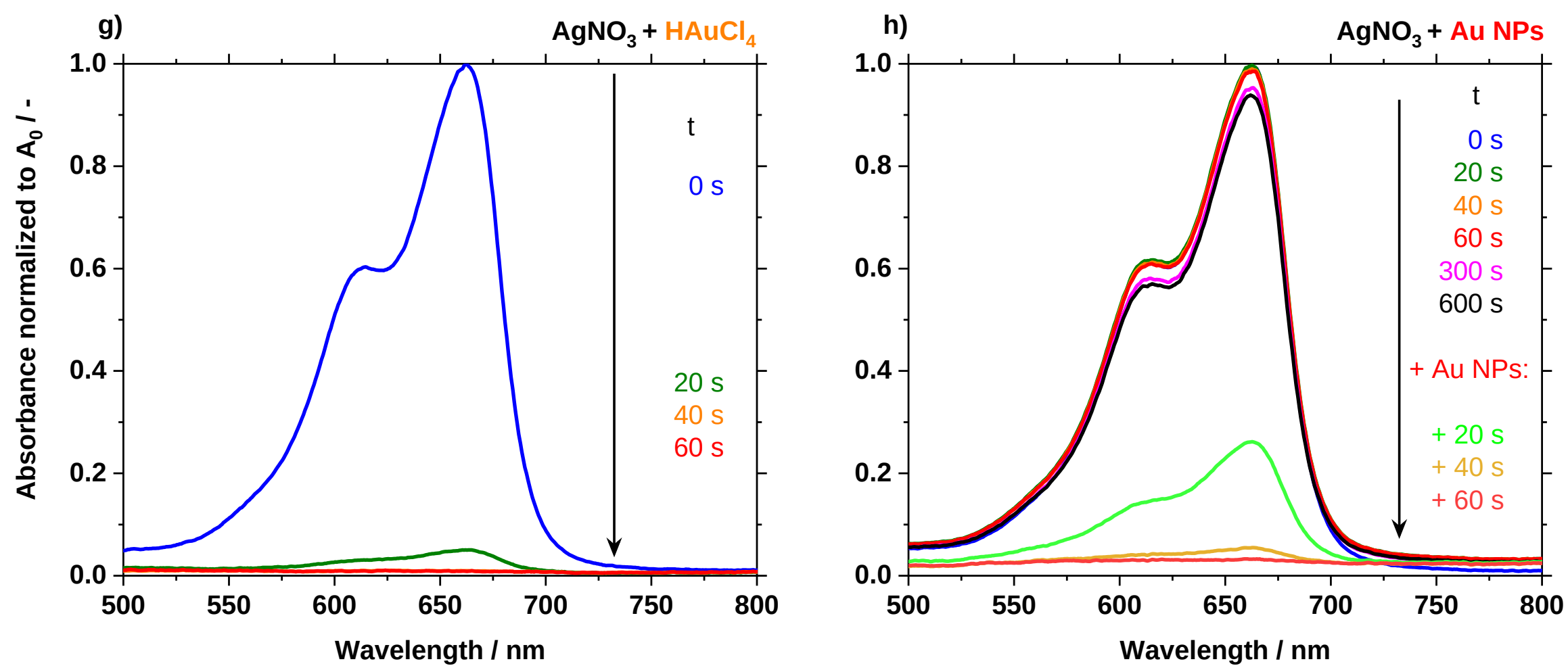

Figure S31. Illustrative examples of MB degradation catalyzed by various NPs and species and indicated. In all cases 10 µL of a solution of Au+Ag at 0.125 mM, was used. The catalyst used was (a) Au NPs or (b) Au@Ag NPs. The NPs were prepared in 20 v.% ethanol at 0.5 mM_{Au} and for a Au:Ag ratio of 1:0 or 1:1, and diluted x4 with water. (c) A_t/A_0 as a function of time. Two datasets for Au@Ag NPs are represented to illustrate the reproducibility of the measurements. (d) $-\ln(A_t/A_0)$ as a function of time. (e-f) Cases where 10 µL of a solution of (e) $HAuCl_4$ and (f) $AgNO_3$ at 0.125 mM in water were used. (g) Case where $AgNO_3$ was added rapidly followed by the addition of $HAuCl_4$ (10 uL each from a stock solution at 0.125 mM). (h) Case where 10 µL of a solution of $AgNO_3$ at 0.125 mM was added and after 600 s 10 µL of a solution of Au NPs at 0.125 mM.

The results in **Figure S31** illustrate the very fast degradation of MB in presence of Au NPs of when $HAuCl_4$ is directly added to the solution. When $HAuCl_4$ is directly added to the solution of MB it is expected that the $NaBH_4$ present in large excess contributes to the formation of Au NPs. The formation of a Ag shell changes the reaction kinetics significantly and this change cannot be attributed to potential leftover of unreacted $AgNO_3$ that might form Ag NPs *in situ*, **Figure S31f**, nor the formation of AuAg bimetallic which might happen in the cases presented in **Figure S31g** by co-reduction of Au and Ag precursors.[4] The control in **Figure S31h** where $AgNO_3$ was added before Au NPs were added also show the different behavior of the Au@Ag NPs. In the present case the silver shell limits the catalytic activity and enable to tune the reaction mechanism.

The MB reduction is a widely reported reaction to benchmark the catalytic properties of Au and Ag NPs. However, the wide range of syntheses methods reported, NP sizes, experimental conditions (volumes, concentrations, setup, etc.) make a comparison across the literature challenging, as illustrated in **Table S5**. Au-Ag based nanomaterials have been reported,[17,18] but there is surprisingly little work on Au@Ag NPs. We could not easily find a report focusing on this specific material. The results above therefore illustrate the potential of the present synthesis approach to fill gaps in the literature. Nevertheless, the Au@Ag NPs obtained here show a relatively high activity.

Table S5. Overview and illustration of catalytic assessment of Au and Ag NPs for MB $NaBH_4$-mediated reduction.

**Ref**	**Material**	**Size** nm	**$NaBH_4$** mM	**MB** mM	**Metal** mM	**k_{app}** min^{-1}	**TOF** min^{-1}
[19]	Ag-NP	20-30	29	0.18*	0.0006	0.26	25*
[20]	Ag-NP	10-30	33	0.03	0.06	2.6**	0.36
[21]	Au-NP	~30	4.5	0.04	-	0.006	-
[18]	Au-NR	~89 x 17	14	0.05	0.01	0.2	0.4
[18]	Au@Ag-NR	~100 x 34	14	0.05	0.02	0.1	0.1
**This work**	Au-NP	~10	10	0.05	0.000625	4.4**	113**
**This work**	Au@Ag-NP	~10+2	10	0.05	0.001250	0.4**	4.7**

NR: nanorods

* Based on the information provided but this quantity is suspiciously high for MB degradation (it will saturate most UV-vis spectrophotometers) and this high value account for the high TOF value.

** Considered one of the highest value but achieved at the expense of a large amount of metal

** The k_{app} and TOFF were evaluated for a conversion around or above 95% ($-\ln(A_t/A_0)$ around 3). In the case of Au@Ag the kinetics do not seem to follow a pseudo first-order kinetics, i.e. the k_{app} varies as a function of time and there seem to be a initial period where k_{app} is smaller (value around 0.1 min^{-1}) before it increases (final value around 1.0 min^{-1}).

The higher activity observed here, even for Ag surfaces, is attributed to the smallest size and the surfactant-free approach preferred here.

Similarly, the 4-NP reduction is a widely reported reaction to benchmark the catalytic properties of Au and Ag NPs. However, the wide range of syntheses methods reported, NP sizes, experimental conditions (volumes, concentrations, setup, etc.) make a comparison across the literature challenging,[11] as illustrated in **Table S6**. Nevertheless, the Au@Ag NPs obtained here show a relatively high activity.

Table S6. Overview and illustration of catalytic assessment of Ag NPs for 4-NP $NaBH_4$-mediated reduction.

**Ref**	**Material**	**Size** nm	**$NaBH_4$** mM	**4-NP** mM	**Metal** mM	**k_{app}** min^{-1}	**TOF** min^{-1}
[22]	Ag-NP	< 5	33	0.066	0.00007	58	-
[22]	Ag-NP	10-20	33	0.066	0.022	0.3	-
[23]	Ag-NP	18	12	0.12	0.05	0.33*	-
4**	Ag-NP	~20	30	0.14	0.0028	0.31*	40*
[16]	Ag-NP	~45	5	0.05	0.003750	0.15	0.6
**This work**	Au@Ag-NP	~10+2	10	0.05	0.001250	0.40	4.7

** The high values of k_{app} and TOFs can be attributed to the relatively high concentration of 4-NP used.[24]

* The authors note that the colloids are relatively unstable at high Ag contents in that study focusing on Au_xAg_{1-x} NPs.

9. Electrochemical characterization

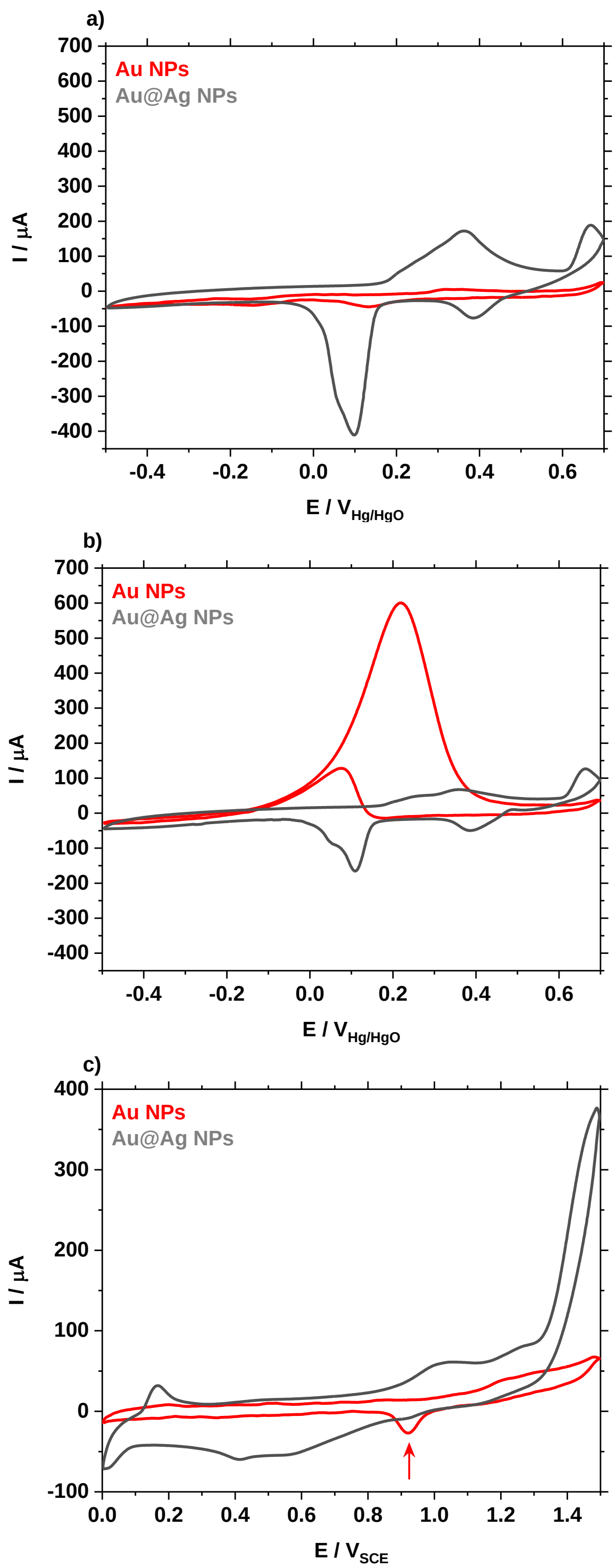

Figure S32. Illustrative cyclic voltammograms of Au NPs used as seeds (red) and Au@Ag NPs (Au:Ag of 50:50 molar ratios, grey). In (a) 1 M KOH, (b) in 1 M KOH + 1 M ethanol and (c) 0.5 M H_2SO_4.

The electrochemical characterization in **Figure S32** illustrates the impact of the Ag shell on the electrochemical properties of the Au NPs **Figure S32a** displays the CV in 1 M KOH of the Au and Au@Ag NPs. The Au@Ag NPs show more pronounced redox behaviour.[25] **Figure S32b** is presented in a such a way that the graph is directly comparable with **Figure S32a**, the only difference being the presence of ethanol in **Figure S32b**, and illustrate in the case of Au NPs the electrocatalytic oxidation of ethanol (EOR) detailed elsewhere and characterized by a large current around 0.3 $V_{Hg/HgO}$ and a significant current increase compared to **Figure S32a**.[3] The signal of the Au@Ag NPs remains the same in KOH or KOH + ethanol electrolytes with the same features at the same position (the small decrease in current is attributed to the rinsing and potential loss of materials by the previous CV performed). **Figure S32c** displays the typical CV of Au NPs in H_2SO_4 with a pronounced reduction peak around 0.9 V_{SCE} (indicated by a red arrow).[3] This characteristic peak disappears for the Au@Ag NPs whereas other redox behavior attributed to Ag are observed.[25]

All together, those results show that the surfactant-free NPs are readily electroactive. The results also show that the sample is not composed of Au and Ag NPs but that all (at least most) of the AuNPs are completely covered with Ag since no pronounced catalytic activity is observed for the Au@Ag NPs in **Figure S32b**. This result confirm further UV-vis and STEM-EDS data in the formation of Au@Ag NPs.

10. References

1 Quinson, J. On the Importance of Fresh Stock Solutions for Surfactant-Free Colloidal Syntheses of Gold Nanoparticles in Alkaline Alcohol and Water Mixtures. *Inorganics* **11**, 140 (2023). https://doi.org/10.3390/inorganics11040140

2 Rasmussen, D., Lock, N. & Quinson, J. Lights on the Synthesis of Surfactant-Free Colloidal Gold Nanoparticles in Alkaline Mixtures of Alcohols and Water. *ChemSusChem* **18**, e202400763 (2025). https://doi.org/10.1002/cssc.202400763

3 Panagopoulos, D., Alamdari, A. & Quinson, J. Surfactant-free colloidal gold nanoparticles: Room temperature synthesis, size control and opportunities for catalysis. *Materials Today Nano* **29**, 100600 (2025). https://doi.org/10.1016/j.mtnano.2025.100600

4 Larm, N. E., Thon, J. A., Vazmitsel, Y., Atwood, J. L. & Baker, G. A. Borohydride stabilized gold-silver bimetallic nanocatalysts for highly efficient 4-nitrophenol reduction. *Nanoscale Advances* **1**, 4665-4668 (2019). https://doi.org/10.1039/c9na00645a

5 Quinson, J. *et al.* Surfactant-Free Colloidal Syntheses of Gold-Based Nanomaterials in Alkaline Water and Mono-alcohol Mixtures. *Chemistry of Materials* **35**, 2173-2190 (2023). https://doi.org/10.1021/acs.chemmater.3c00090

6 Liz-Marzan, L. M., Kagan, C. R. & Millstone, J. E. Reproducibility in Nanocrystal Synthesis? Watch Out for Impurities! *ACS Nano* **14**, 6359-6361 (2020). https://doi.org/10.1021/acsnano.0c04709

7 Roy, A. *et al.* The Huge Role of Tiny Impurities in Nanoscale Synthesis. *ACS Nanoscience Au* **4**, 176-193 (2024). https://doi.org/10.1021/acsnanoscienceau.3c00056

8 Quinson, J. *et al.* Surfactant-free colloidal syntheses of gold-based nanomaterials in alkaline water and mono-alcohol mixtures. *Chemistry of Materials* **35**, 2173–2190 (2023). https://doi.org/10.1021/acs.chemmater.3c00090

9 Grazulis, S. *et al.* Crystallography Open Database (COD): an open-access collection of crystal structures and platform for world-wide collaboration. *Nucleic Acids Research* **40**, D420-D427 (2012). https://doi.org/10.1093/nar/gkr900

10 Jæger, F., Pedersen, A. A., Wacherhausen, P. S., Smolska, A. & Quinson, J. Surfactant-free gold nanoparticles synthesized in alkaline water-ethanol mixtures: leveraging lower grade chemicals for size control of active nanocatalysts. *RSC Sustainability* **3**, 2870-2875 (2025). https://doi.org/10.1039/D5SU00213C

11 Larm, N. E., Bhawawet, N., Thon, J. A. & Baker, G. A. Best practices for reporting nanocatalytic performance: lessons learned from nitroarene reduction as a model reaction. *New Journal of Chemistry* **43**, 17932-17936 (2019). https://doi.org/10.1039/c9nj01745c

12 Nathawat, P., Sahu, M. & Ganguly, M. Role of sodium borohydride as a co-catalyst for nanoparticle-induced degradation of toxic dyes in aquatic systems. *Nanoscale Advances* **8**, 1791-1816 (2026). https://doi.org/10.1039/d6na00073h

13 Quinson, J., Nielsen, T. M., Escudero-Escribano, M. & Jensen, K. M. Ø. Room temperature syntheses of surfactant-free colloidal gold nanoparticles: the benefits of mono-alcohols over polyols as reducing agents for electrocatalysis. *Colloids and Surfaces A: Physicochemical and Engineering Aspects* **675** 131853 (2023). https://doi.org/10.1016/j.colsurfa.2023.131853

14 Larsen, J., Porsgaard, A., Vinding, L., Smolska, A. & Quinson, J. Less chemicals for more controlled syntheses: on the benefits of mixtures of alcohols for room temperature surfactant-free colloidal syntheses of gold nanoparticles. *RSC Sustainability* **3**, 2876-2882 (2025). https://doi.org/10.1039/d5su00259a

15 Sorensen, M. *et al.* Room temperature surfactant-free syntheses of colloidal gold nanoparticles: Comparison of the precursors $HAuCl_4$ and $HAuBr_4$. *Colloids and Surfaces A-Physicochemical and Engineering Aspects* **739**, 140023 (2026). https://doi.org/10.1016/j.colsurfa.2026.140023

16 Lind, L. H. S., Smolska, A. & Quinson, J. Surfactant-free room temperature syntheses of stable colloidal silver nanoparticles in alkaline water-alcohols mixtures: pitfalls and opportunities. *Nanoscale Advances* **8**, 4781-4784 (2026). https://doi.org/10.1039/d6na00408c

17 Lee, H., Dao, A. & Kaneko, K. Volume-dependent catalytic efficiency of highly durable Au@Ag-Pt core@multi-shell nanoparticles in methylene blue reduction. *RSC Advances* **15**, 45928-45941 (2025). https://doi.org/10.1039/d5ra06393k

18 Zhang, Q. *et al.* Trimetallic Nanostructures of Silver-Platinum Alloy Shells on Gold Nanorods for Plasmon-Mediated Photocatalysis. *ACS Applied Nano Materials* **5**, 17048-17058 (2022). https://doi.org/10.1021/acsanm.2c03953

19 Almarashi, J. *et al.* Quick methylene blue dye elimination via SDS-Ag nanoparticles catalysts. *Scientific Reports* **14**, 15227 (2024). https://doi.org/10.1038/s41598-024-65491-6

20 Isa, N. & Lockman, Z. Methylene blue dye removal on silver nanoparticles reduced by Kyllinga brevifolia. *Environmental Science and Pollution Research* **26**, 11482-11495 (2019). https://doi.org/10.1007/s11356-019-04583-7

21 Stolle, H., Kluitmann, J., Csáki, A., Köhler, J. & Fritzsche, W. Shape-Dependent Catalytic Activity of Gold and Bimetallic Nanoparticles in the Reduction of Methylene Blue by Sodium Borohydride. *Catalysts* **11**, 1442 (2021). https://doi.org/10.3390/catal11121442

22 Kästner, C. & Thünemann, A. Catalytic Reduction of 4-Nitrophenol Using Silver Nanoparticles with Adjustable Activity. *Langmuir* **32**, 7383-7391 (2016). https://doi.org/10.1021/acs.langmuir.6b01477

23 Baruah, B., Gabriel, G., Akbashev, M. & Booher, M. Facile Synthesis of Silver Nanoparticles Stabilized by Cationic Polynorbornenes and Their Catalytic Activity in 4-Nitrophenol Reduction. *langmuir* **29**, 4225-4234 (2013). https://doi.org/10.1021/la305068p

24 Smolska, A., Lock, N. & Quinson, J. On the Overlooked Effect of Counter-Cations From Borohydrides for the Gold- and Metal- Mediated Catalytic Reduction of 4-Nitrophenol. *Small Methods* **10**, e02277 (2026). https://doi.org/10.1002/smtd.202502277

25 Tominaga, M. *et al.* Electrocatalytic oxidation of glucose at gold-silver alloy, silver and gold nanoparticles in an alkaline solution. *Journal of Electroanalytical Chemistry* **590**, 37-46 (2006). https://doi.org/10.1016/j.jelechem.2006.02.018
